# Cytosolic DNA inhibits rDNA transcription by retaining the RNA polymerase I transcription machinery

**DOI:** 10.1038/s44318-026-00792-2

**Published:** 2026-05-05

**Authors:** Yinfeng Xu, Qian Wang, Chuying Qian, Sheng Lu, Zhengfu He, Wei Liu, Wei Wan

**Affiliations:** 1 Laboratory of Basic Biology, Hunan First Normal University, Changsha, China; 2 Department of Thoracic Surgery of Sir Run Run Shaw Hospital, and Department of Biochemistry, Zhejiang University School of Medicine, Hangzhou, China; 3 Department of Metabolic Medicine, International Institutes of Medicine, the Fourth Affiliated Hospital, Zhejiang University School of Medicine, Yiwu, China

**Keywords:** Chromatin, Transcription & Genomics, Immunology, Microbiology, Virology & Host Pathogen Interaction

## Abstract

Cytosolic DNA, derived from cellular damage or microbial infection, functions as a pivotal trigger for the host innate immune responses by activating intracellular DNA-sensing machinery, including the cGAS-STING pathway. However, whether cytosolic DNA is involved in DNA-sensing pathway-independent biological processes remains largely unknown. Here, we show that cytosolic DNA interacts with UBTF and POLR1A, two essential components of the RNA polymerase I transcription machinery, and sequesters these two proteins in the cytoplasm. This retention decreases nuclear UBTF and POLR1A, inhibits rDNA transcription, suppresses protein synthesis, and curtails cell proliferation. Furthermore, we demonstrate that STING-induced autophagy specifically eliminates cytosolic DNA and restores nuclear UBTF and POLR1A, thereby abolishing the inhibitory effects of cytosolic DNA on rDNA transcription, protein synthesis, and cell proliferation. Thus, our findings uncover a novel role of cytosolic DNA in rDNA transcription, suggesting that cytosolic DNA not only activates immune responses but also interferes with cell metabolism.

## Synopsis

Cytosolic DNA, originating from cell damage or pathogen infection, is well recognized as an important inducer of innate immune responses. In this study, we demonstrate that cytosolic DNA retains the RNA polymerase I transcription machinery to inhibit rDNA transcription, protein synthesis, and cell proliferation, uncovering a previously unknown function of cytosolic DNA in cell metabolism.

- UBTF and POLR1A, two components of the RNA polymerase I transcription machinery, are identified as novel binding partners of cytosolic DNA.
- Cytosolic DNA sequesters UBTF and POLR1A in the cytoplasm, thereby reducing their nuclear abundance and inhibiting rDNA transcription.
- STING-induced autophagy restores rDNA transcription by selectively degrading cytosolic DNA and recovering nuclear UBTF and POLR1A.

## Abstract (web summary)

Cytosolic DNA traps UBTF and POLR1A in the cytoplasm to inhibit rDNA transcription and protein synthesis.

## Graphical abstract

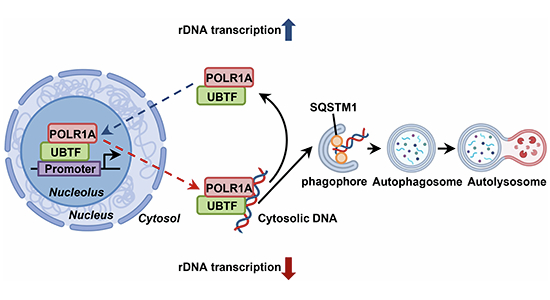

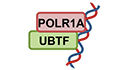

## Introduction

DNA, the carrier of genetic information for almost all life forms, has long been recognized as a strong inducer of innate immunity in mammals (Paludan and Bowie, 2013). In recent decades, the core DNA-sensing machinery that is responsible for such an immune response has been identified and characterized, including the cyclic GMP-AMP (cGAMP) synthase (cGAS)-stimulator of interferon genes (STING) pathway. Upon cellular damage or pathogen invasion, self or pathogen DNA is partially leaked into the cytoplasm and induces the innate immune responses of the cell (Barber, 2011; Gurtler and Bowie, 2013; Paludan and Bowie, 2013). In the cytoplasm, the enzyme cGAS is bound and activated by DNA and catalyzes the synthesis of the second messenger molecule cGAMP. Then, cGAMP directly binds to the adaptor protein STING that is localized at the endoplasmic reticulum (ER) and triggers the exit of STING from the ER. After that, STING traffics across the intracellular membrane compartments, including ERGIC, Golgi, endosome, and lysosome. Meanwhile, the protein kinases TANK-binding kinase 1 (TBK1) and IκB kinase (IKK) are recruited to STING to be activated, which then induce the nuclear translocation of transcription factors interferon regulatory factor 3 (IRF3) and nuclear factor κB (NF-κB), driving the expression of type I interferons (IFNs) and inflammatory cytokines (Chen and Xu, 2023; Chen et al, 2016; Zhang and Zhang, 2025). The innate immune responses, induced by DNA-sensing pathways, underpin many physiopathological processes triggered by cytosolic DNA, such as aging, neurodegeneration, and anti-viral and anti-tumor immunity (Decout et al, 2021; Dong and Fitzgerald, 2024; Miller et al, 2021). However, whether cytosolic DNA possesses DNA-sensing pathway-independent functions in the cell remains largely unknown.

In recent years, a number of novel functions of the cGAS-STING pathway have been discovered, several of which pre-date the emergence of the type I IFNs, such as induction of autophagy and activation of lysosome biogenesis (Gui et al, 2019; Huang et al, 2025; Liu et al, 2019; Lv et al, 2024a; Tapia et al, 2025; Wan et al, 2023; Xu et al, 2025). Autophagy is a highly conserved lysosome-dependent degradation process in eukaryotic cells, by which intracellular materials, such as protein aggregates, damaged organelles, and invading pathogens, are engulfed by double-membrane autophagosomes and delivered to lysosomes for digestion (Aman et al, 2021; Xu and Wan, 2023). It is worth noting that cytosolic DNA not only activates the cGAS-STING signaling but also undergoes autophagic degradation as the cargo during STING-induced autophagy (Xu and Wan, 2024; Xu and Wan, 2025). This seems to represent a delicate mechanism that ensures the timely elimination of cytosolic DNA, while avoiding excessive or prolonged immune responses.

In mammalian cells, all kinds of the mature ribosomal RNAs (rRNAs), accounting for the most cellular RNAs, include *18S*, *5.8S*, *28S*, and *5S* rRNAs. These rRNAs are the indispensable components of the ribosome, the only organelle for protein synthesis (Drygin et al, 2010; Hori et al, 2023). As a multistage process, ribosome biogenesis mainly takes place at the nucleolus, where the precursor rRNA transcript *47S* rRNA of *18S*, *5.8S*, and *28S* rRNAs is transcribed from the ribosomal DNA (rDNA) by the RNA polymerase I (Pol I) transcription machinery (Drygin et al, 2010; Hori et al, 2023). The first key event for rDNA transcription is the assembly of Pol I and other transcription-related proteins into a pre-initiation complex (PIC) at the rDNA promoter regions, which is required for the initiation of rDNA transcription (Drygin et al, 2010; Hori et al, 2023). The core components of the PIC at least include upstream binding transcription factor (UBTF), selectivity factor 1 (SL1), transcription initiation factor IA (TIF-IA), and Pol I (Russell and Zomerdijk, 2006). These components have been reported to be regulated by various post-translational modifications (Kusnadi et al, 2015; Sharifi and Bierhoff, 2018), which link intracellular and environmental cues to rDNA transcription activity. As rDNA transcription consumes tremendous cellular energy, its activity is highly linked to the energy and nutrient status of the cell, and tightly controlled by nutrient-sensing pathways. For instance, the energy sensor AMP-activated protein kinase (AMPK) and the nutrient sensor mechanistic target of rapamycin (mTOR) complex 1 (mTORC1) have been demonstrated to directly phosphorylate TIF-IA to control rDNA transcription in response to cellular energy supply and nutrient availability (Hoppe et al, 2009; Mayer et al, 2004). In addition to energy or nutrient deprivation, how rDNA transcription activity of the cell is regulated under stress conditions is poorly understood.

In this study, we identified UBTF and RNA polymerase I subunit A (POLR1A), a necessary subunit of Pol I, as novel binding proteins of cytosolic DNA. We showed that UBTF and POLR1A retention in the cytoplasm by cytosolic DNA leads to a significant decrease of these two proteins in the nucleus, which ultimately results in an inhibition of Pol I-mediated rDNA transcription. Furthermore, we demonstrated that pharmacological activation of STING-induced autophagy specifically promotes the elimination of cytosolic DNA, which abrogates the decease of nuclear UBTF and POLR1A and the reduction of rDNA transcription activity. Functionally, we unraveled that cytosolic DNA inhibits protein synthesis and cell proliferation, and these inhibitory effects can be abolished by STING-induced autophagy.

## Results

### Identification of UBTF and POLR1A as novel binding proteins of cytosolic DNA

**Figure 1:**
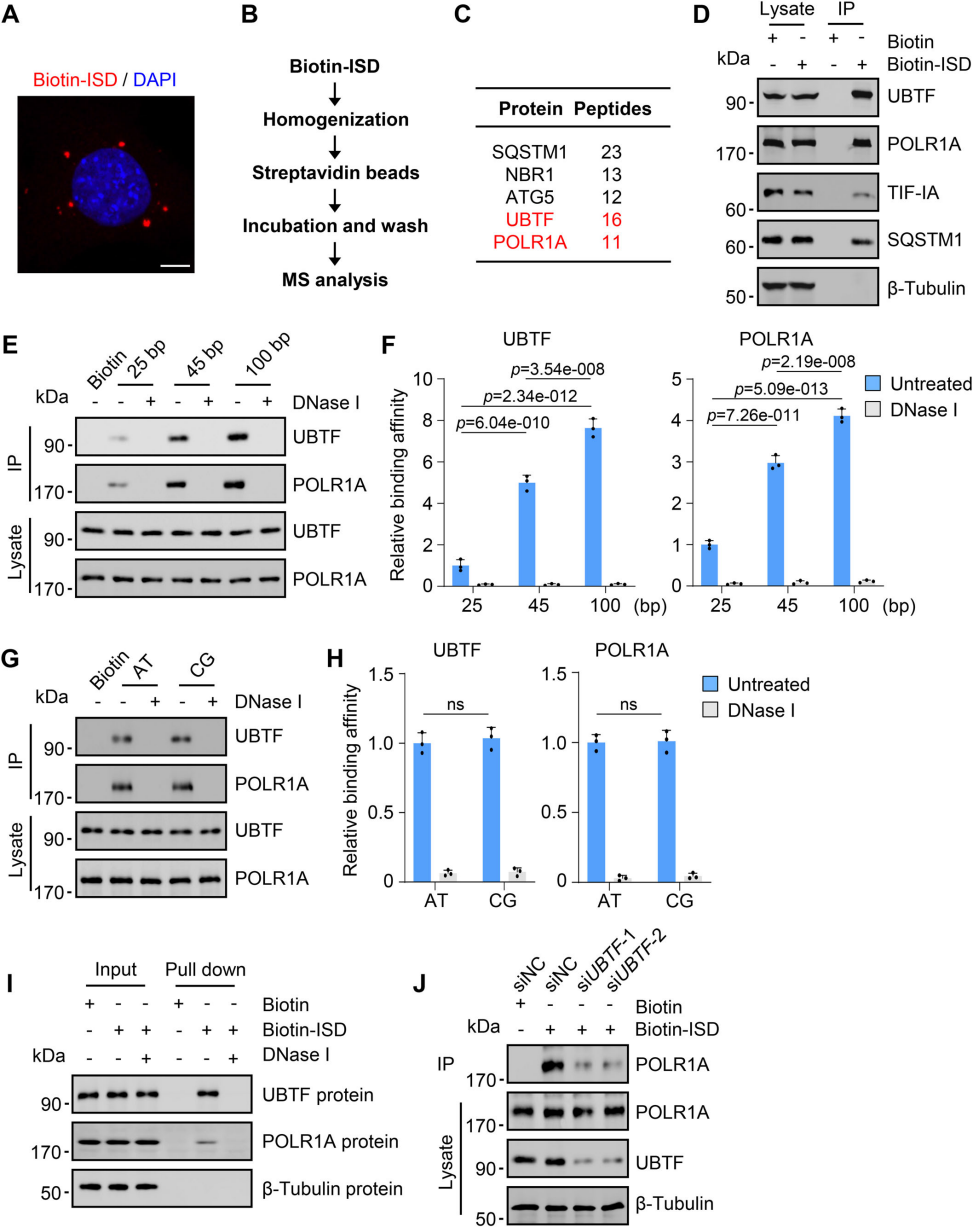
Identification of UBTF and POLR1A as novel binding proteins of cytosolic DNA. (**A**) Subcellular localization of biotin-labeled interferon stimulatory DNA (biotin-ISD) in mouse embryonic fibroblasts (MEFs). MEFs transfected with biotin-ISD were subjected to immunostaining using anti-biotin. Scale bars, 10 μM. (**B**) Schematic of the workflow of the screening method for identification of binding proteins of cytosolic biotin-ISD by mass spectrometric analysis. (**C**) The highly abundant proteins pulled down by biotin-ISD from MEFs transfected with biotin-ISD. (**D**) Association of endogenous UBTF and POLR1A with biotin-ISD. Biotin-ISD was pulled down using streptavidin beads from MEFs transfected with biotin-ISD, and the bound proteins were subjected to western blot analysis using anti-UBTF, anti-POLR1A, anti-TIF-IA, anti-SQSTM1, and anti-β-Tubulin. (**E**) Binding of biotin-labeled DNA fragments to endogenous UBTF and POLR1A. Cells were transfected with biotin-labeled DNA fragments with different lengths. The cell lysates were incubated with or without DNase I. Following that, the resulting cell lysates were incubated with streptavidin beads and the bound proteins were subjected to western blot analysis. (**F**) Statistical analysis of (**E**). (**G**) Binding of biotin-labeled DNA fragments to endogenous UBTF and POLR1A. Cells were transfected with biotin-labeled DNA fragments with different base pairs. (**H**) Statistical analysis of (**G**). (**I**) Interaction between biotin-ISD and recombinant UBTF or POLR1A protein. Biotin-ISD was incubated with recombinant UBTF, POLR1A, or β-Tubulin and pulled down using streptavidin beads, and the bound proteins were subjected to western blot analysis using anti-UBTF, anti-POLR1A, or anti-β-Tubulin. (**J**) Interaction between POLR1A and biotin-ISD. Cells were treated with or without *UBTF* siRNAs and then transfected with biotin-ISD. Following that, biotin-ISD was pulled down and the bound proteins were analyzed by western blot using anti-POLR1A. All statistical data are presented as mean ± SD of three independent experiments and analyzed by two-way ANOVA and Holm–Sidak post hoc test. ns, not significant. Source data are available online for this figure.

To investigate whether cytosolic DNA in the cell plays roles in DNA-sensing pathway-independent processes, we started by identifying the binding proteins of cytosolic DNA within cells using biotin-labeled-interferon stimulatory DNA (ISD) (biotin-ISD) (Stetson and Medzhitov, 2006). Consistent with previous studies (Gui et al, 2019; Wan et al, 2023; Xu et al, 2024), biotin-ISD formed punctate structures in the cytoplasm of mouse embryonic fibroblasts (MEFs) (Fig. 1A). Using biotin-ISD in the cytoplasm as the bait, MEFs transfected with biotin-ISD were homogenized and incubated with streptavidin beads, and the proteins binding to isolated biotin-ISD were analyzed by mass spectrometry (Fig. 1B). The proteins pulled down by biotin-ISD included several autophagy-related proteins (Fig. 1C), which have been reported to function in STING-induced autophagy (Gui et al, 2019; Prabakaran et al, 2018). Intriguingly, UBTF and POLR1A, two essential components of the RNA Pol I transcription machinery (Drygin et al, 2010; Hori et al, 2023; Russell and Zomerdijk, 2006), were also highly abundant in the precipitates (Fig. 1C).

**Figure EV1:**
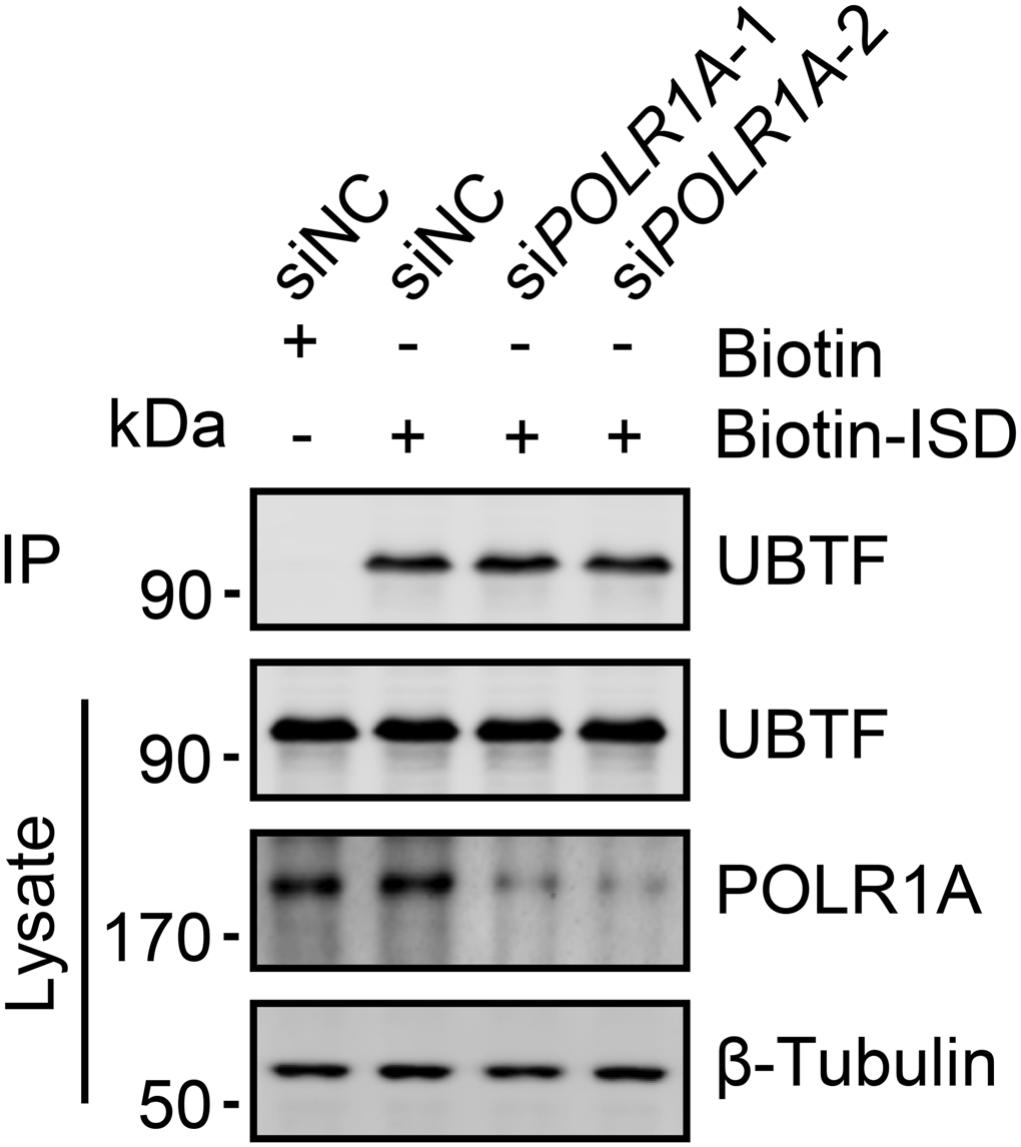
POLR1A is not required for the interaction between UBTF and ISD. Cells were treated with or without *POLR1A* siRNAs and then transfected with biotin-ISD. Following that, biotin-ISD was pulled down and the bound proteins were analyzed by western blot using anti-UBTF. Source data are available online for this figure.

To verify the screening result, we pulled down biotin-ISD from cells and subjected the precipitates to western blot analysis. As expected, biotin-ISD co-precipitated UBTF, POLR1A, TIF-IA, another component of the RNA Pol I transcription machinery, and autophagy-related protein sequestosome 1 (SQSTM1), but not cytosolic protein β-Tubulin (Fig. 1D). In addition, when transfecting cells with biotin-labeled DNA fragments with different lengths, we found that both of them co-precipitated UBTF and POLR1A (Fig. 1E). However, treatment of the precipitates with DNase I to cleave DNA completely abolished the co-precipitation of UBTF and POLR1A (Fig. 1E). Of note, longer DNA fragments co-precipitated higher amount of UBTF and POLR1A (Fig. 1E,F). These data suggest that cytosolic DNA binds to UBTF and POLR1A through a length-dependent manner. We also checked the binding using synthesized DNA fragments composed of only adenine and thymine, or cytosine and guanine. Interestingly, these two distinct DNA fragments exhibited similar affinity to UBTF and POLR1A (Fig. 1G,H), suggesting that cytosolic DNA appears to bind to UBTF and POLR1A through a base pair-independent manner. The mass spectrometric analysis showed a higher number of UBTF peptides in the precipitates pulled down by cytosolic DNA compared to POLR1A (Fig. 1C), prompting us to test whether UBTF is the key factor mediating the association of cytosolic DNA with the RNA Pol I transcription machinery. By using recombinant proteins, we showed that ISD can directly bind to both UBTF and POLR1A in vitro (Fig. 1I). Notably, ISD exhibited a much higher affinity for UBTF than for POLR1A (Fig. 1I). Furthermore, we found that knockdown of *UBTF* can markedly weaken the interaction between ISD and POLR1A in cells (Fig. 1J). Conversely, knockdown of *POLR1A* did not impair the association of ISD with UBTF (Fig. EV1).

Taken together, these data suggest that cytosolic DNA interacts with the key components of the RNA Pol I transcription machinery in the cytoplasm.

### Cytosolic DNA triggers the subcellular redistribution of UBTF and POLR1A

**Figure 2:**
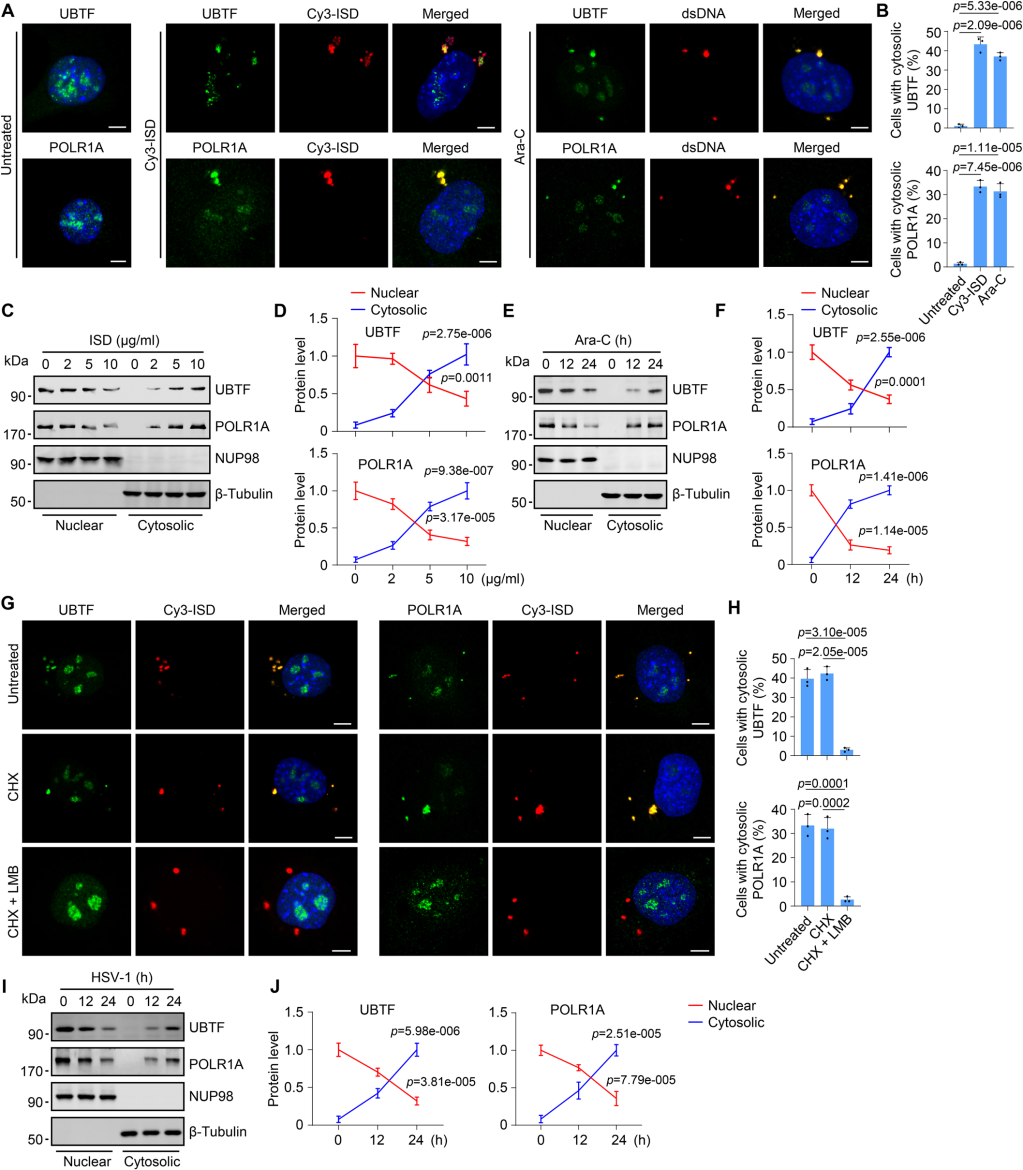
Cytosolic DNA triggers the subcellular redistribution of UBTF and POLR1A. (**A**) Subcellular localization of UBTF and POLR1A. U2OS cells were transfected with Cy3 labeled ISD (Cy3-ISD) or treated with arabinofuranosyl cytidine (Ara-C), a chemical that causes DNA damage and leads to an accumulation of endogenous DNA in the cytoplasm. After the treatments, all cells were stained using anti-UBTF, anti-POLR1A, or anti-dsDNA. (**B**) Statistical analysis of cells with cytosolic UBTF or POLR1A treated as in (**A**). (**C**) The protein levels of UBTF and POLR1A in the nuclear and cytoplasmic fractions of MEFs. Cells were transfected with ISD with different concentrations as indicated. (**D**) Statistical analysis of the protein levels of UBTF and POLR1A in the nuclear and cytoplasmic fractions from cells treated as in (**C**). The relative levels of nuclear UBTF and POLR1A were normalized to NUP98, and the relative levels of cytoplasmic UBTF and POLR1A were normalized to β-Tubulin. (**E**) The protein levels of UBTF and POLR1A in the nuclear and cytoplasmic fractions of MEFs. Cells were treated with Ara-C for different time as indicated. (**F**) Statistical analysis of the protein levels of UBTF and POLR1A in the nuclear and cytoplasmic fractions from cells treated as in (**E**). (**G**) Subcellular localization of UBTF and POLR1A in MEFs. Cells were incubated with cycloheximide (CHX), a protein synthesis inhibitor, or co-treated with leptomycin B (LMB), a nuclear export inhibitor of proteins. Following that, the cells were transfected with Cy3-ISD and subjected to immunostaining using anti-UBTF or anti-POLR1A. (**H**) Statistical analysis of cells with cytosolic UBTF or POLR1A treated as in (**G**). (**I**) The protein levels of UBTF and POLR1A in the nuclear and cytoplasmic fractions of MEFs. Cells were infected with HSV-1 for different time as indicated. (**J**) Statistical analysis of cells with cytosolic UBTF or POLR1A treated as in (**I**). All statistical data are presented as mean ± SD of three independent experiments and analyzed by one-way ANOVA and Tukey’s post hoc test. Scale bars, 10 µm. Source data are available online for this figure.

As the essential components of Pol I transcription machinery, UBTF and POLR1A are mainly localized at the nucleolus, where rDNA transcription takes place (Drygin et al, 2010; Hori et al, 2023; Russell and Zomerdijk, 2006). Consistent with this, endogenous UBTF and POLR1A were mainly distributed at the nucleolus under normal conditions (Fig. 2A,B). Intriguingly, a portion of UBTF and POLR1A was localized in the cytoplasm of cells transfected with Cy3 labeled ISD (Cy3-ISD) (Fig. 2A,B). Moreover, both UBTF and POLR1A in the cytoplasm were targeted to Cy3-ISD-positive punctuate structures (Fig. 2A,B), suggesting that these two proteins might be retained in the cytoplasm by cytosolic DNA. To test whether endogenous cytosolic DNA can also sequester UBTF and POLR1A, we used arabinofuranosyl cytidine (Ara-C), which interferes with DNA synthesis to cause DNA damage, eventually leading to DNA leakage into the cytoplasm (Grant, 1998; Gui et al, 2019). To specifically stain cytosolic DNA, we used a low-permeabilization buffer that allows the antibody to pass through plasma membrane, but not mitochondrial membrane or nuclear envelope (Spada et al, 2019) (Appendix Fig. S1). Consistent with the previous observations (Gui et al, 2019; Wan et al, 2023), a portion of endogenous DNA was distributed in the cytoplasm upon Ara-C treatment (Fig. 2A,B). Moreover, a portion of UBTF and POLR1A also appeared in the cytoplasm and targeted to endogenous cytosolic DNA (Fig. 2A,B).

**Figure EV2:**
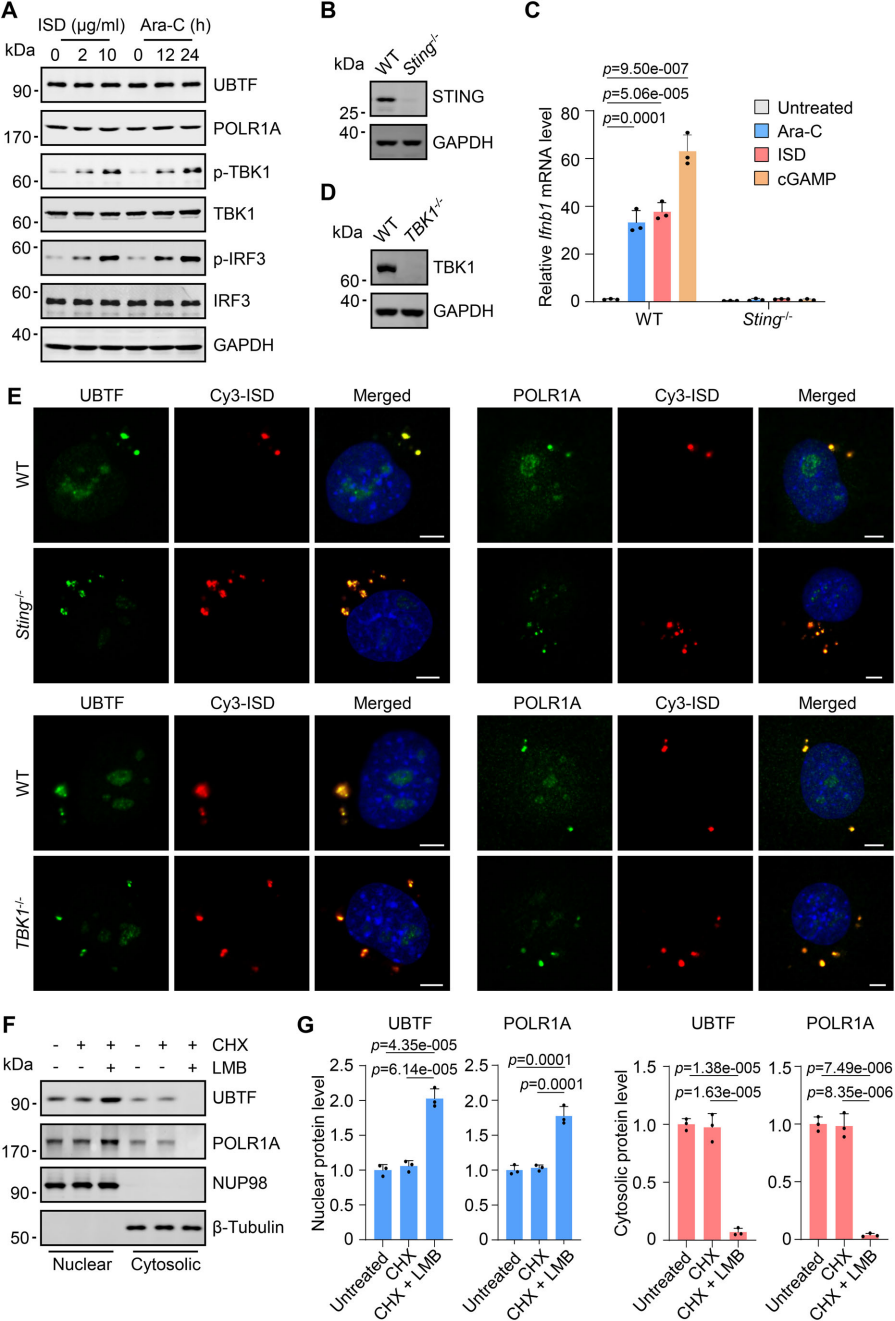
The cGAS-STING signaling is not required for the retention of UBTF and POLR1A in the cytoplasm by cytosolic DNA. (**A**) The protein levels of UBTF and POLR1A in MEFs transfected with ISD or treated with arabinofuranosyl cytidine (Ara-C) as indicated. (**B**) Verification of *Sting* knockout MEFs. (**C**) Statistical analysis of *Ifnb1* mRNA levels in wild-type (WT) and *Sting*^−/−^ MEFs. The cells were treated as indicated. (**D**) Verification of *TBK1* knockout DLD1 cells. (**E**) Subcellular localization of UBTF and POLR1A in cells transfected with Cy3 labeled ISD (Cy3-ISD). WT and *Sting*^−/−^ MEFs, or WT and *TBK1*^−/−^ DLD1 cells, were transfected with Cy3-ISD and subjected to immunostaining using anti-UBTF and anti-POLR1A, respectively. Scale bars, 10 µm. (**F**) The protein levels of UBTF and POLR1A in the nuclear and cytoplasmic fractions of MEFs. Cells were incubated with cycloheximide (CHX), a protein synthesis inhibitor, or co-treated with leptomycin B (LMB), a nuclear export inhibitor of proteins. Following that, the cells were transfected with Cy3-ISD and subjected to cell fractionation experiments. (**G**) Statistical analysis of the protein levels of UBTF and POLR1A in the nuclear and cytoplasmic fractions from cells treated as in (**F**). The relative levels of nuclear UBTF and POLR1A were normalized to NUP98, and the relative levels of cytoplasmic UBTF and POLR1A were normalized to β-Tubulin. All statistical data are presented as mean ± SD of three independent experiments and analyzed by one-way ANOVA and Tukey’s post hoc test. Source data are available online for this figure.

To further verify the retention of UBTF and POLR1A in the cytoplasm by cytosolic DNA, we performed cell fractionation experiments and confirmed that both exogenous and endogenous cytosolic DNA can lead to the appearance of UBTF and POLR1A in the cytoplasm (Fig. 2C–F). Meanwhile, the nuclear levels of UBTF and POLR1A were significantly decreased (Fig. 2C–F). We also examined cellular levels of UBTF and POLR1A in cells with ISD transfection or Ara-C treatment, and found the protein levels of these two proteins in cells remained unaffected (Fig. EV2A). These data together indicate that UBTF and POLR1A in the cytoplasm under these conditions might be from the nucleus. Of note, the phosphorylation of TBK1 and IRF3 in these cells was markedly upregulated (Fig. EV2A), supporting the notion that cytosolic DNA is an inducer of the cGAS-STING signaling. Consistent with this, both ISD transfection and Ara-C treatment upregulated *Ifnb1* mRNA levels in wild-type (WT) rather than *Sting* knockout cells (Fig. EV2B,C). To determine whether the cGAS-STING signaling affects the subcellular distribution of UBTF and POLR1A, we utilized cells without STING or TBK1 (Xu et al, 2025) (Fig. EV2B,D). In these cells, both UBTF and POLR1A can still be sequestered in the cytoplasm by Cy3-ISD (Fig. EV2E), ruling out the direct involvement of the cGAS-STING pathway in this process.

To validate whether UBTF and POLR1A in the cytoplasm indeed originate from the nucleus, we utilized cycloheximide (CHX), a protein synthesis inhibitor, and leptomycin B (LMB), a nuclear protein export inhibitor. Cells were treated with CHX and followed by Cy3-ISD transfection, we found that UBTF and POLR1A were still targeted to Cy3-ISD in the cytoplasm (Fig. 2G,H), indicating that they may not be from the nascent proteins translated in the cytoplasm. However, when cells were treated with both CHX and LMB and then transfected with Cy3-ISD, UBTF and POLR1A were no longer sequestered in the cytoplasm by cytosolic Cy3-ISD (Fig. 2G,H). In addition, we utilized these cells to carry out cell fractionation experiments and obtained consistent results (Fig. EV2F,G). These results suggest that UBTF and POLR1A bound to cytosolic Cy3-ISD are indeed from the nucleus. Given that pathogen DNA can appear in the cytoplasm of the host cells during pathogen infection, we investigated the effect of DNA virus herpes simplex virus-1 (HSV-1) infection on the nuclear-cytoplasmic distribution of UBTF and POLR1A. As expected, HSV-1 infection caused the appearance of UBTF and POLR1A in the cytoplasm and decreased the nuclear levels of these two proteins (Fig. 2I,J).

These data together suggest that cytosolic DNA sequesters UBTF and POLR1A in the cytoplasm and leads to a reduction of them in the nucleus.

### Cytosolic DNA-mediated retention of UBTF and POLR1A in the cytoplasm is abrogated by STING-induced autophagy

**Figure 3:**
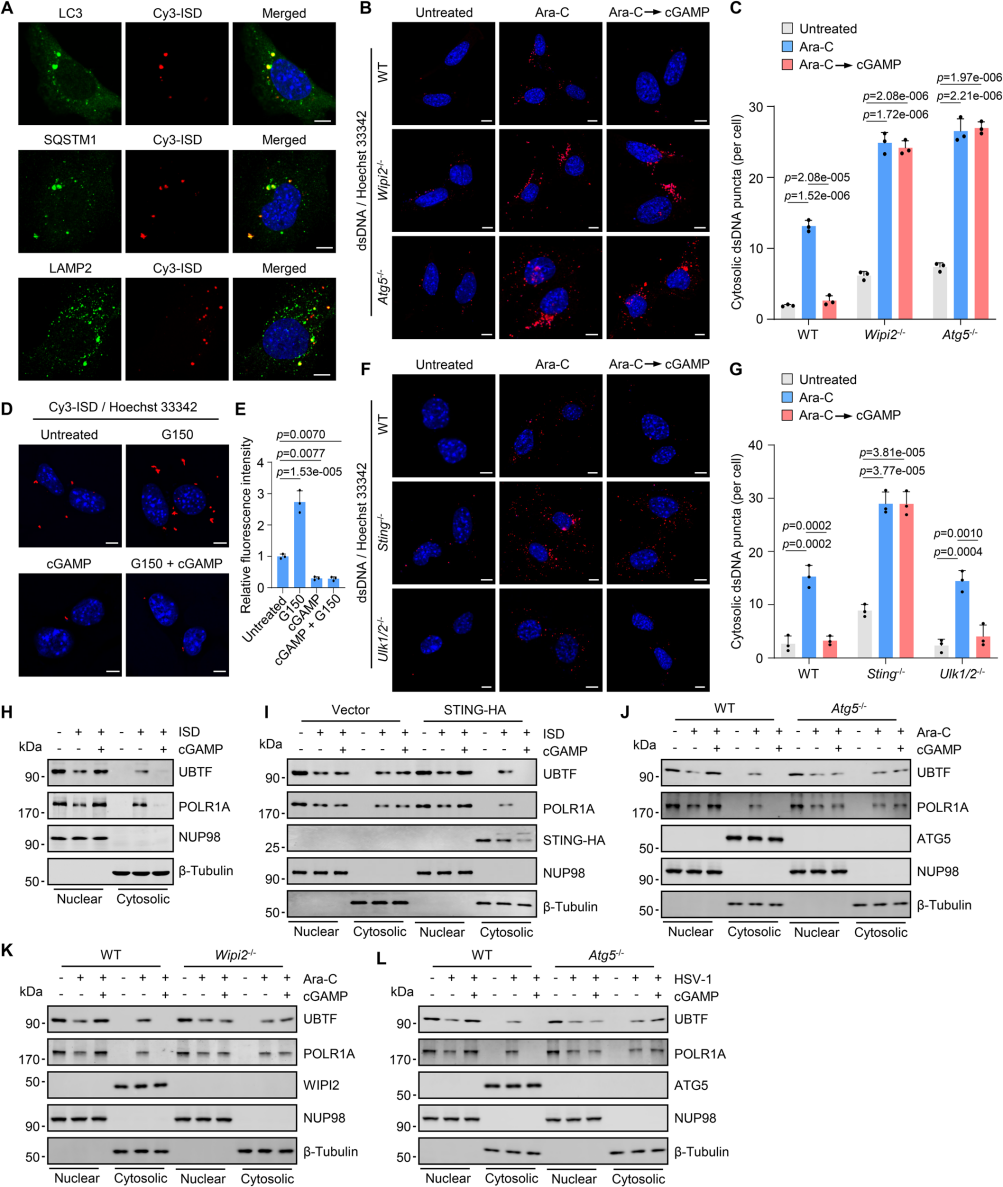
Cytosolic DNA-mediated retention of UBTF and POLR1A in the cytoplasm is abrogated by STING-induced autophagy. (**A**) Subcellular localization of Cy3 labeled ISD (Cy3-ISD) in MEFs. MEFs were transfected with Cy3-ISD and then stained with anti-LC3, anti-SQSTM1, and anti-LAMP2, respectively. (**B**) Endogenous cytosolic DNA in MEFs. Wild-type (WT), *Wipi2*^−/−^, and *Atg5*^−/−^ MEFs were treated with arabinofuranosyl cytidine (Ara-C) for 12 h and then cultured in fresh medium and incubated with or without cGAMP for another 12 h. After that, the cells were subjected to immunostaining using anti-dsDNA. (**C**) Statistical analysis of cytosolic DNA puncta in cells treated as in (**B**). 60 cells were analyzed from three independent experiments. (**D**) Exogenous Cy3-ISD in DLD1 cells. The cells were transfected with Cy3-ISD (4 μg/ml) for 6 h, followed by treatment with the cGAS inhibitor G150, the STING agonist cGAMP, or both of them for another 12 h. (**E**) Statistical analysis of the fluorescence intensity of Cy3-ISD in cells treated as in (**D**). (**F**) Endogenous cytosolic DNA in MEFs. WT, *Sting*^−/−^, and *Ulk1/2*^−/−^ MEFs were treated with Ara-C for 12 h and then cultured in fresh medium and incubated with or without cGAMP for another 12 h. (**G**) Statistical analysis of cytosolic DNA puncta in cells treated as in (**F**). 60 cells were analyzed from three independent experiments. (**H**–**K**) The protein levels of UBTF and POLR1A in the nuclear and cytoplasmic fractions of cells. MEFs and HEK293T cells reconstituted with or without STING were transfected with ISD (**H**, **I**), and WT, *Atg5*^−/−^, and *Wipi2*^−/−^ MEFs were treated with Ara-C for 12 h and then cultured in fresh medium (**J**, **K**). After that, all these cells were further treated with or without cGAMP for another 12 h. (**L**) The protein levels of UBTF and POLR1A in the nuclear and cytoplasmic fractions of MEFs. WT and *Atg5*^−/−^ MEFs were treated with or without cGAMP, and then infected with HSV-1 for 18 h. All statistical data are presented as mean ± SD of three independent experiments and analyzed by one-way ANOVA and Tukey’s post hoc test. Scale bars, 10 µm. Source data are available online for this figure.

Compelling evidence has shown that cytosolic DNA can be eliminated by the cGAS-STING pathway-induced autophagy (Gui et al, 2019; Wan et al, 2023; Watson et al, 2012; Xu et al, 2024). We chose to explore whether UBTF and POLR1A retention in the cytoplasm led by cytosolic DNA can be abrogated by STING-induced autophagy. We transfected cells with Cy3-ISD and found that Cy3-ISD in the cytoplasm can be targeted to punctuate structures positive for autophagosome marker LC3, autophagy receptor SQSTM1, and lysosomal protein LAMP2 (Fig. 3A), consistent with the previous observations (Xu et al, 2025). In addition, we checked endogenous cytosolic DNA in autophagy-deficient cells. As expected, endogenous DNA appeared in the cytoplasm of cells treated with Ara-C, and which was further accumulated in cells without WD-repeat domain phosphoinositide-interacting protein 2 (WIPI2) or autophagy-related 5 (ATG5) (Fig. 3B,C), two necessary components for STING-induced autophagy (Gui et al, 2019; Wan et al, 2023). Although both Ara-C treatment and ISD transfection could activate the cGAS-STING pathway, the STING agonist cGAMP activated STING more strongly, as indicated by *Ifnb1* mRNA levels (Fig. EV2B,C). Consistent with this observation, several previous studies have shown that cGAMP stimulation is required for the efficient clearance of cytosolic DNA via STING-induced autophagy (Gui et al, 2019; Wan et al, 2023; Xu et al, 2024). Therefore, to facilitate the clearance of cytosolic DNA, we employed cGAMP treatment to robustly activate STING-induced autophagy. As expected, when Ara-C-treated cells were further stimulated by cGAMP, cytosolic DNA was almost totally eliminated in WT rather than autophagy-deficient cells (Fig. 3B,C). Since STING-induced autophagy is triggered by the cGAS-STING signaling (Gui et al, 2019; Liu et al, 2019; Wan et al, 2023), we also examined its role in cytosolic DNA clearance. The clearance of Cy3-ISD was inhibited by the cGAS inhibitor G150 and promoted by the STING agonist cGAMP (Fig. 3D,E). Notably, G150 failed to inhibit cytosolic DNA clearance in cells treated with cGAMP (Fig. 3D,E), verifying that the autophagic clearance of cytosolic DNA depends specifically on downstream STING activation. In line with this, *Sting* knockout led to increased cytosolic DNA accumulation in Ara-C-treated cells, and abolished the clearance of cytosolic DNA triggered by further cGAMP treatment (Fig. 3F,G). In contrast, *Ulk1/2* knockout did not affect cytosolic DNA clearance in cGAMP-treated cells (Fig. 3F,G), confirming that ULK1/2 is dispensable for STING-induced autophagy (Gui et al, 2019). These data support the notion that STING-induced autophagy suffices to degrade cytosolic DNA (Gui et al, 2019; Wan et al, 2023; Xu et al, 2024).

**Figure EV3:**
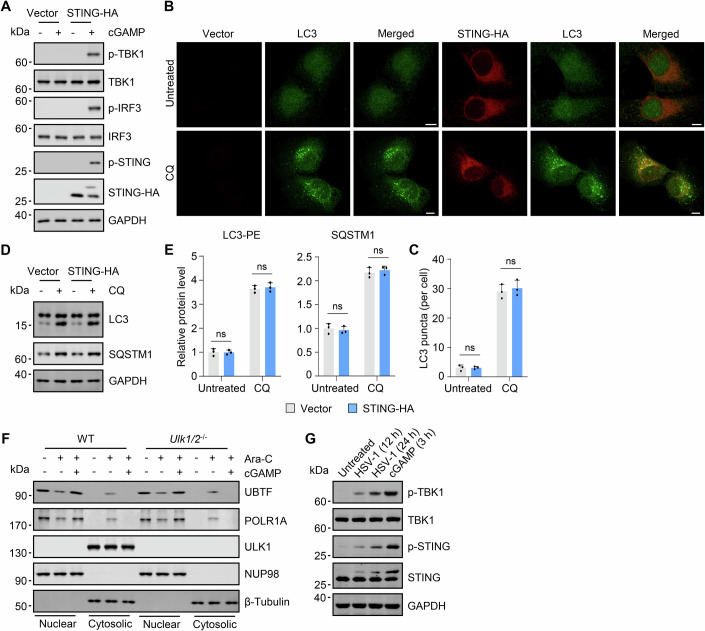
STING does not affect basal autophagy. (**A**) Verification of the functionality of STING in HEK293T cells reconstituted with HA-tagged STING. The cells were treated with or without cGAMP and subjected to western blot analysis using anti-phospho-TBK1 (Ser172), anti-phospho-IRF3 (Ser396), and anti-phospho-STING (Ser366). (**B**) LC3 punctum formation in HEK293T cells stably expressing STING-HA. The cells were treated with or without chloroquine (CQ), a lysosome inhibitor, and subjected to immunostaining using anti-LC3. Scale bars, 10 µm. (**C**) Statistical analysis of the number of LC3 puncta in cells treated as in (**B**). 60 cells were analyzed from three independent experiments. (**D**) The protein levels of lipidated LC3 and SQSTM1 in HEK293T cells stably expressing STING-HA. The cells were treated with or without CQ. (**E**) Statistical analysis of the protein levels of lipidated LC3 and SQSTM1 in cells treated as in (**D**). (**F**) The protein levels of UBTF and POLR1A in the nuclear and cytoplasmic fractions of cells. Wild-type (WT) and *Ulk1/2*^−/−^ MEFs were treated with Ara-C for 12 h and then stimulated with or without cGAMP for another 12 h. (**G**) Activation of STING by HSV-1 infection. MEFs were infected with HSV-1 or treated with cGAMP, then subjected to western blot analysis using anti-phospho-TBK1 (Ser172) and anti-phospho-STING (Ser366). All statistical data are presented as mean ± SD of three independent experiments and analyzed by the unpaired two-tailed Student’s *t* test. ns, not significant. Source data are available online for this figure.

We then performed cell fractionation experiments to investigate the effect of STING-induced autophagy on the subcellular distribution of UBTF and POLR1A. Obviously, the retention of UBTF and POLR1A in the cytoplasm induced by ISD transfection was abolished by cGAMP stimulation (Fig. 3H). Meanwhile, the reduction of UBTF and POLR1A in the nucleus was reversed (Fig. 3H). These data suggest that a strong activation of STING is required for the restoration of UBTF and POLR1A in the nucleus in cells with cytosolic DNA. To further validate the regulatory role of STING activation in this process, we utilized HEK293T cells, in which endogenous *STING* is silenced (Gui et al, 2019; Sui et al, 2017). We generated a HEK293T cell line stably expressing HA-tagged STING and confirmed that cGAMP treatment triggered the expected downstream signaling cascade (Fig. EV3A). It has been reported that STING controls energy stress-induced autophagy (Rong et al, 2022). By checking autophagosome formation and autophagic flux, we found that reconstitution of STING did not affect basal autophagy in HEK293T cells (Fig. EV3B–E). ISD transfection caused the appearance of UBTF and POLR1A in the cytoplasm in WT HEK293T cells and HEK293T cells reconstituted with STING (Fig. 3I). However, cGAMP treatment only abrogated the cytoplasmic distribution of these two proteins in cells expressing STING (Fig. 3I), confirming that a strong activation of STING is sufficient and necessary to abolish the retention of UBTF and POLR1A by cytosolic DNA. Ara-C treatment also induced the cytoplasmic distribution of UBTF and POLR1A in WT and *Atg5* knockout MEFs (Fig. 3J). Moreover, cGAMP treatment abolished the cytoplasmic distribution of these two proteins in WT but not *Atg5* knockout MEFs (Fig. 3J). A consistent result was also obtained when using *Wipi2* knockout MEFs (Fig. 3K). By contrast, in *Ulk1/2* knockout cells, the appearance of UBTF and POLR1A in the cytoplasm induced by Ara-C treatment was still abrogated by further stimulation of the cells with cGAMP (Fig. EV3F). Furthermore, we checked the effect of HSV-1 infection on the subcellular distribution of UBTF and POLR1A. In contrast to the strong activation by cGAMP, HSV-1 infection activated STING to a much weaker degree (Fig. EV3G). We found that cGAMP treatment can abolish HSV-1 infection-induced cytoplasmic distribution of UBTF and POLR1A in WT but not *Atg5* knockout MEFs (Fig. 3L). These results together suggest that STING-induced autophagy can abrogate the effect of cytosolic DNA on the subcellular distribution of UBTF and POLR1A.

**Figure EV4:**
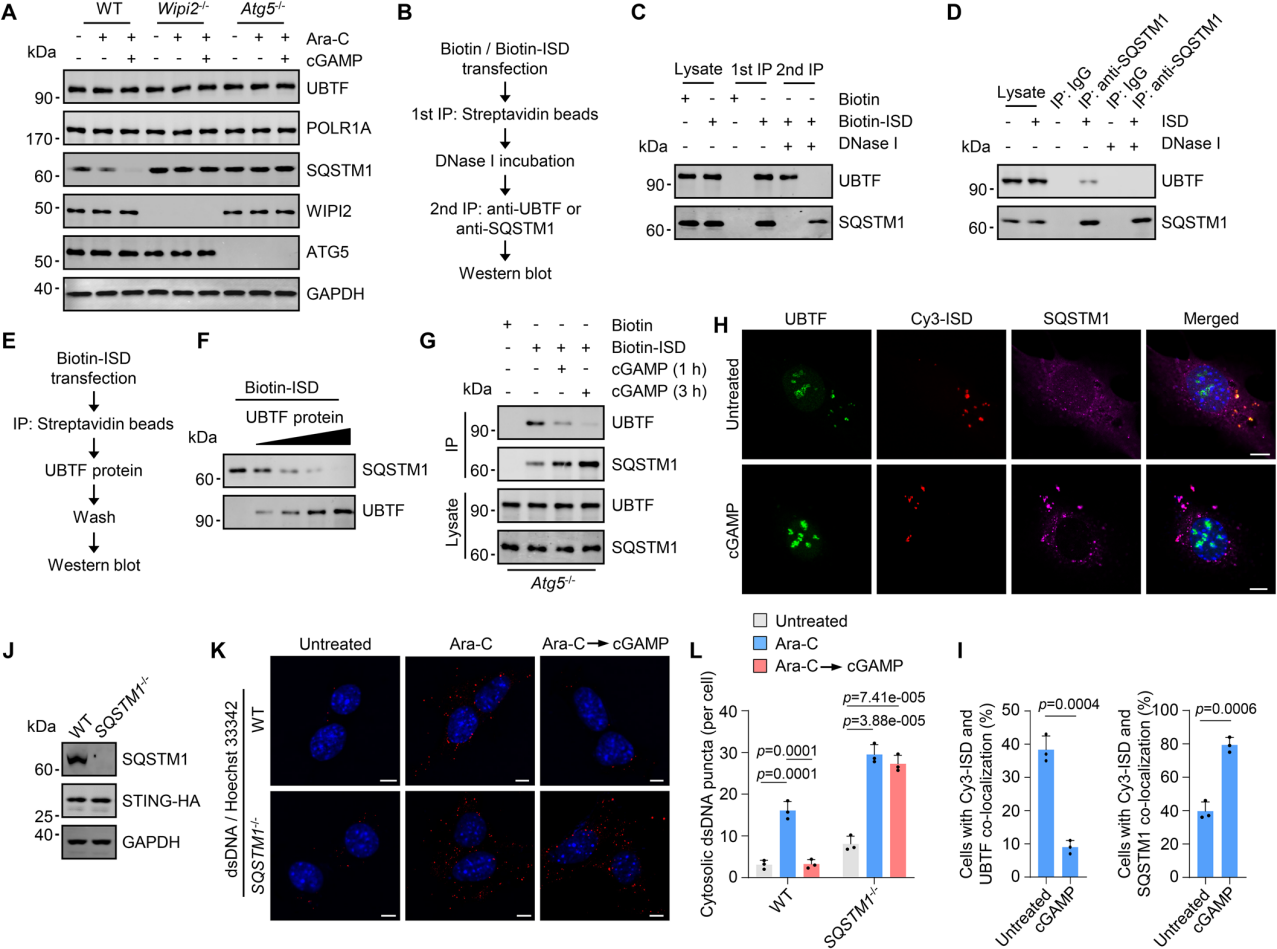
UBTF and SQSTM1 compete for binding to cytosolic DNA. (**A**) The protein levels of UBTF, POLR1A, and SQSTM1 in wild-type (WT), *Wipi2*^−/−^, and *Atg5*^−/−^ MEFs. The cells were treated with arabinofuranosyl cytidine (Ara-C) for 12 h and then cultured in fresh medium and incubated with or without cGAMP for another 12 h. The cell lysates from these cells were subjected to western blot analysis using anti-UBTF, anti-POLR1A, and anti-SQSTM1. (**B**) Schematic of the workflow for the two-step co-immunoprecipitation experiment. (**C**) MEFs transfected with biotin-ISD were lysed and incubated with streptavidin beads. After the incubation, the precipitates were treated with DNase I, the eluates were then subjected to the immunoprecipitation experiments with either anti-UBTF or anti-SQSTM1. The resulting immunoprecipitates were analyzed by western blot using anti-SQSTM1 and anti-UBTF, respectively. (**D**) MEFs transfected with ISD were lysed and incubated with or without DNase I. After the incubation, the cell lysates were subjected to the immunoprecipitation experiments using anti-SQSTM1. The resulting immunoprecipitates were analyzed by western blot using anti-UBTF. (**E**) Schematic of the workflow for examining the competitive binding of UBTF and SQSTM1 to biotin-ISD. (**F**) MEFs transfected with biotin-ISD were lysed and pulled down by streptavidin beads. After that, the precipitates were incubated with different amounts of recombinant UBTF protein, and then washed, and subjected to western blot analysis using anti-UBTF and anti-SQSTM1. (**G**) Association of endogenous UBTF and SQSTM1 with biotin-ISD in *Atg5*^−/−^ MEFs. The cells transfected with biotin-ISD were stimulated with cGAMP for 1 or 3 h. Biotin-ISD was then pulled down from these cells and the precipitates were subjected to western blot analysis using anti-UBTF and anti-SQSTM1. (**H**) Subcellular localization of UBTF and SQSTM1 in MEFs. The cells were transfected with Cy3-ISD and stimulated with or without cGAMP. After the treatments, the cells were stained using anti-UBTF and anti-SQSTM1. (**I**) Statistical analysis of the co-localization between Cy3-ISD and UBTF, as well as between Cy3-ISD and SQSTM1, in cells treated as in (**H**). (**J**) Verification of WT and *SQSTM1*^−/−^ HEK293 cells stably expressing HA-tagged STING. (**K**) Endogenous cytosolic DNA in cells. WT and *SQSTM1*^−/−^ cells stably expressing HA-tagged STING were treated with Ara-C for 12 h and then cultured in fresh medium and incubated with or without cGAMP for another 12 h. (**L**) Statistical analysis of cytosolic DNA puncta in cells treated as in (**K**). In total, 60 cells were analyzed from three independent experiments. All statistical data are presented as mean ± SD of three independent experiments. The unpaired two-tailed Student’s *t* test performed for (**I**), and one-way ANOVA and Tukey’s post hoc test performed for (**L**). Scale bars, 10 µm. Source data are available online for this figure.

Given that the reduction of UBTF and POLR1A in the nucleus is abolished when cytosolic DNA is eliminated, it indicates that UBTF and POLR1A in the cytoplasm may not be degraded by autophagy along with their binding partner cytosolic DNA. To validate this, we checked the protein levels of UBTF and POLR1A in WT and autophagy-deficient cells. The protein level of autophagy receptor SQSTM1 was decreased by Ara-C treatment, and which was further reduced by cGAMP stimulation (Fig. EV4A). Meanwhile, the protein levels of UBTF and POLR1A remained nearly unchanged (Fig. EV4A). Consistent with this, SQSTM1 but not UBTF or POLR1A was accumulated in cells with *Wipi2* or *Atg5* knockout (Fig. EV4A). Moreover, Ara-C treatment and cGAMP stimulation were unable to reduce the protein levels of all these proteins in autophagy-deficient cells (Fig. EV4A). Considering that both SQSTM1 and UBTF can interact with and target to cytosolic DNA, a simple hypothesis is that these two proteins may compete for binding to cytosolic DNA. At first, we conducted a two-step co-precipitation experiment to determine whether UBTF and SQSTM1 interact with each other for their targeting to cytosolic DNA (Fig. EV4B). Biotin-ISD was transfected into MEFs and pulled down by streptavidin beads, along with which both UBTF and SQSTM1 were co-precipitated (Fig. EV4C). After incubation of the precipitates with DNase I to eliminate the ISD, we immunoprecipitated UBTF and SQSTM1 from the precipitates and subjected the precipitates to western blot analysis using anti-SQSTM1 and anti-UBTF, respectively. The data clearly showed there is no co-immunoprecipitation between UBTF and SQSTM1 in the resulting precipitates (Fig. EV4C), ruling out the possibility that UBTF and SQSTM1 interact with each other on cytosolic DNA. Interestingly, when we immunoprecipitated SQSTM1 from the cells with ISD transfection, we detected a very low level of UBTF in the precipitate (Fig. EV4D). However, the appearance of UBTF in the precipitate was completely abolished when pretreating the cell lysate with DNase I (Fig. EV4D), confirming the essential role of cytosolic DNA in the recruitment of UBTF and SQSTM1. These results together suggest that UBTF and SQSTM1 target to cytosolic DNA independently of each other.

We then designed an assay to test whether UBTF and SQSTM1 indeed compete for binding to cytosolic DNA. We pulled down biotin-ISD from cells and incubated the precipitates with different amounts of recombinant UBTF protein. Following that, the precipitates were pulled down by streptavidin beads, washed intensively, and subjected to western blot analysis (Fig. EV4E). We found that more SQSTM1 protein was dissociated from the biotin-ISD when more UBTF protein was added to the precipitates (Fig. EV4F). Furthermore, we examined this competitive binding effect in cells transfected with biotin-ISD. We utilized *Atg5* knockout cells, in which autophagy-mediated degradation of cytosolic DNA and SQSTM1 was completely blocked. Clearly, treatment of these cells with cGAMP increased the binding of biotin-ISD to SQSTM1 while decreasing its association with UBTF (Fig. EV4G). In line with this, we observed that cGAMP treatment enhanced SQSTM1 targeting to Cy3-ISD but inhibited UBTF targeting (Fig. EV4H,I). Finally, we tested whether SQSTM1 indeed plays an important role in the clearance of cytosolic DNA. We utilized *SQSTM1* knockout cells and established a cell line stably expressing HA-tagged STING (Fig. EV4J). By using this cell line, we found that *SQSTM1* knockout markedly increased the accumulation of cytosolic DNA in Ara-C-treated cells (Fig. EV4K,L). Moreover, this accumulation of cytosolic DNA remained nearly unaffected by further treatment of these cells with cGAMP (Fig. EV4K,L).

Collectively, these data suggest a model in which STING-induced autophagy selectively degrades cytosolic DNA to abolish UBTF and POLR1A retention in the cytoplasm, thereby abrogating the reduction of these two proteins in the nucleus.

### Cytosolic DNA inhibits Pol I-mediated rDNA transcription

**Figure 4:**
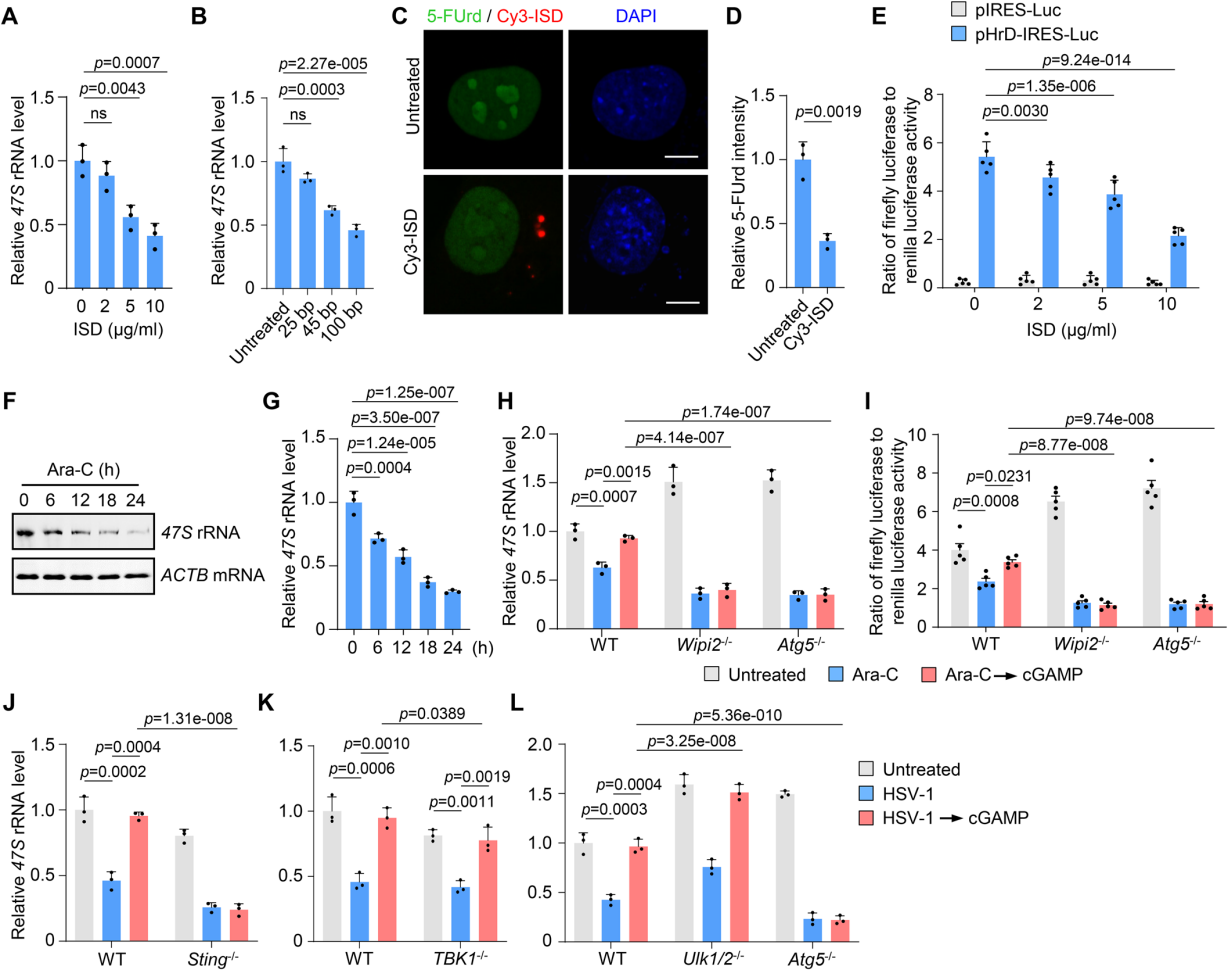
Cytosolic DNA inhibits Pol I-mediated rDNA transcription. (**A**, **B**) Cellular *47S* rRNA level in MEFs. MEFs were transfected with ISD with different concentrations (**A**), or DNA fragments with different lengths (**B**). *47S* rRNA level of the cells was measured by real-time PCR and normalized to *Actb* mRNA. (**C**) 5-fluorouridine (5-FUrd) incorporation in U2OS cells. U2OS cells transfected with Cy3 labeled ISD (Cy3-ISD) were incubated with 5-FUrd for 15 min and then fixed and stained using anti-BrdU. Scale bar: 10 µm. (**D**) Statistical analysis of the relative 5-FUrd intensity at the nucleolus in cells treated as in (**C**). (**E**) Ratio of firefly luciferase to renilla luciferase activity in U2OS cells. The cells were treated as in (**A**), and transfected with the indicated plasmids. After 24 h, the luciferase activity was measured. (**F**) Cellular *47S* rRNA level in U2OS cells. The cells were treated with arabinofuranosyl cytidine (Ara-C) for different time as indicated and the *47S* rRNA levels from these cells were monitored by northern blot. Blotted RNA was hybridized to the biotin-labeled probes comprising antisense sequences from human *47S* rRNA or human *ACTB* mRNA. (**G**) Statistical analysis of the *47S* rRNA levels in cells treated as in (**F**). The relative *47S* rRNA level was normalized to *ACTB* mRNA. (**H**, **I**) Cellular *47*S rRNA level (**H**), and rDNA promoter activity (**I**), in wild-type (WT), *Wipi2*^−/−^, and *Atg5*^−/−^ MEFs. The cells were treated with Ara-C for 12 h and then incubated with or without cGAMP for another 12 h. (**J**–**L**) Cellular *47*S rRNA level in WT and *Sting*^−/−^ MEFs (**J**), in WT and *TBK1*^−/−^ DLD1 cells (**K**), in WT, *Ulk1/2*^−/−^, and *Atg5*^−/−^ MEFs (**L**). The cells were treated with or without cGAMP, and then infected with HSV-1 for 18 h. All statistical data are presented as mean ± SD of three or five independent experiments. ns, not significant. One-way ANOVA and Tukey’s post hoc test performed for (**A**, **B**, **G**–**L**), the unpaired two-tailed Student’s *t* test performed for (**D**), and two-way ANOVA and Holm–Sidak post hoc test performed for (**E**, **H**–**L**). Source data are available online for this figure.

Considering that cytosolic DNA reduces the protein levels of UBTF and POLR1A in the nucleus, we determined to investigate whether cytosolic DNA affects the activity of rDNA transcription. At first, we measured the production of the precursor rRNA transcript *47S* rRNA in MEFs transfected with ISD. *47S* rRNA level in MEFs was decreased by ISD transfection through a dose-dependent manner (Fig. 4A). In line with the observation that longer DNA displays stronger binding affinity to UBTF and POLR1A, we found that longer DNA exerted stronger inhibitory effect on *47S* rRNA synthesis (Fig. 4B). As 5-fluorouridine (5-FUrd) is able to be incorporated into the nascent RNA, we then chose to check the incorporation of 5-FUrd at the nucleolus to directly assess rDNA transcription activity in situ (Torrano et al, 2006; Xu et al, 2022). Obviously, Cy3-ISD transfection significantly reduced 5-FUrd incorporation at the nucleolus (Fig. 4C,D). In addition, we examined the promoter activity of rDNA in cells. By using the human rDNA promoter luciferase reporter (Ghoshal et al, 2004; Xu et al, 2016), we found that ISD transfection decreased rDNA promoter activity (Fig. 4E). To further validate the role of cytosolic DNA in regulating rDNA transcription, we analyzed *47S* rRNA level by northern blot. The blotted RNA was hybridized to biotin-labeled probes comprising antisense sequences of human *47S* rRNA or human *ACTB* mRNA, and detected by biotin antibody. As expected, *47S* rRNA level in U2OS cells was decreased by Ara-C treatment through a time-dependent manner (Fig. 4F,G).

**Figure EV5:**
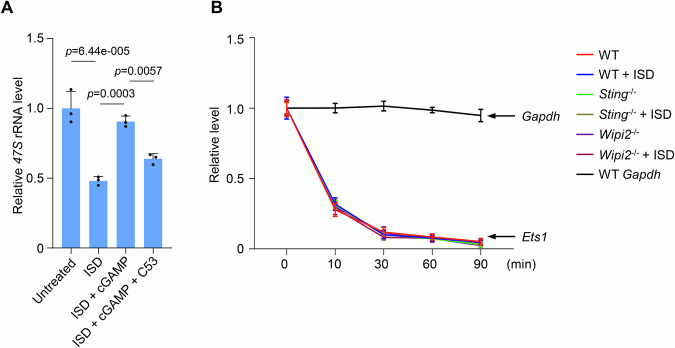
STING-induced autophagy does not affect *47S* rRNA degradation. (**A**) Cellular *47S* rRNA level in DLD1 cells. The cells were transfected with ISD and treated as indicated. *47S* rRNA level of the cells was measured by real-time PCR and normalized to *ACTB* mRNA. cGAMP, a STING agonist; compound 53 (C53), a STING agonist that can block its proton channel activity. (**B**) Cellular *Ets1* and *Gapdh* mRNA levels in wild-type (WT), *Sting*^−/−^, and *Wipi2*^−/−^ MEFs. The cells were transfected with or without ISD and incubated with 20 μg/ml actinomycin D (Act-D). The *Ets1* and *Gapdh* mRNA levels were measured by real-time PCR and normalized to *Actb* mRNA. *Gaphd* mRNA level was used as the internal control. Act-D, a transcription inhibitor. All statistical data are presented as mean ± SD of three independent experiments and analyzed by one-way ANOVA and Tukey’s post hoc test. Source data are available online for this figure.

We further checked the regulatory role of STING-induced autophagy in rDNA transcription. Supporting the previous observation (Xu et al, 2022), autophagy-deficient cells exhibited higher basal *47S* rRNA level (Fig. 4H). As expected, a reduction in *47S* rRNA level was also seen in all cells treated with Ara-C (Fig. 4H). However, the reduction was only reversed in WT rather than autophagy-deficient cells by further cGAMP stimulation (Fig. 4H). A consistent result was obtained when measuring the promoter activity of rDNA in these cells (Fig. 4I). Recently, induction of lysosome biogenesis has been reported to be a novel function of the cGAS-STING pathway (Huang et al, 2025; Lv et al, 2024a; Tang et al, 2025; Xu et al, 2025), and which is required for the efficient clearance of cytosolic DNA (Xu et al, 2025). Moreover, the proton channel activity is necessary for STING to stimulate lysosome biogenesis (Lv et al, 2024a; Xu et al, 2025), which prompts us to explore whether the proton channel activity of STING affects rDNA transcription. Compound 53 (C53) is a special STING agonist that can block STING’s proton channel activity (Liu et al, 2023; Xun et al, 2024). By using this chemical, we found that block of STING’s proton channel activity significantly decreased the recovery of *47S* rRNA level led by cGAMP treatment in cells with ISD transfection (Fig. EV5A). These data suggest that STING’s proton channel activity is required for the recovery of rDNA transcription activity in cells with cytosolic DNA. Given that autophagy is an intracellular degradation process, we also checked whether STING-induced autophagy regulates *47S* rRNA degradation. Treatment of the cells with actinomycin D (Act-D) in high concentration has been reported to inhibit rDNA transcription in less than 30 s (Lazdins et al, 1997; Popov et al, 2013). After the treatment, we measured the levels of *Ets1* (Lazdins et al, 1997; Popov et al, 2013), derived from *47S* rRNA, and *Gapdh* mRNA, used as the internal control. *Gapdh* mRNA level remained nearly unaffected at different time points (Fig. EV5B). By contrast, the half-life time of *Ets1* was less than 10 min (Fig. EV5B), which is consistent with the previous observations (Lazdins et al, 1997; Popov et al, 2013). However, the half-life time of *Ets1* remained nearly unchanged in *Sting* and *Wipi2* knockout cells. (Fig. EV5B). Moreover, ISD transfection had no effect on the half-life time of *Ets1* in all these cells (Fig. EV5B). These data suggest that STING-induced autophagy does not degrade *47S* rRNA. Finally, we investigated the role of viral infection in regulation of *47S* rRNA synthesis. As expected, HSV-1 infection decreased *47S* rRNA level, and which was restored by cGAMP treatment (Fig. 4J–L). However, knockout of *Sting* or *Atg5*, but not *TBK1* or *Ulk1/2*, abrogated the restoration of *47S* rRNA level in these cGAMP-treated cells (Fig. 4J–L).

Therefore, these data suggest that cytosolic DNA decreases rDNA promoter activity and inhibits rDNA transcription, and both of which can be abolished by STING-induced autophagy.

### Cytosolic DNA suppresses protein synthesis and curtails cell proliferation

**Figure 5:**
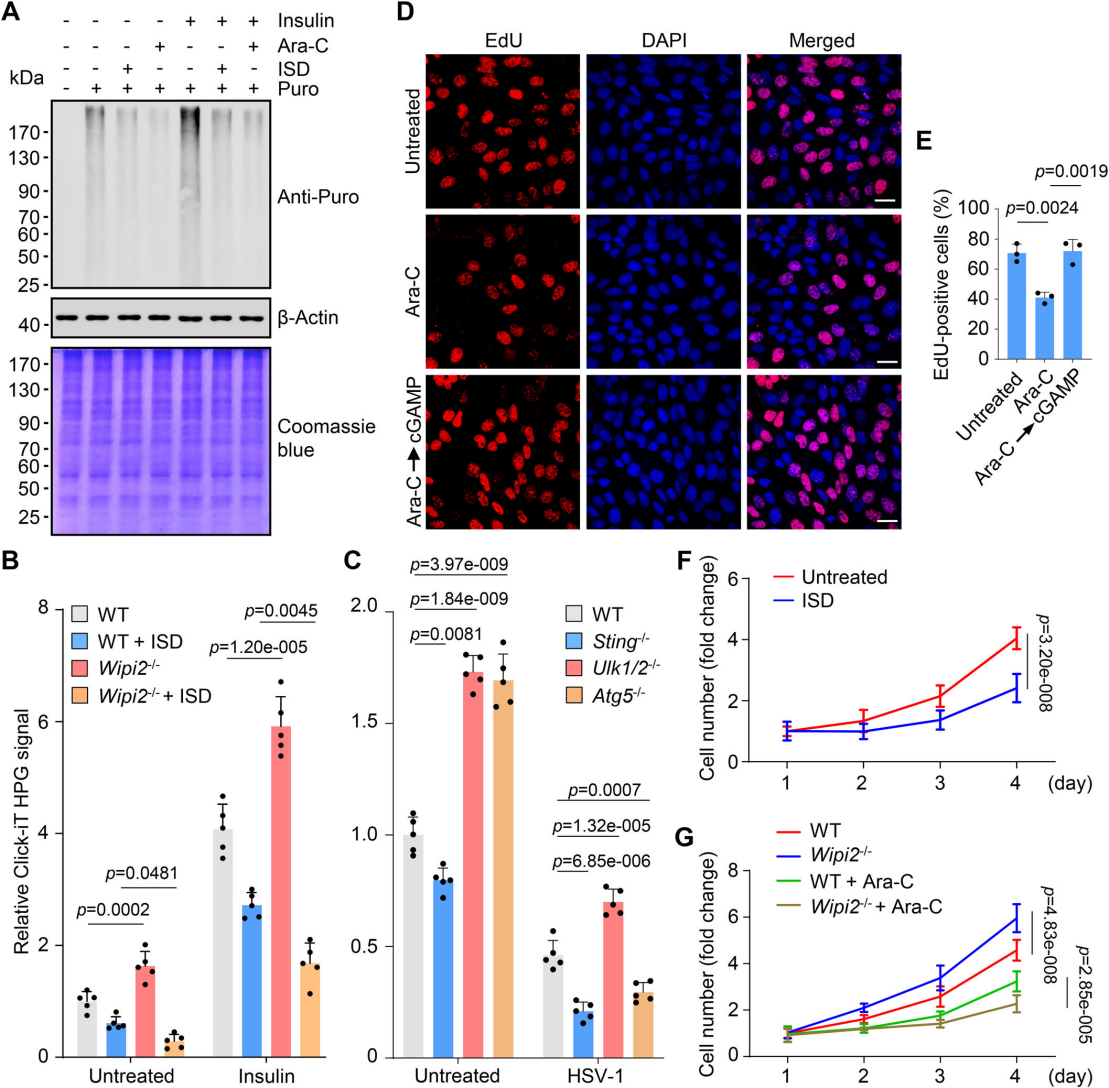
Cytosolic DNA suppresses protein synthesis and curtails cell proliferation. (**A**) Global protein synthesis of cells was detected using the surface sensing of translation (SUnSET) method. Cells were transfected with ISD for 24 h, or treated with arabinofuranosyl cytidine (Ara-C) for 12 h and cultured in Ara-C-free medium for another 12 h. Following that, in the presence or absence of puromycin, the cells were incubated with or without insulin for 30 min. The specificity of the anti-puromycin antibody was demonstrated by a sample without puromycin treatment. Coomassie blue staining was used as the loading control. (**B**, **C**) Global protein synthesis of cells was detected using a Click-iT HPG method. Wild-type (WT) and *Wipi2*^−/−^ MEFs transfected with or without ISD were incubated with or without insulin for 30 min (**B**), or WT, *Sting*^−/−^, *Ulk1/2*^−/−^, and *Atg5*^−/−^ MEFs were infected with or without HSV-1 for 18 h (**C**). (**D**) EdU incorporation in cells. Cells were treated with Ara-C for 12 h and then incubated with or without cGAMP for another 12 h. After that, all the cells were cultured in fresh medium for 24 h and then incubated with EdU for 2 h and stained with DAPI. Scale bar: 40 µm. (**E**) Statistical analysis of the EdU-positive cells treated as in (**D**). (**F**) Cell growth of MEFs with or without ISD transfection. Cell number was determined by counting for 4 days. (**G**) Cell growth of WT and *Wipi2*^−/−^ MEFs. Cells were treated with or without Ara-C for 12 h. After that, all the cells were cultured in fresh medium for 24 h and the cell number was then determined by counting for 4 days. All statistical data are presented as mean ± SD of three or five independent experiments. One-way ANOVA and Tukey’s post hoc test performed for (**B**, **C**, **E**), and two-way ANOVA and Holm–Sidak post hoc test performed for (**F**, **G**). Source data are available online for this figure.

The transcription of rDNA, a rate-limiting step of ribosome biogenesis, has been tightly regulated to control cellular protein synthesis and cell proliferation across species in distinct contexts (Arabi et al, 2005; Grandori et al, 2005; Grewal et al, 2005; Xu et al, 2016; Xu et al, 2022). To explore the physiological significance of cytosolic DNA induced-rDNA transcription inhibition in cells, we chose to examine cellular protein synthesis and cell proliferation. To check protein synthesis in cells, we utilized the surface sensing of translation (SUnSET) assay, detecting the puromycin incorporation into nascent proteins, to indicate the rate of nascent protein synthesis (Schmidt et al, 2009). Obviously, ISD transfection or Ara-C treatment decreased puromycin incorporation in cells (Fig. 5A). The incorporation of puromycin was further enhanced by insulin stimulation, but the promoting effect of insulin was impeded in cells with ISD transfection or Ara-C treatment (Fig. 5A). Considering that puromycin incorporation can interfere with protein synthesis to induce protein aggregates (Pankiv et al, 2007), we also examined cellular protein synthesis using a non-toxic method (Chorghade et al, 2017). Cells with *Wipi2* knockout displayed higher protein synthesis rate with or without insulin treatment (Fig. 5B), confirming the previous observation that autophagy deficiency upregulates basal rDNA transcription activity to enhance protein synthesis in cells (Xu et al, 2022). Intriguingly, *Wipi2* knockout cells exhibited lower protein synthesis rate than WT cells when all these cells were transfected with ISD (Fig. 5B). In addition, we measured protein synthesis rate in cells with viral infection. Clearly, knockout of *Sting* or *Atg5*, but not *Ulk1/2*, significantly downregulated protein synthesis rate in cells with HSV-1 infection (Fig. 5C). These data were consistent with the observations that abolishing STING-induced autophagy leads to much lower rDNA transcription activity in cells with Ara-C treatment, ISD transfection, or HSV-1 infection.

It is well known that highly proliferative cells demand higher DNA synthesis rate. We therefore checked cell proliferation by examining cellular DNA synthesis, which was indicated by EdU incorporation in cells. As expected, EdU incorporation was inhibited by Ara-C treatment, and the inhibition was abrogated by further cGAMP stimulation (Fig. 5D,E). In addition, we performed a time-course experiment to assess cell proliferation by directly counting cell number. Notably, ISD transfection significantly hindered the increase of cell number (Fig. 5F). Meanwhile, cell viability remained nearly unchanged (Appendix Fig. S2A), ruling out the involvement of cell death in the regulation of cell number. Finally, we investigated the regulatory role of STING-induced autophagy in cell proliferation. Consistent with the previous study (Xu et al, 2022), the number of *Wipi2* knockout cells increased faster than that of WT cells (Fig. 5G). However, when all these cells were treated with Ara-C, the increase in cell number of *Wipi2* knockout cells was lower than that of WT cells (Fig. 5G). Of note, all these cells exhibited similar cell viability with or without Ara-C treatment (Appendix Fig. S2B).

Taken together, these data suggest that cytosolic DNA inhibits cellular protein synthesis and cell proliferation, and these inhibitory effects can be abrogated by STING-induced autophagy, thereby further confirming the roles of cytosolic DNA and STING-induced autophagy in the regulation of rDNA transcription.

## Discussion

**Figure 6:**
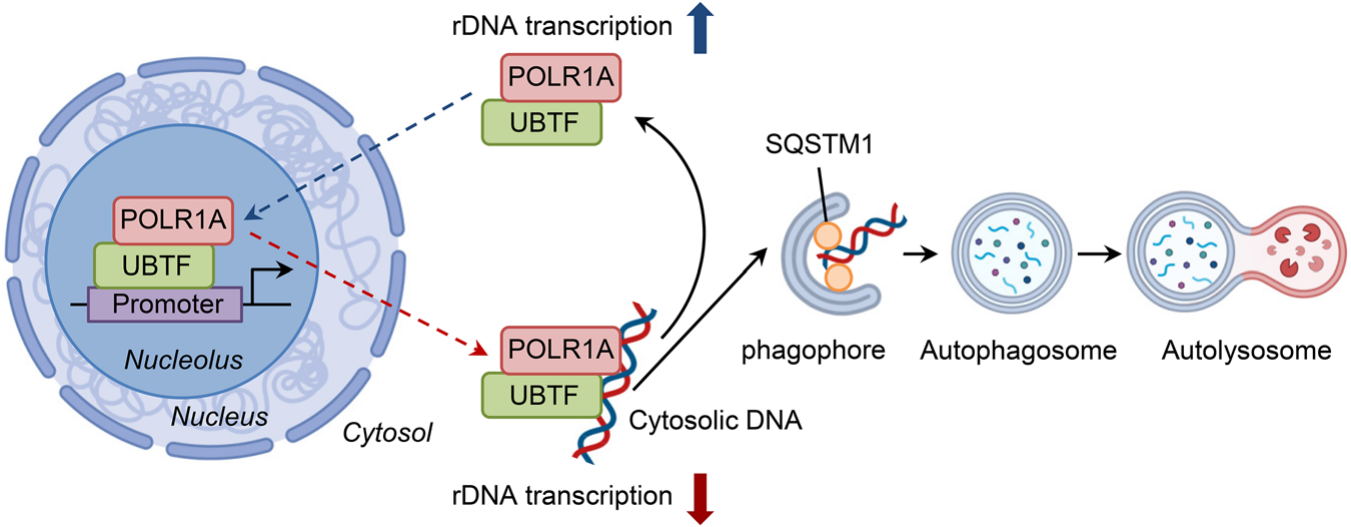
Schematic model for the roles of cytosolic DNA and STING-induced autophagy in the regulation of rDNA transcription. Cytosolic DNA sequesters UBTF and POLR1A in the cytoplasm and reduces these two proteins in the nucleus, leading to the inhibition of rDNA transcription of the cell. However, STING-induced autophagy can specifically degrade the cytosolic DNA to abolish UBTF and POLR1A retention in the cytoplasm, ultimately restoring rDNA transcription activity of the cell.

Cytosolic DNA, which originates from nuclear, mitochondrial, or pathogen genome upon cell damage or pathogen infection, has been identified as an important inducer of host innate immune responses by activating intracellular DNA-sensing machinery, such as the cGAS-STING pathway (Barber, 2011; Paludan and Bowie, 2013). In this study, we uncover a previously unappreciated function of cytosolic DNA in cell metabolism. By identifying UBTF and POLR1A, two essential components of the RNA Pol I transcription machinery, as novel binding partners of cytosolic DNA, we demonstrate that cytosolic DNA can function as an endogenous inhibitor of rDNA transcription, protein synthesis, and cell proliferation, and all these inhibitory effects can be totally abrogated by STING-induced autophagy (Fig. 6).

Our findings suggest that cytosolic DNA is involved in the regulation of innate immune response-independent biological processes, which broadens the physiological roles played by cytosolic DNA. Accumulation of nuclear or mitochondrial genome-derived DNA in the cytoplasm has been observed in diverse physiopathological contexts, such as aging, neurodegeneration, and cancer (Decout et al, 2021; Dong and Fitzgerald, 2024; Miller et al, 2021). Pathogen DNA can also appear in the cytoplasm of the host cells upon microbial infection (Gui et al, 2019; Watson et al, 2015; Watson et al, 2012; Wu et al, 2019). In addition to the induction of innate immune responses, cells have been demonstrated to intensively reprogram their catabolic and anabolic metabolism for survival and growth in these stress settings (Tattoli et al, 2012; Telser et al, 2019). Considering the key roles of rDNA transcription in metabolism, development, aging, and disease (Drygin et al, 2010; Sharifi and Bierhoff, 2018), cytosolic DNA-mediated rDNA transcription inhibition may represent a novel mechanism that contributes to the metabolic reprogramming of cells under some physiopathological conditions.

Consistent with previous studies (Watson et al, 2012; Xu et al, 2025), we show that autophagy receptor SQSTM1 can target to cytosolic DNA. Moreover, we find that SQSTM1 and UBTF competitively bind to cytosolic DNA, which explains how STING-induced autophagy efficiently degrades cytosolic DNA but not its binding partners UBTF and POLR1A. In addition, we find that several autophagy-deficient cells accumulate more cytosolic DNA with Ara-C treatment but are unable to retain more UBTF and POLR1A in the cytoplasm. Given that autophagy-deficient cells also accumulate much higher levels of SQSTM1 in the cytoplasm, the elevated SQSTM1 likely exerts a stronger inhibitory effect on Ara-C treatment-induced retention of UBTF and POLR1A in the cytoplasm. Therefore, the competitive binding to cytosolic DNA between SQSTM1 and UBTF may explain why differences in UBTF and POLR1A relocation between WT and autophagy-deficient cells under these conditions are relatively mild. It is worth noting that activation of STING stimulates the association of SQSTM1 with cytosolic DNA, which is thought to promote the targeting of cytosolic DNA, but not UBTF or POLR1A, to the autophagic membranes for subsequent degradation. Following the activation of the cGAS-STING signaling, TBK1 is activated and phosphorylates SQSTM1 to enhance the affinity of SQSTM1 to the ubiquitinated proteins (Matsumoto et al, 2015; Pilli et al, 2012; Prabakaran et al, 2018). Interestingly, cytosolic DNA can also be marked by ubiquitin, and which seems to act as the key signal for SQSTM1 recruitment (Watson et al, 2012). Therefore, TBK1-mediated phosphorylation of SQSTM1 may also regulate SQSTM1 binding to cytosolic DNA. However, activation of STING rather than TBK1 has been demonstrated to control the autophagic clearance of cytosolic DNA (Gui et al, 2019). Given that activation of TBK1 is only one of the downstream events of the cGAS-STING pathway (Zhang et al, 2025), it is also possible that the cGAS-STING pathway regulates SQSTM1 binding to cytosolic DNA through a TBK1-independent manner. Of note, both UBTF and POLR1A show high affinity for rDNA at the nucleolus (Xu et al, 2016), whether UBTF and POLR1A utilize the same mechanism to interact with cytosolic DNA remains unknown and warrants further investigation.

DNA binding in the cytoplasm is required for the activation of the enzyme cGAS and the subsequent initiation of the cGAS-STING signaling, it would be interesting to explore whether the association of cytosolic DNA with UBTF and POLR1A interferes with its affinity to cGAS in the cytoplasm and then affects the intensity of the cGAS-STING signaling. In addition, cGAS has been demonstrated to be highly abundant in the nucleus, where it can play several roles in immune response-independent processes, such as DNA damage response and epigenetic reprogramming (Liu et al, 2018; Lv et al, 2024b). Of note, cGAS still seems to rely on its DNA binding capacity to exert these functions. Therefore, it would be interesting to explore whether cGAS in the nucleus can directly bind to rDNA to regulate rDNA transcription in some physiopathological contexts.

## Methods

**Table:** Reagents and tools table

Reagent/resource	Reference or source	Identifier or catalog number
**Experimental models**		
HEK293T cells	ATCC	CRL-3216
U2OS cells	Dr. Qiming Sun	N/A
HEK293 cells	You et al, 2019	N/A
*SQSTM1*^−/−^ HEK293 cells	You et al, 2019	N/A
DLD1 cells	Dr. Pinglong Xu	N/A
*TBK1*^−/−^ DLD1 cells	Dr. Pinglong Xu	N/A
MEFs	Wan et al, 2018	N/A
*Atg5*^−/−^ MEFs	Wan et al, 2018	N/A
*Wipi2*^−/−^ MEFs	Dr. Hong Zhang	N/A
*Sting*^−/−^ MEFs	Dr. Lei Liu	N/A
*Ulk1/2*^−/−^ MEFs	Dr. Quan Chen	N/A
**Recombinant DNA**		
pCDNA3.1-STING-HA	Dr. Quan Chen	N/A
pGEX-4T-1-GST-UBTF	This study	N/A
pGEX-4T-1-GST-POLR1A	This study	N/A
pGEX-4T-1-GST-TUBB	This study	N/A
**Antibodies**		
Anti-biotin	Abcam	ab53494
Anti-dsDNA	Abcam	ab27156
Anti-WIPI2	Abcam	ab105459
Anti-LAMP2	Abcam	ab13524
Anti-NUP98	Cell Signaling Technology	2598S
Anti-TBK1/NAK	Cell Signaling Technology	3504S
Anti-phospho-TBK1/NAK (Ser172)	Cell Signaling Technology	5483S
Anti-IRF3	Cell Signaling Technology	4302S
Anti-phospho-IRF3 (Ser396)	Cell Signaling Technology	4947S
Anti-STING	Cell Signaling Technology	50494S
Anti-phospho-STING (Ser366)	Cell Signaling Technology	19781S
Anti-LC3	MBL	PM036
Anti-HA	MBL	M180-3
Anti-ATG5	Novus Biologicals	NB110-53818
Anti-UBTF	Novus Biologicals	NBP1-82545
Anti-RPA194/POLR1A	Novus Biologicals	NBP2-56122
Anti-p62/SQSTM1	Proteintech	18420-1-AP
Anti-GAPDH	Proteintech	10494-1-AP
Anti-BrdU	Sigma-Aldrich	B8434
Anti-β-Tubulin	Sigma-Aldrich	T5293
Anti-β-Actin	Sigma-Aldrich	A5316
Anti-puromycin	Sigma-Aldrich	MABE343
Anti-Atg1/ULK1	Sigma-Aldrich	A7481
Anti-UBTF	Santa Cruz Biotechnology	sc-13125
Anti-RPA194/POLR1A	Santa Cruz Biotechnology	sc-48385
Anti-TIF-IA/RRN3	Santa Cruz Biotechnology	sc-390464
Goat anti-mouse IgG (H + L), Alexa Fluor 488	Thermo Fisher Scientific	A-11001
Donkey anti-rabbit IgG (H + L), Alexa Fluor 488	Thermo Fisher Scientific	A-21206
Goat anti-rat IgG (H + L), Alexa Fluor 488	Thermo Fisher Scientific	A-11006
Donkey anti-mouse IgG (H + L), Alexa Fluor 546	Thermo Fisher Scientific	A10036
Donkey anti-rabbit IgG (H + L), Alexa Fluor 546	Thermo Fisher Scientific	A10040
Goat anti-rabbit IgG (H + L), Alexa Fluor 635	Thermo Fisher Scientific	A-31576
Donkey anti-rabbit IRDye800CW	LI-COR Biosciences	926-32213
Donkey anti-mouse IRDye680	LI-COR Biosciences	926-32222
**Oligonucleotides and other sequence-based reagents**		
Please see Appendix Table S1 for the list of synthesized DNA fragments	This study	N/A
Please see Appendix Table S2 for the list of siRNAs	This study	N/A
Please see Appendix Table S3 for the list of primers used for real time PCR	This study	N/A
Please see Appendix Table S4 for the list of biotin-labeled antisense sequences	This study	N/A
**Chemicals, enzymes and other reagents**		
cGAMP	Invivogen	tlrl-nacga23-1
Puromycin	Invivogen	ant-pr-1
G418	Invivogen	ant-gn-1
Chloroquine (CQ)	Sigma-Aldrich	C6628
Saponin	Sigma-Aldrich	S7900
Actinomycin D (Act-D)	Sigma-Aldrich	A1410
5-fluorouridine (5-FUrd)	Sigma-Aldrich	F5130
Lipofectamine 3000	Thermo Fisher Scientific	L3000015
DAPI	Thermo Fisher Scientific	D8417
TRIzol	Thermo Fisher Scientific	15596018
DNase I	Thermo Fisher Scientific	EN0521
Fetal bovine serum (FBS)	Gibco	10091148
Opti-MEM	Gibco	31985-062
Arabinofuranosyl cytidine (Ara-C)	Selleckchem	S1648
Cycloheximide (CHX)	Selleckchem	S7418
Leptomycin B (LMB)	Selleckchem	S7580
Compound 53 (C53)	MedChemExpress	HY-147010
G150	MedChemExpress	HY-128583
Hoechst 33342	Beyotime	C1022
Insulin	Beyotime	P3376
Streptavidin Magnetic Beads	Beyotime	P2151
M-MLV reverse transcriptase	Promega	M1701
TB Green Premix Ex Taq II (Tli RNaseH Plus)	Takara	RR820A
**Software**		
GraphPad Prism 8.0.1	GraphPad Software	https://www.graphpad.com/
Odyssey infrared imaging system	LI-COR Biosciences	N/A
CFX96 Touch Real-Time Detection System	Bio-Rad Laboratories	N/A
ImageJ	NIH	https://imagej.nih.gov/ij/
Zeiss Zen software	Zeiss	https://www.zeiss.com.cn/
BioRender	BioRender	https://www.biorender.com/
**Other**		
Zeiss LSM 880 microscope	Zeiss	Zeiss

### Cell culture and transfection

U2OS, DLD1, HEK293, HEK293T cells and MEFs were grown in Dulbecco’s modified Eagle’s medium supplemented with 10% FBS at 37 °C under an atmosphere of 5% CO_2_. Lipofectamine 3000 was used for the transfection of plasmids, synthesized DNA fragments, or siRNA duplexes according to the manufacturer’s instructions. For RNA interference, the transfection was repeated twice with an interval of 24 h to achieve the maximal RNAi efficacy. HEK293T cells stably expressing STING-HA were generated in a previous study (Wan et al, 2023). Wild-type (WT) and *SQSTM1* knockout HEK293 cells stably expressing STING-HA were generated by transient transfection followed by selection with G418. Cell lines were authenticated by STR profiling and routinely tested for mycoplasma contamination. The sequences of synthesized oligonucleotides used as exogenous cytosolic DNA are listed in Appendix Table S1. The sequences of siRNA duplexes used are listed in Appendix Table S2.

### Reagents and treatments

The chemicals were used as follows unless indicated otherwise: cGAMP, 500 nM; arabinofuranosyl cytidine (Ara-C), 5 μM; compound 53 (C53), 10 μM; G150, 5 μM; cycloheximide (CHX), 5 μM; leptomycin B (LMB), 40 nM; 5-fluorouridine (5-FUrd), 2 mM; insulin, 500 nM; puromycin, 1 µM. cGAMP was delivered into cells by permeabilization with digitonin (10 μg/ml) for 15 min in buffer A (50 mM HEPES-KOH, pH 7.2, 100 mM KCl, 3 mM MgCl2, 0.1 mM DTT, 85 mM sucrose, 0.2% BSA, 1 mM ATP) (Gui et al, 2019).

### Confocal microscopy

For immunostaining, cells were fixed with 4% formaldehyde followed by permeabilization and blocking with PBS containing 10% FCS and 0.1% saponin. Then the cells were incubated with appropriate primary and secondary antibodies in 0.1% saponin as indicated. For immunostaining of endogenous cytosolic DNA, we used a low-permeabilization buffer (0.1% Tween 20, 0.01% Triton-X in PBS) that allows the antibody to pass through plasma membrane, but not mitochondrial membrane or nuclear envelope (Spada et al, 2019). Nuclei were stained with DAPI or Hoechst 33342. Confocal images were captured on a laser scanning confocal microscope LSM880 (Carl Zeiss) and analyzed with the LSM 880 software (Carl Zeiss).

### Protein expression and purification

GST-tagged UBTF, POLR1A, and β-Tubulin were expressed in Escherichia coli BL21 by induction with 0.1 mM isopropyl β-D-thiogalactopyranoside for 12 h at 28 °C. The recombinant proteins were purified using glutathione-sepharose 4B beads (GE Healthcare Life Sciences, 17-0756-01) and incubated with thrombin at 4 °C for 6 h to release the proteins from the GST.

### Western blot and immunoprecipitation

Western blot was performed as described previously (Wan et al, 2017). In brief, samples were denatured and loaded on sodium dodecyl sulfate polyacrylamide gels. Then, they were transferred to polyvinylidene difluoride membranes. After blocking with 5% (w/v) BSA, the membranes were stained with the corresponding primary and secondary antibodies. The specific bands were analyzed using an Odyssey Infrared Imaging System. For immunoprecipitation experiments, the cell lysates were mixed with anti-UBTF or anti-SQSTM1 at 4 °C overnight, followed by the addition of protein A/G agarose beads at 4 °C for 4 h. After that, the immunocomplexes were washed extensively four times and resolved in SDS sample buffer. The samples were subjected to western blot analysis.

### Biotin-based pull-down assay

Cells were transfected with biotin-labeled ISD (biotin-ISD) and then treated as indicated. Following that, the cells were treated with or without DNase I and incubated with streptavidin beads for 4 h at 4 °C. The beads were then washed extensively four times and resuspended in SDS sample buffer. The resulting samples were subjected to western blot analysis.

### Cell fractionation

Nuclear and cytoplasmic fractions were isolated as described previously (Xu et al, 2025). Cells were washed with iced PBS and scraped into hypotonic buffer (10 mM HEPES, pH 8.0, 10 mM KCl, 3 mM MgCl_2_, 0.5 mM DTT, and protease inhibitors), and then incubated on ice for 10 min and Triton X-100 was added. After centrifugation at 500× *g* for 5 min at 4 °C, the supernatant was used as the cytoplasmic fraction. The pellet was washed twice with hypotonic buffer and reconstituted in RIPA buffer (100 mM TRIS-HCl, pH 8.0, 1% Triton X-100, 100 mM NaCl, 0.5 mM EDTA, and protease inhibitors). After centrifugation at 15,000× *g* for 10 min at 4 °C, the resulting supernatant was used as the nuclear fraction.

### RNA isolation and real-time PCR

Total RNA was isolated using TRIzol from cells and reverse transcribed using random hexamers, dNTPs, and M-MLV reverse transcriptase. The resulting cDNA was subjected to real-time PCR analysis with gene-specific primers. The sequences of the primers used are listed in Appendix Table S3.

### Northern blot

Northern blot was carried out as described previously (Xu et al, 2022). Total RNA isolated from cells was separated on denaturing agarose gel and transferred to nylon membrane (Millipore, INYC00010). Blotted RNA was then crosslinked and hybridized to biotin-labeled probes comprising antisense sequences of human *47S* rRNA and human *ACTB* mRNA, and detected by biotin antibody. The sequences of the probes used are listed in Appendix Table S4.

### Luciferase reporter assay

Cells were transfected with the indicated amount of ISD for 12 h, or treated with Ara-C for 12 h and then incubated with or without cGAMP for another 12 h. After that, pIRES-Luc or pHrD-IRES-Luc (expressing firefly luciferase) along with the internal control pRLTK (expressing renilla luciferase) were co-transfected into cells. The cells were lysed in passive lysis buffer 24 h after transfection, and assayed for luciferase activity using a luciferase assay kit (Promega, E1910) according to the manufacturer’s instructions.

### Protein synthesis and cell proliferation assays

Puromycin incorporation assay was carried out as described previously (Xu et al, 2020; Xu et al, 2022). Briefly, cells were treated with or without insulin in the presence of puromycin for 30 min and followed by extraction with 0.015% digitonin supplemented permeabilization buffer (50 mM TRIS-HCl, pH 7.5, 5 mM MgCl_2_, 25 mM KCl) containing protease inhibitors to release free puromycin. The cell lysates were subjected to western blot analysis, and the nascent proteins were detected using anti-puromycin antibody. For Click-iT HPG Alexa Fluor 488 protein synthesis assay, the protein synthesis rate was assessed using a kit (Thermo Fisher Scientific, C10428) according to the manufacturer’s instructions. For cell proliferation assay, the cells were counted using a cell counting kit (Bimake, B34302). The EdU incorporation assay was carried out using a kit (Sangon Biotech, E607204).

### Viral infection

HSV-1 was kindly provided by Dr. Pinglong Xu (Zhejiang University, China). For viral infection assay, the cells were treated with or without cGAMP, and then infected with HSV-1 for the indicated time.

### Cell viability assay

Cell viability assay was performed as described previously (Xu et al, 2020). Apoptotic cells were examined by the detection of Annexin V and propidium iodide (PI) double-positive cells. After specific treatments, cells were collected by trypsinization, centrifuged, washed with PBS, and stained with FITC-conjugated Annexin V and PI (BD Biosciences) according to the manufacturer’s instructions. Following that, the cells were subjected to flow cytometry analysis using CytoFLEX LX flow cytometer (Beckman Coulter).

### HPLC-MS/MS

Mass spectrometric analysis was carried out as described previously with some modifications (Wan et al, 2018). To characterize the interacting proteins of cytosolic DNA, MEFs transiently expressing biotin-ISD were purified with streptavidin beads. The streptavidin beads were washed extensively 4 times and dissolved with 40 μl urea (8 M)/DTT (10 mM). The mixture was sonicated for 30 min at room temperature, and then sequentially treated with IAA (10 mM) and trypsin to alkylate the resulting thiol group and digest the proteins for 16 h at 37 °C at an enzyme-to-substrate ratio of 1:50 (w/w). For LC-MS/MS analysis, the tryptic digested peptides were directly loaded onto an in-house packed capillary reverse-phase C18 column (150 mm length, 360 μm OD × 75 μm ID, 2.5 µm particle, 100 Å pore diameter) connected to an Agilent HPLC1260 system (Agilent Technology) and then desalted online for 60 min. The samples were analyzed with a 180 min-HPLC gradient from 0% to 100% of 0.1% formic acid in acetonitrile at a flow rate of 300 nl/min. The eluted peptides were ionized and directly introduced into a Q-Exactive mass spectrometer (Thermo Fisher Scientific) using a nano-spray source. Survey full-scan MS spectra (*m/z* 300–1800) were acquired in the Orbitrap analyzer with resolution *r* = 70,000 at *m/z* 400.

### Quantification and statistical analysis

All the statistical data from three or five independent repeats are shown for every figure, except where specified otherwise in the figure legends. All statistical analyses were performed using GraphPad Prism 8.0.1 software. The data were presented as mean ± SD, and statistical significance was determined by the unpaired two-tailed Student’s *t* test, or the ANOVA test followed by Tukey’s post hoc test or Holm–Sidak post hoc test as indicated in the figure legends.

## Supplementary information

Appendix

Peer Review File

Source data Fig. 1

Source data Fig. 2

Source data Fig. 3

Source data Fig. 4

Source data Fig. 5

Expanded View and Appendix Source Data

Expanded View Figures

## Data Availability

This study includes no data deposited in external repositories. The source data of this paper are collected in the following database record: biostudies:S-SCDT-10_1038-S44318-026-00792-2.
