# Supplementary material for: Cytosolic DNA inhibits rDNA transcription by retaining the RNA polymerase I transcription machinery
Source: EMBO J. 2026 May 5;45(12):4153–75. doi: 10.1038/s44318-026-00792-2 (PMC13270134; doi:10.1038/s44318-026-00792-2)
Supplement: Supplementary file 1 — Appendix [file 44318_2026_792_MOESM1_ESM.pdf]

Cytosolic DNA inhibits rDNA transcription by retaining the RNA polymerase I transcription machinery

Yinfeng Xu, Qian Wang, Chuying Qian, Sheng Lu, Zhengfu He, Wei Liu, and Wei Wan

Appendix

Table of Contents

Appendix Figure S1 Page 2

Appendix Figure S2 Page 3

Appendix Table S1 Page 4

Appendix Table S2 Page 5

Appendix Table S3 Page 6

Appendix Table S4 Page 7

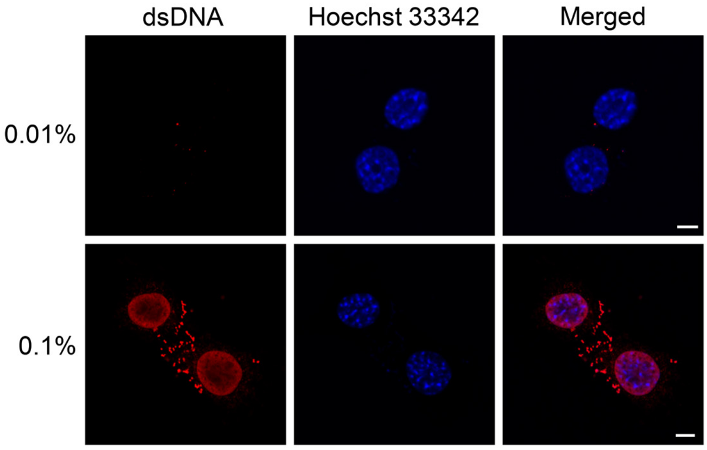

Appendix Figure S1. Verification of the immunostaining of cytosolic DNA.

MEFs were immunostaining by anti-dsDNA in a buffer with low-permeabilization (0.01% Triton X-100) or high-permeabilization (0.1% Triton X-100). Scale bars, 10 µm. Source data are available online for this figure.

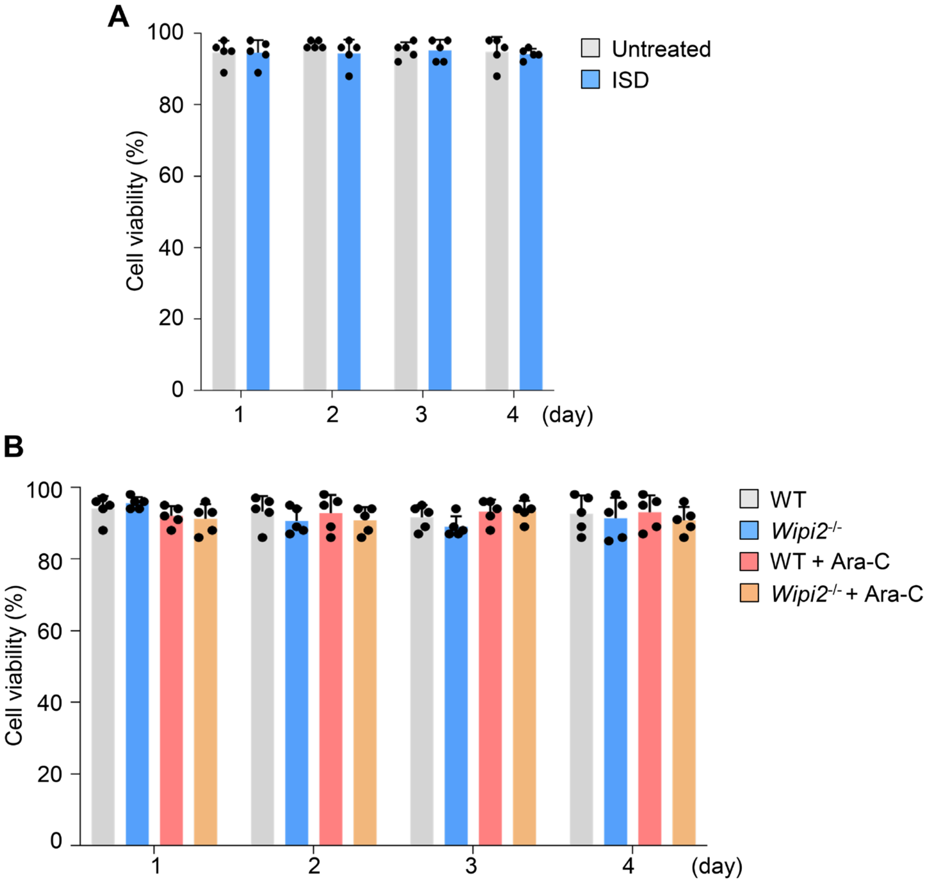

Appendix Figure S2. Cytosolic DNA does not affect cell viability.

(**A**, **B**) Wild-type (WT) MEFs were transfected with or without ISD (**A**), or WT and *Wipi2*$^{-/-}$ MEFs were treated with or without arabinofuranosyl cytidine (Ara-C) for 12 h (**B**). After that, all the cells were cultured in fresh medium for 24 h and the apoptotic cells were then indicated by the detection of Annexin V and propidium iodide (PI) double-positive cells for four days. All statistical data are presented as mean ± SD of five independent experiments. Source data are available online for this figure.

Appendix Table S1. Oligonucleotides used as cytosolic DNA

Length	Type	Sequence 5'-3'
25 bp	sense	AAAACAAACAACACAACAAACAAAA
	anti-sense	TTTTGTTTGTTGTGTTGTTTGTTTT
45 bp (ISD)	sense	TACAGATCTACTAGTGATCTATGACTGA TCTGTACATGATCTACA
	anti-sense	TGTAGATCATGTACAGATCAGTCATAG ATCACTAGTAGATCTGTA
45 bp (AT)	sense	ATATATATATATATATATATATATATATA TATATATATATATA
	anti-sense	TATATATATATATATATATATATATATAT ATATATATATATAT
45 bp (CG)	sense	CGCGCGCGCGCGCGCGCGCGCGCGCG CGCGCGCGCGCGCGCGC
	anti-sense	GCGCGCGCGCGCGCGCGCGCGCGCGC GCGCGCGCGCGCGCGCG
100 bp	sense	ACATCTAGTACATGTCTAGTCAGTATCT AGTGATTATCTAGACATACATCTAGTAC ATGTCTAGTCAGTATCTAGTGATTATCT AGACATGGACTCATCC
	anti-sense	GGATGAGTCCATGTCTAGATAATCACT AGATACTGACTAGACATGTACTAGATG TATGTCTAGATAATCACTAGATACTGAC TAGACATGTACTAGATGT

Appendix Table S2. siRNA duplex sequences

Name	Type	Sequence 5'-3'
siNC	sense	UUCUCCGAACGUGUCACGUTT
	anti-sense	ACGUGACACGUUCGGAGAATT
si*UBTF*-1	sense	GAAGUUCCGUACAUUGACATT
	anti-sense	UGUCAAUGUACGGAACUUCTT
si*UBTF*-2	sense	GGAGAAACUGAUGUGGAUUTT
	anti-sense	AAUCCACAUCAGUUUCUCCTT
si*POLR1A*-1	sense	CAACUACGAGGUGAUAAUGAATT
	anti-sense	UUCAUUAUCACCUCGUAGUUGTT
si*POLR1A*-2	sense	GCAAACGGCCUGUACGAUUTT
	anti-sense	AAUCGUACAGGCCGUUUGCTT

Appendix Table S3. Primers used for real-time PCR

Name	Type	Sequence 5'-3'
47S rRNA (mouse)	forward	TTTTGGGGAGGTGGAGAGTC
	reverse	AGAGAACTCCGGAGCACCAC
Actb (mouse)	forward	CGTGCACCGCAAGTGCTT
	reverse	GCTGTCGCCTTCACCGTT
Ets1 (mouse)	forward	TTTTGGGGAGGTGGAGAGTC
	reverse	AGAGAACTCCGGAGCACCAC
Gapdh (mouse)	forward	ACTCCACTCACGGCAAATTC
	reverse	TCTCCATGGTGGTGAAGACA
Ifnb1 (mouse)	forward	AGGGCGGACTTCAAGATC
	reverse	CTCATTCCACCCAGTGCT
47S rRNA (human)	forward	TGTCAGGCGTTCTCGTCTC
	reverse	GAGAGCACGACGTCACCAC
ACTB (human)	forward	TTGCGTTACACCCTTTCTTG
	reverse	CACCTTCACCGTTCCAGTTT

Appendix Table S4. Biotin labeled antisense sequences

Name	Sequence 5’-3’
47S rRNA (human)	CGGGAGAGCACGACGTCACCACATCGATCACGAA<br>GAGCCCCCCGGGAGCGGAGGCCGGCCGGCC<br>AGCGAGCCGATCGGCTCCGGCCAACCCCCCACTCC<br>GGGGAAGGGGCGGCGGACAACCCCGCGGAGACG<br>AGAACGCCTGACACGCAC
ACTB (human)	CTGTCACCTTCACCGTTCCAGTTTTTAAATCCTGAG<br>TCAAGCCAAAAAAAAAAAAAAAACCAAAACAAA<br>ACAAAAAAAACAAATAAAGCCATGCCAATCTCATC<br>TTGTTTTCTGCGCAAGTTAGGTTTTGTCAAGAAAG<br>GGTGTAACGCAACTA
