# Supplementary material for: Cytosolic DNA inhibits rDNA transcription by retaining the RNA polymerase I transcription machinery
Source: EMBO J. 2026 May 5;45(12):4153–75. doi: 10.1038/s44318-026-00792-2 (PMC13270134; doi:10.1038/s44318-026-00792-2)
Supplement: Supplementary file 3 — Source data Fig. 1 [file 44318_2026_792_MOESM3_ESM.zip › Source data for Figure 1/Gel data/1D, E, G, I, and J.pdf]

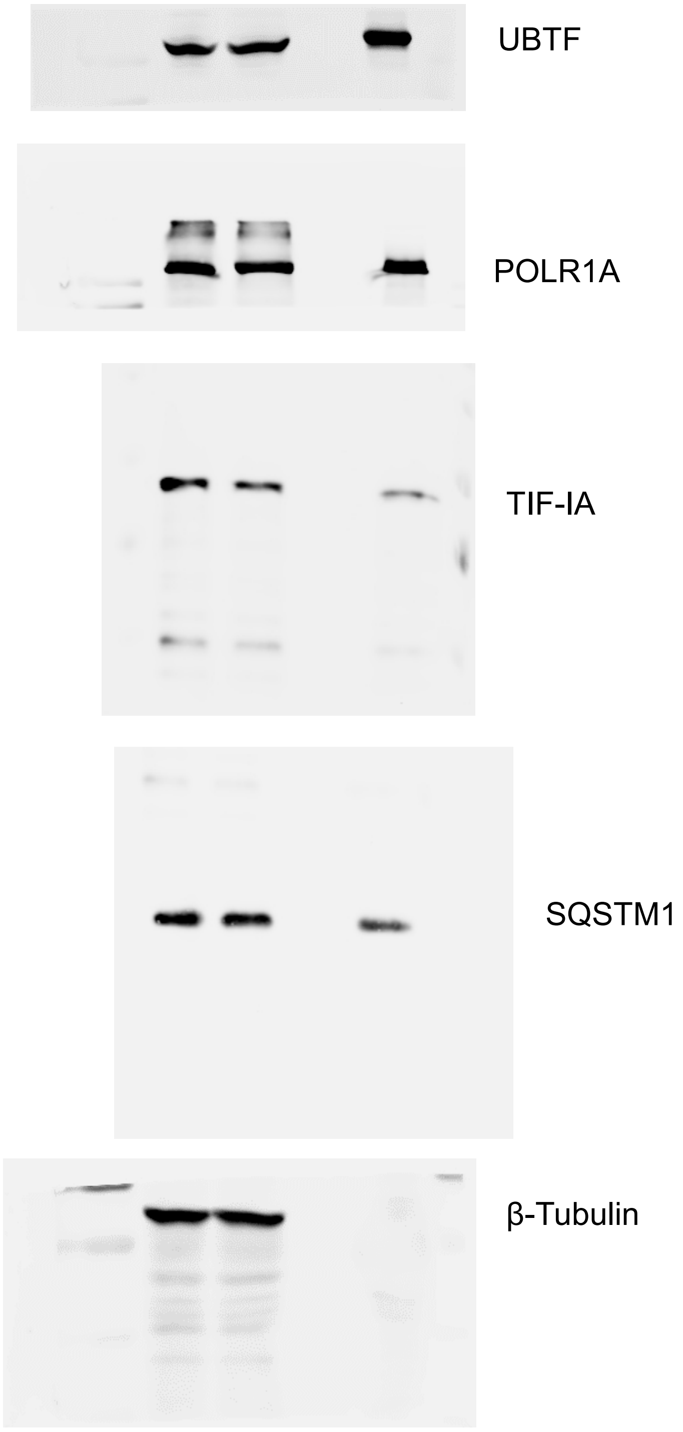

Fig 1D

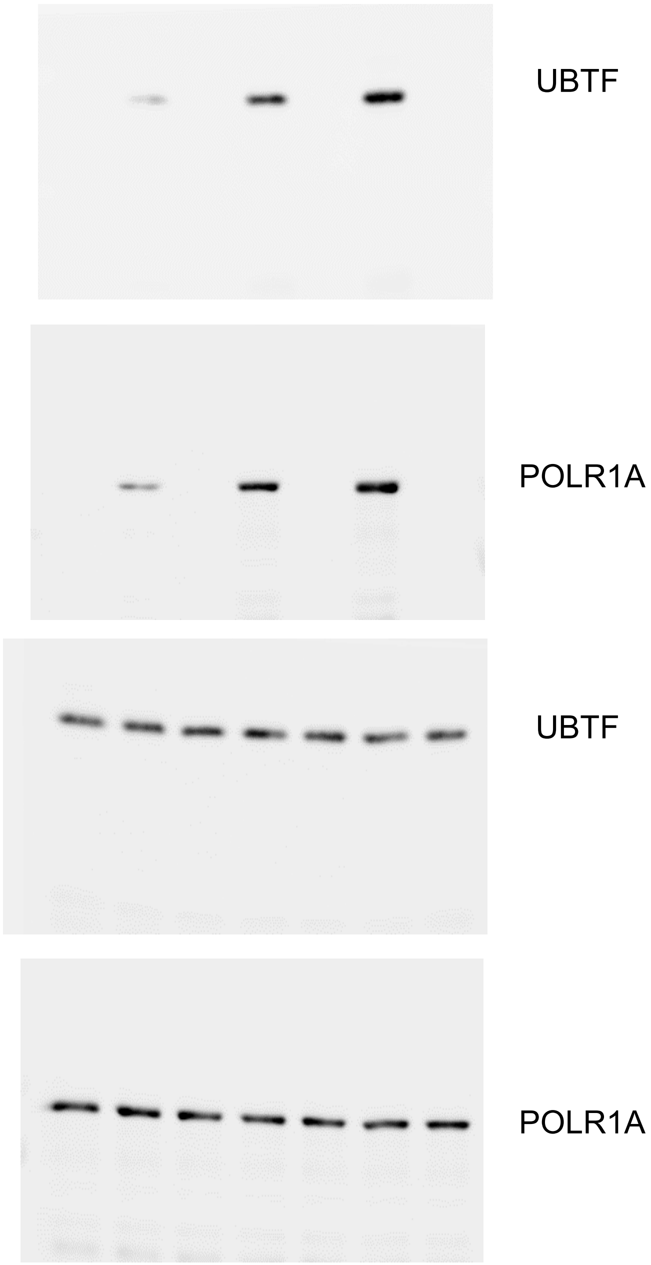

Fig 1E

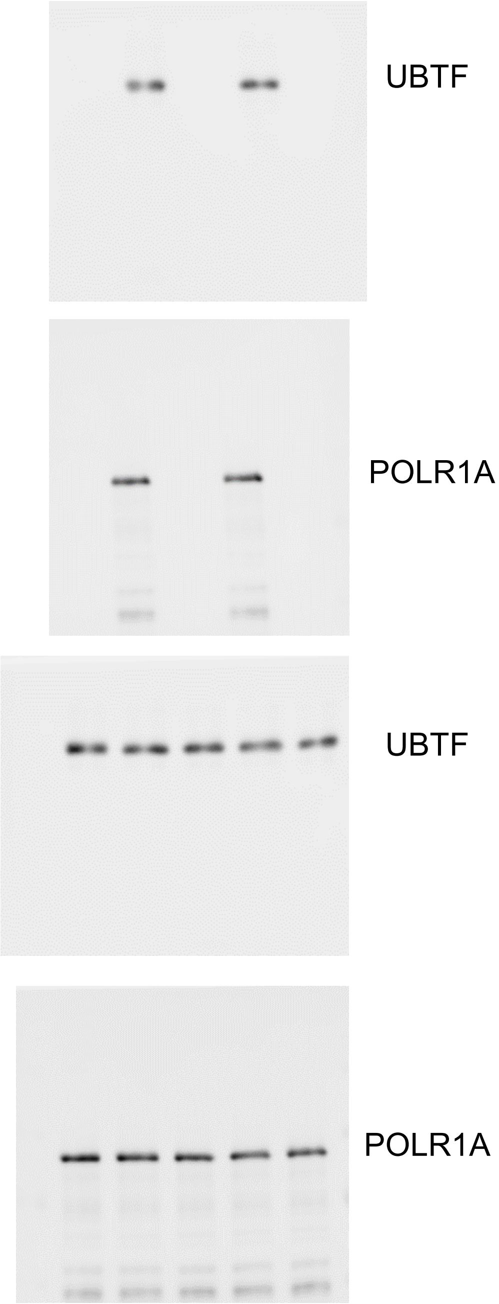

Fig 1G

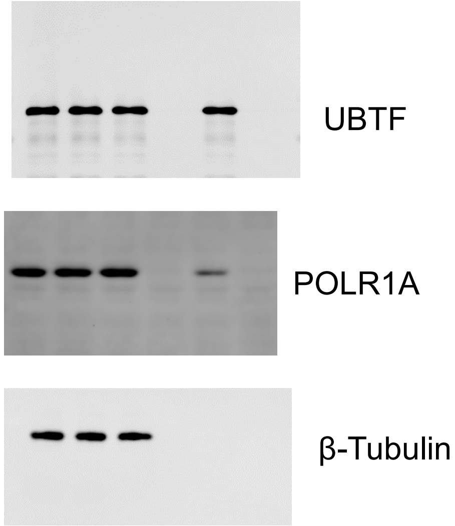

Fig 1I

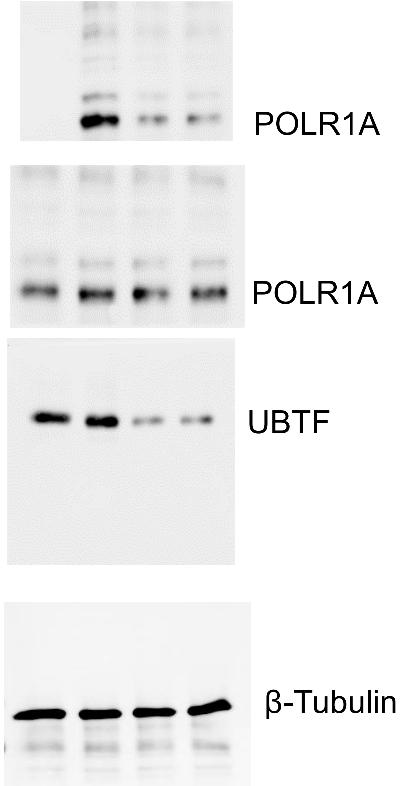

Fig 1J
