# Supplementary figures and images for: Cytosolic DNA inhibits rDNA transcription by retaining the RNA polymerase I transcription machinery (part 1 of 2)
Source: EMBO J. 2026 May 5;45(12):4153–75. doi: 10.1038/s44318-026-00792-2 (PMC13270134; doi:10.1038/s44318-026-00792-2)

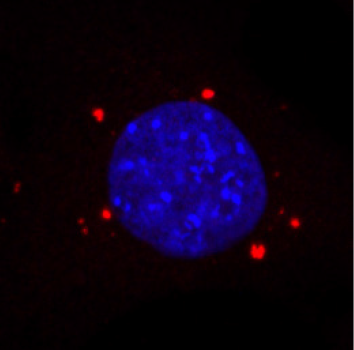

Supplement: Supplementary file 3 — Source data Fig. 1 [file 44318_2026_792_MOESM3_ESM.zip › Source data for Figure 1/Microscopy/1A.tif]

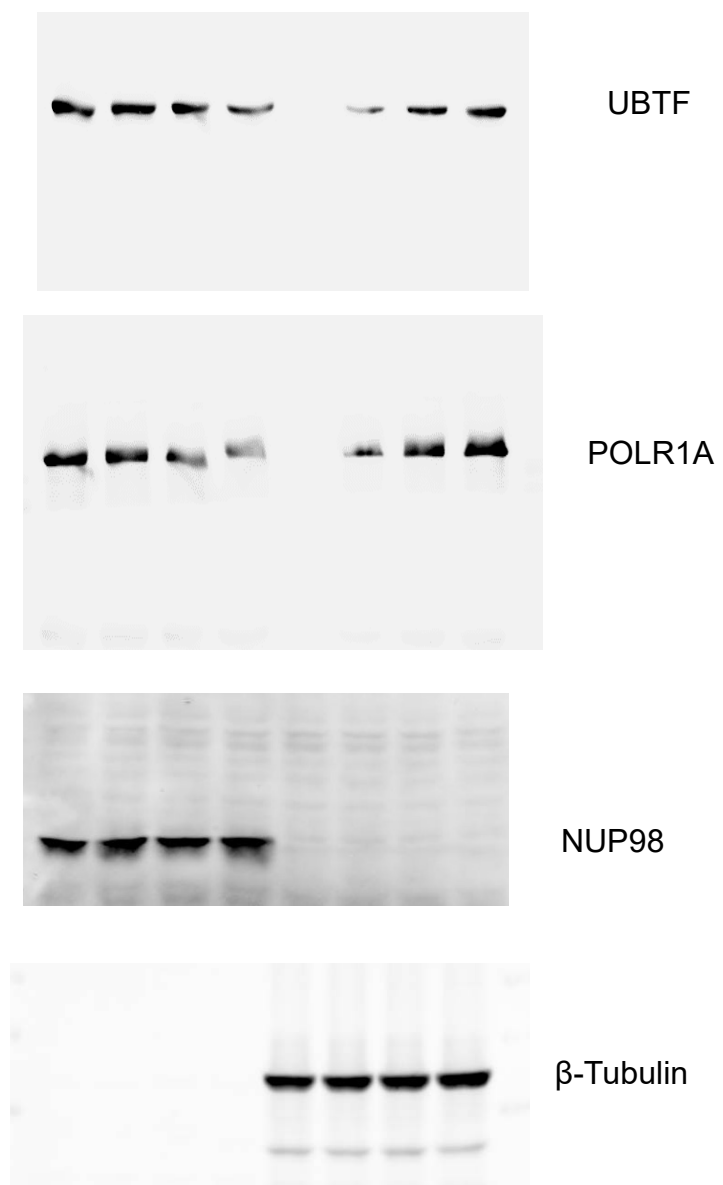

**Fig 2C**

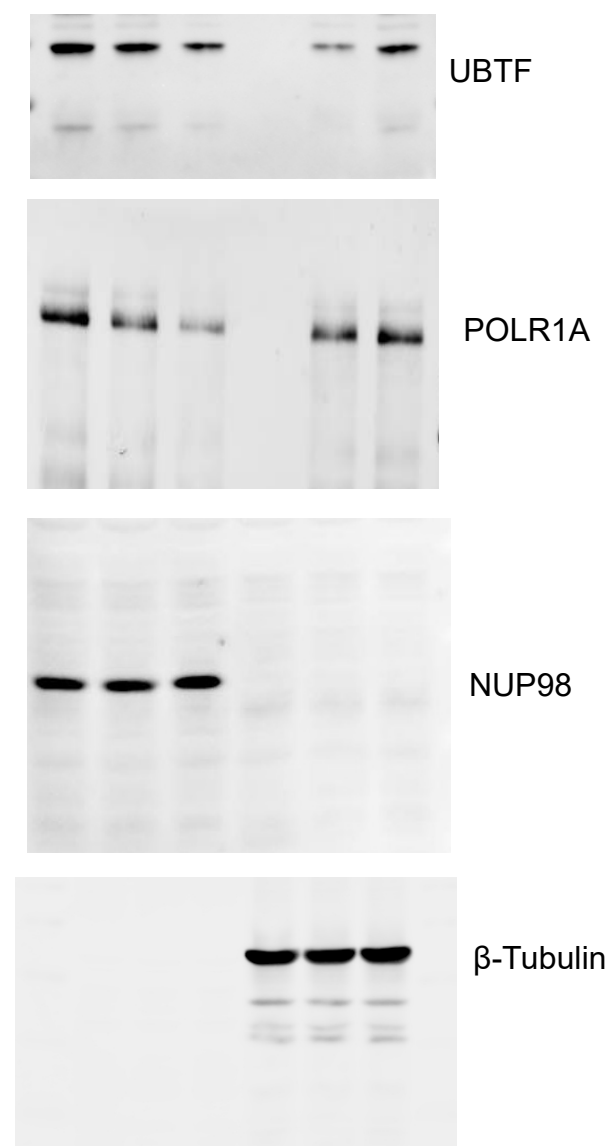

**Fig 2E**

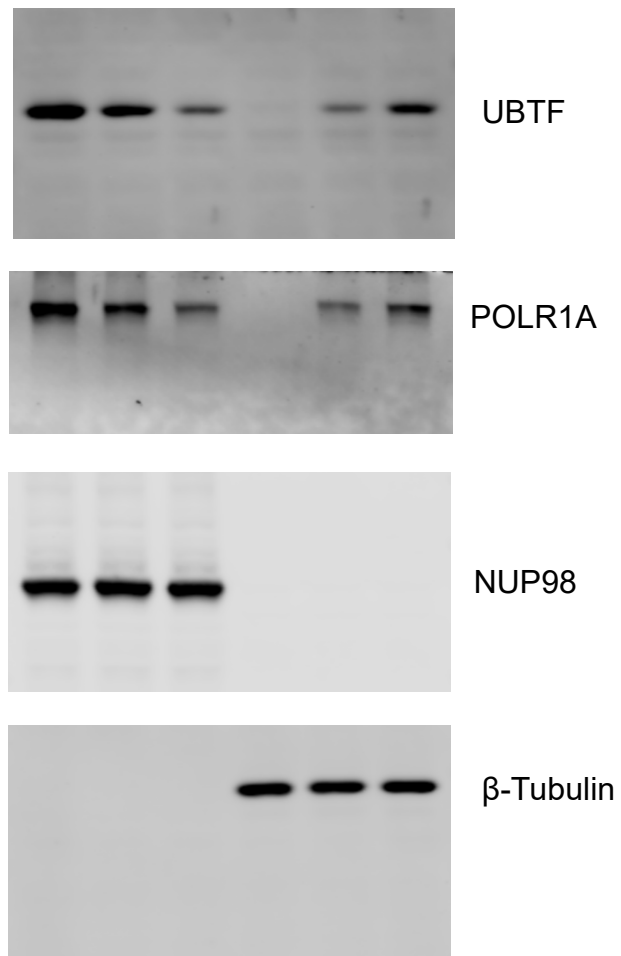

**Fig 2I**

Supplement: Supplementary file 4 — Source data Fig. 2 [file 44318_2026_792_MOESM4_ESM.zip › Source data for Figure 2/Gel data/2C, E, and I.pdf]

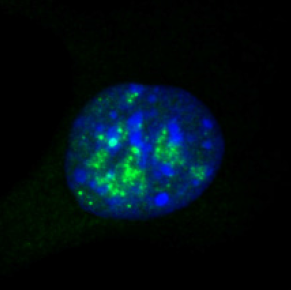

Supplement: Supplementary file 4 — Source data Fig. 2 [file 44318_2026_792_MOESM4_ESM.zip › Source data for Figure 2/Microscopy/2A/2A_1.tif]

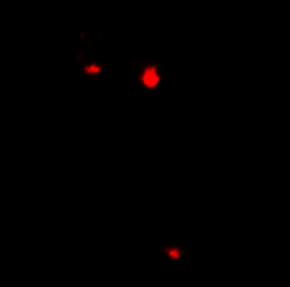

Supplement: Supplementary file 4 — Source data Fig. 2 [file 44318_2026_792_MOESM4_ESM.zip › Source data for Figure 2/Microscopy/2A/2A_10.tif]

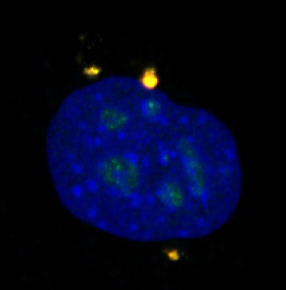

Supplement: Supplementary file 4 — Source data Fig. 2 [file 44318_2026_792_MOESM4_ESM.zip › Source data for Figure 2/Microscopy/2A/2A_11.tif]

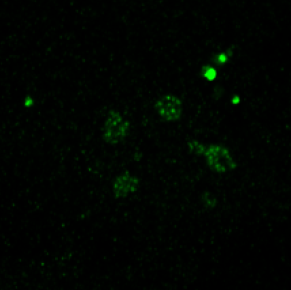

Supplement: Supplementary file 4 — Source data Fig. 2 [file 44318_2026_792_MOESM4_ESM.zip › Source data for Figure 2/Microscopy/2A/2A_12.tif]

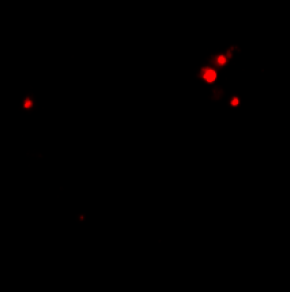

Supplement: Supplementary file 4 — Source data Fig. 2 [file 44318_2026_792_MOESM4_ESM.zip › Source data for Figure 2/Microscopy/2A/2A_13.tif]

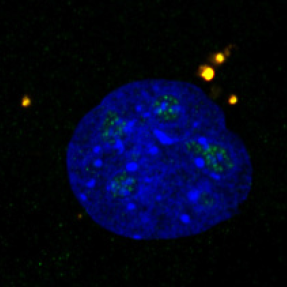

Supplement: Supplementary file 4 — Source data Fig. 2 [file 44318_2026_792_MOESM4_ESM.zip › Source data for Figure 2/Microscopy/2A/2A_14.tif]

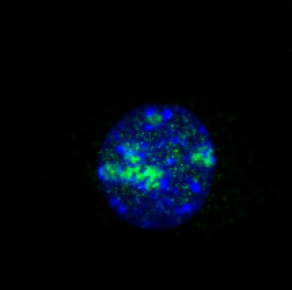

Supplement: Supplementary file 4 — Source data Fig. 2 [file 44318_2026_792_MOESM4_ESM.zip › Source data for Figure 2/Microscopy/2A/2A_2.tif]

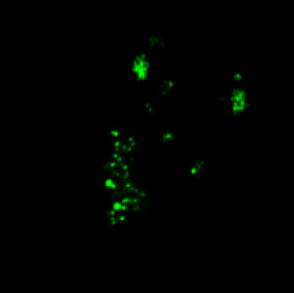

Supplement: Supplementary file 4 — Source data Fig. 2 [file 44318_2026_792_MOESM4_ESM.zip › Source data for Figure 2/Microscopy/2A/2A_3.tif]

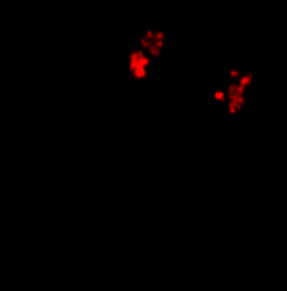

Supplement: Supplementary file 4 — Source data Fig. 2 [file 44318_2026_792_MOESM4_ESM.zip › Source data for Figure 2/Microscopy/2A/2A_4.tif]

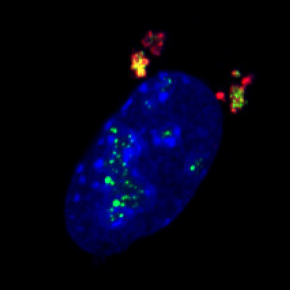

Supplement: Supplementary file 4 — Source data Fig. 2 [file 44318_2026_792_MOESM4_ESM.zip › Source data for Figure 2/Microscopy/2A/2A_5.tif]

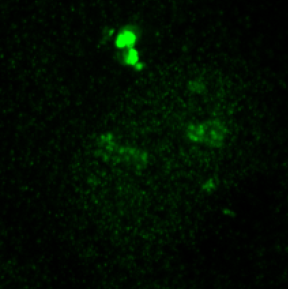

Supplement: Supplementary file 4 — Source data Fig. 2 [file 44318_2026_792_MOESM4_ESM.zip › Source data for Figure 2/Microscopy/2A/2A_6.tif]

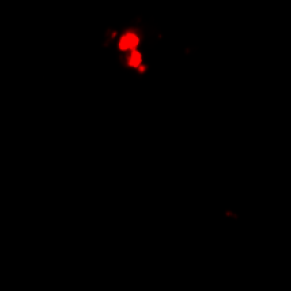

Supplement: Supplementary file 4 — Source data Fig. 2 [file 44318_2026_792_MOESM4_ESM.zip › Source data for Figure 2/Microscopy/2A/2A_7.tif]

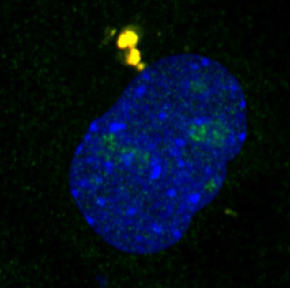

Supplement: Supplementary file 4 — Source data Fig. 2 [file 44318_2026_792_MOESM4_ESM.zip › Source data for Figure 2/Microscopy/2A/2A_8.tif]

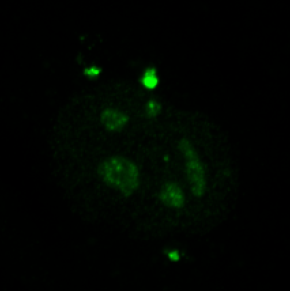

Supplement: Supplementary file 4 — Source data Fig. 2 [file 44318_2026_792_MOESM4_ESM.zip › Source data for Figure 2/Microscopy/2A/2A_9.tif]

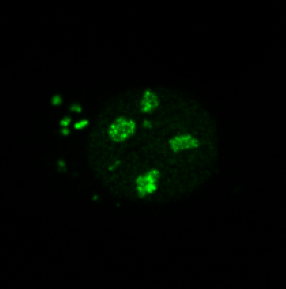

Supplement: Supplementary file 4 — Source data Fig. 2 [file 44318_2026_792_MOESM4_ESM.zip › Source data for Figure 2/Microscopy/2G/2G_1.tif]

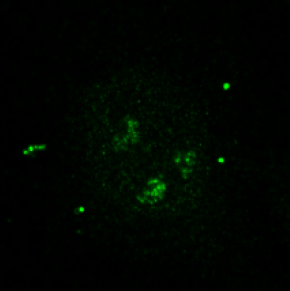

Supplement: Supplementary file 4 — Source data Fig. 2 [file 44318_2026_792_MOESM4_ESM.zip › Source data for Figure 2/Microscopy/2G/2G_10.tif]

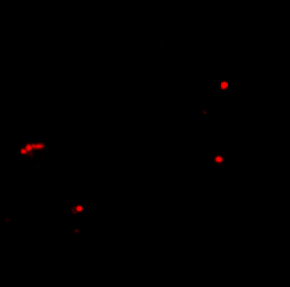

Supplement: Supplementary file 4 — Source data Fig. 2 [file 44318_2026_792_MOESM4_ESM.zip › Source data for Figure 2/Microscopy/2G/2G_11.tif]

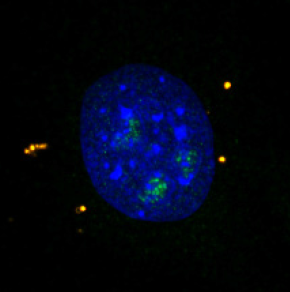

Supplement: Supplementary file 4 — Source data Fig. 2 [file 44318_2026_792_MOESM4_ESM.zip › Source data for Figure 2/Microscopy/2G/2G_12.tif]

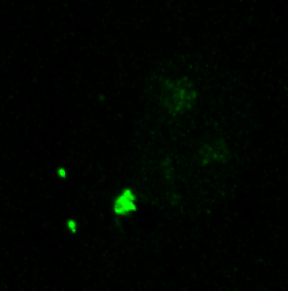

Supplement: Supplementary file 4 — Source data Fig. 2 [file 44318_2026_792_MOESM4_ESM.zip › Source data for Figure 2/Microscopy/2G/2G_13.tif]

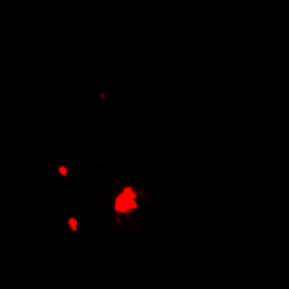

Supplement: Supplementary file 4 — Source data Fig. 2 [file 44318_2026_792_MOESM4_ESM.zip › Source data for Figure 2/Microscopy/2G/2G_14.tif]

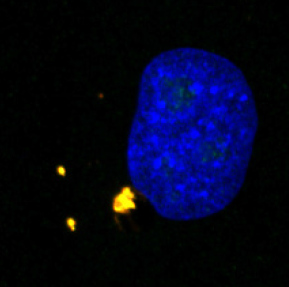

Supplement: Supplementary file 4 — Source data Fig. 2 [file 44318_2026_792_MOESM4_ESM.zip › Source data for Figure 2/Microscopy/2G/2G_15.tif]

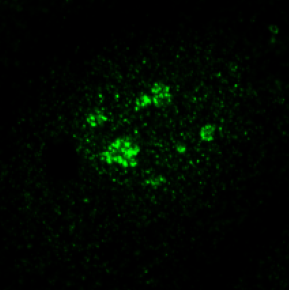

Supplement: Supplementary file 4 — Source data Fig. 2 [file 44318_2026_792_MOESM4_ESM.zip › Source data for Figure 2/Microscopy/2G/2G_16.tif]

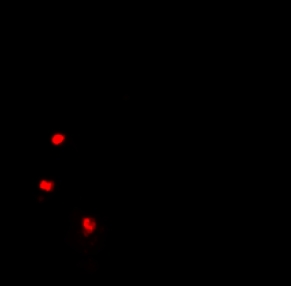

Supplement: Supplementary file 4 — Source data Fig. 2 [file 44318_2026_792_MOESM4_ESM.zip › Source data for Figure 2/Microscopy/2G/2G_17.tif]

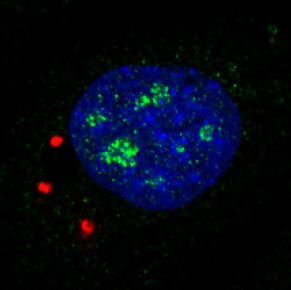

Supplement: Supplementary file 4 — Source data Fig. 2 [file 44318_2026_792_MOESM4_ESM.zip › Source data for Figure 2/Microscopy/2G/2G_18.tif]

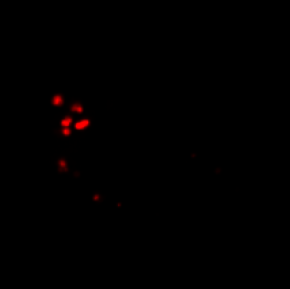

Supplement: Supplementary file 4 — Source data Fig. 2 [file 44318_2026_792_MOESM4_ESM.zip › Source data for Figure 2/Microscopy/2G/2G_2.tif]

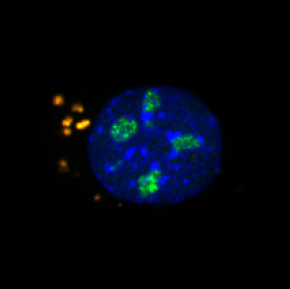

Supplement: Supplementary file 4 — Source data Fig. 2 [file 44318_2026_792_MOESM4_ESM.zip › Source data for Figure 2/Microscopy/2G/2G_3.tif]

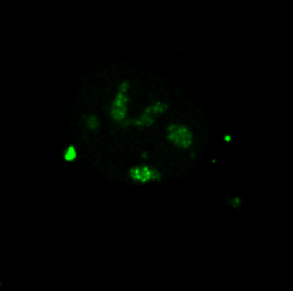

Supplement: Supplementary file 4 — Source data Fig. 2 [file 44318_2026_792_MOESM4_ESM.zip › Source data for Figure 2/Microscopy/2G/2G_4.tif]

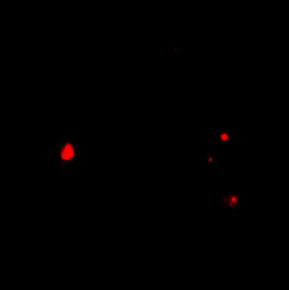

Supplement: Supplementary file 4 — Source data Fig. 2 [file 44318_2026_792_MOESM4_ESM.zip › Source data for Figure 2/Microscopy/2G/2G_5.tif]

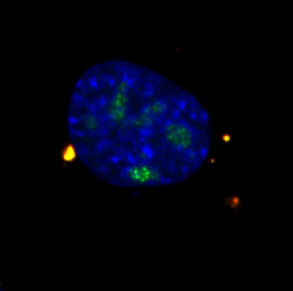

Supplement: Supplementary file 4 — Source data Fig. 2 [file 44318_2026_792_MOESM4_ESM.zip › Source data for Figure 2/Microscopy/2G/2G_6.tif]

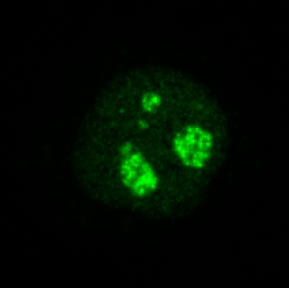

Supplement: Supplementary file 4 — Source data Fig. 2 [file 44318_2026_792_MOESM4_ESM.zip › Source data for Figure 2/Microscopy/2G/2G_7.tif]

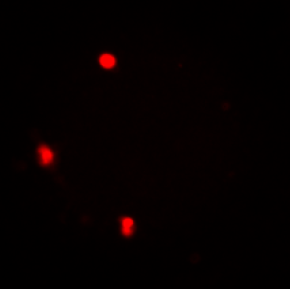

Supplement: Supplementary file 4 — Source data Fig. 2 [file 44318_2026_792_MOESM4_ESM.zip › Source data for Figure 2/Microscopy/2G/2G_8.tif]

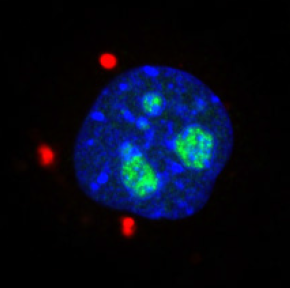

Supplement: Supplementary file 4 — Source data Fig. 2 [file 44318_2026_792_MOESM4_ESM.zip › Source data for Figure 2/Microscopy/2G/2G_9.tif]

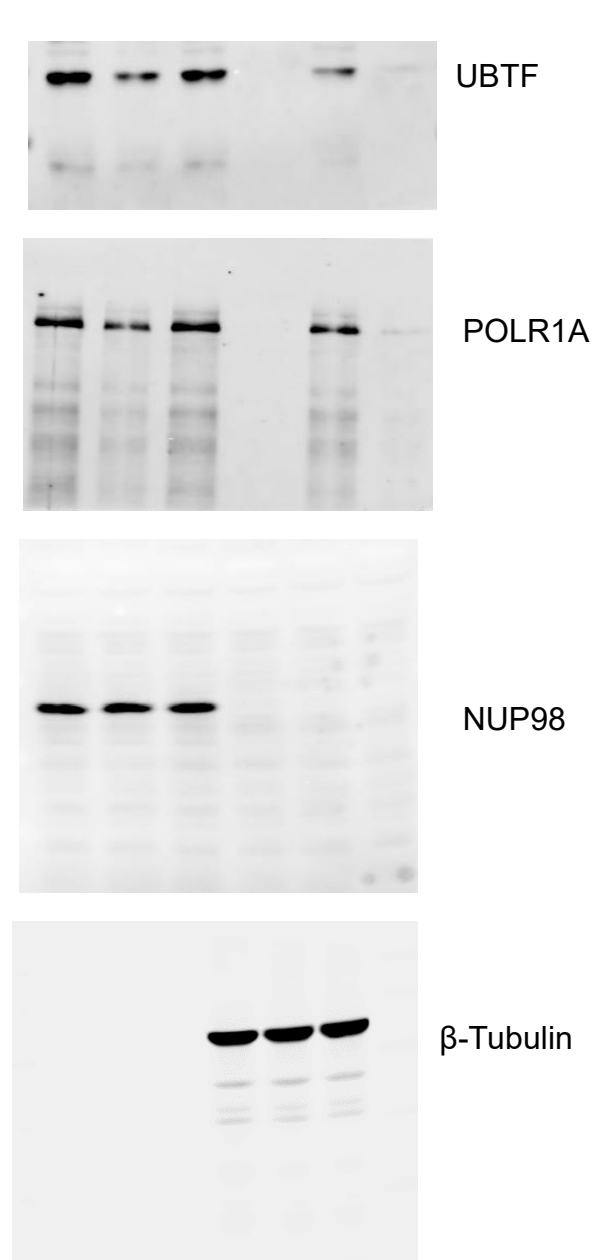

**Fig 3H**

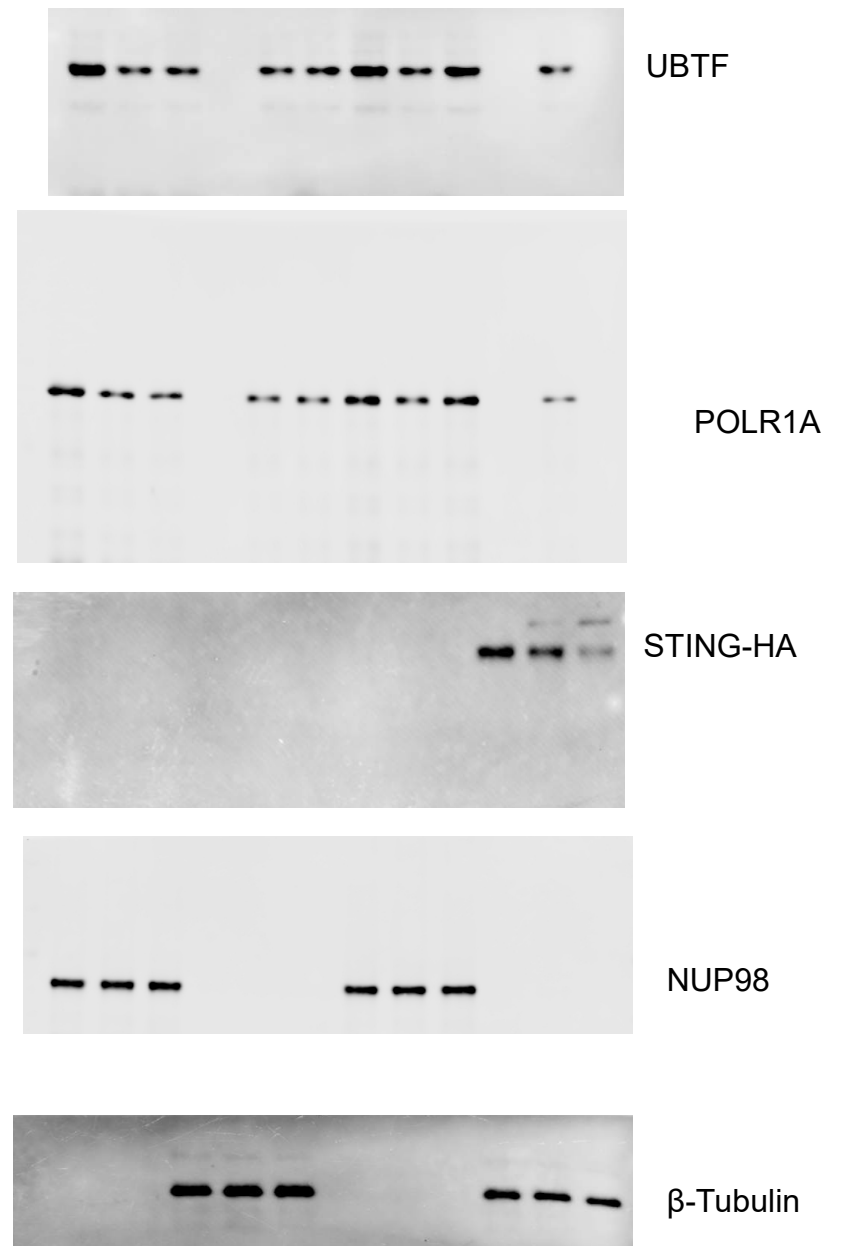

**Fig 3I**

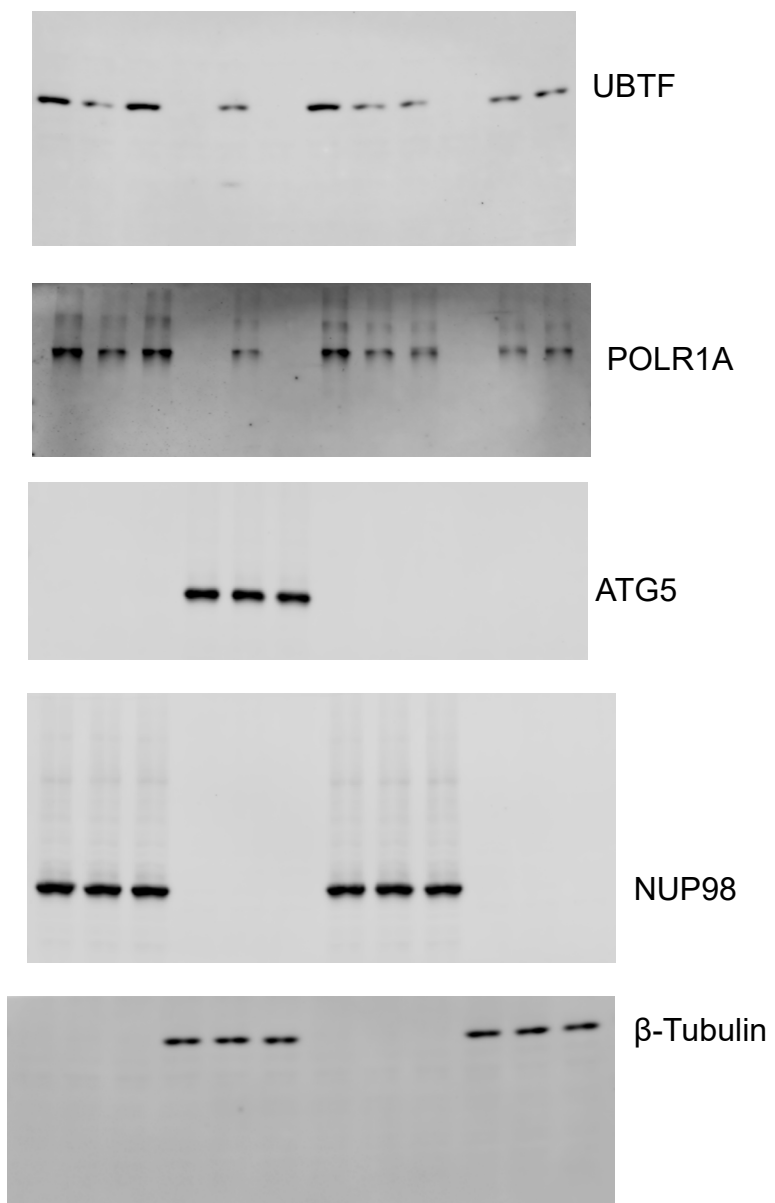

**Fig 3J**

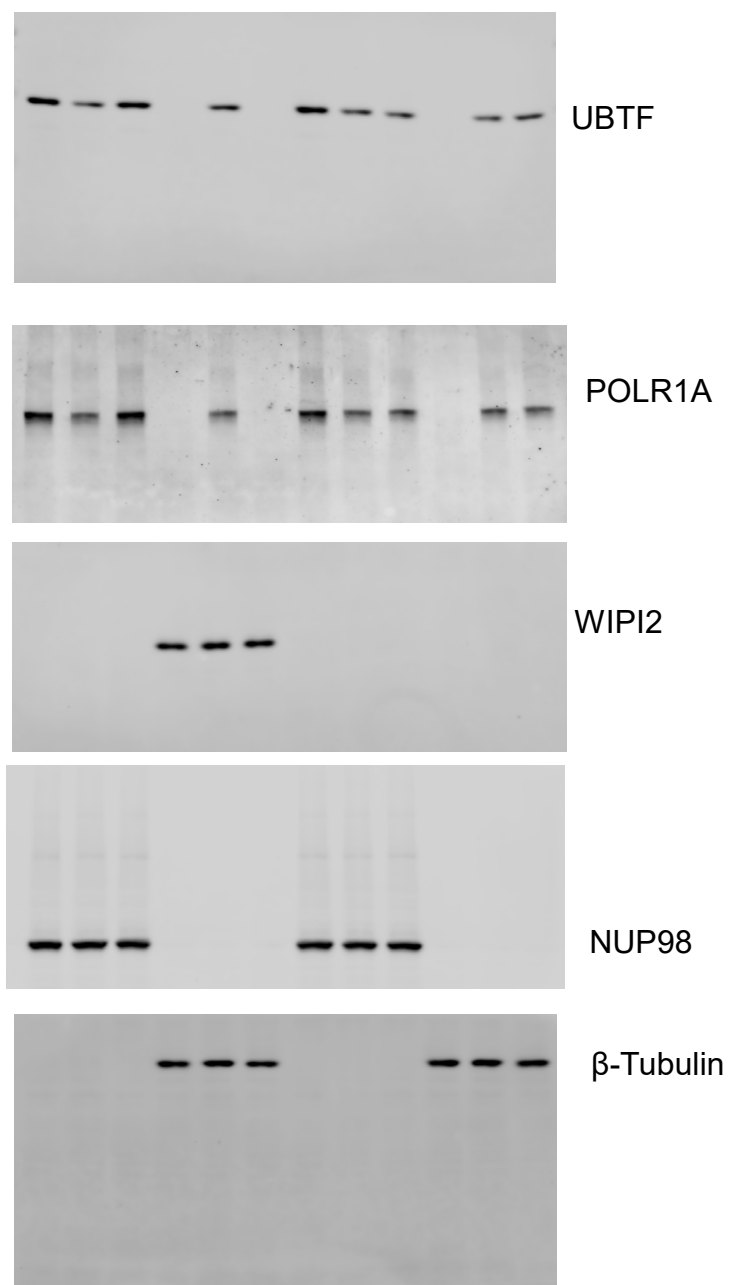

**Fig 3K**

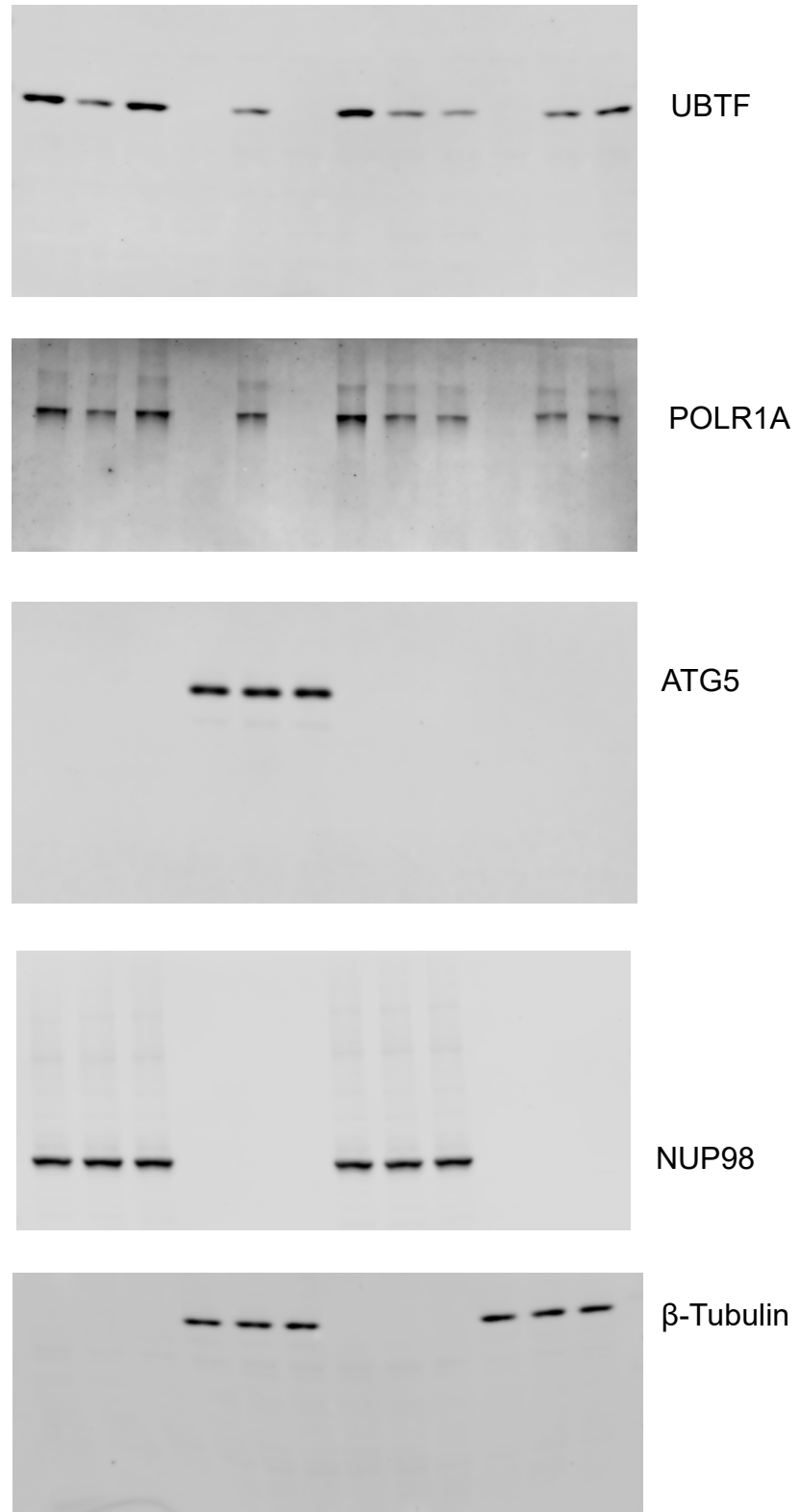

**Fig 3L**

Supplement: Supplementary file 5 — Source data Fig. 3 [file 44318_2026_792_MOESM5_ESM.zip › Source data for Figure 3/Gel data/3H-L.pdf]

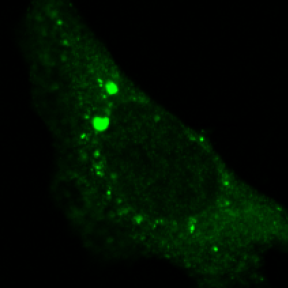

Supplement: Supplementary file 5 — Source data Fig. 3 [file 44318_2026_792_MOESM5_ESM.zip › Source data for Figure 3/Microscopy/3A/3A_1.tif]

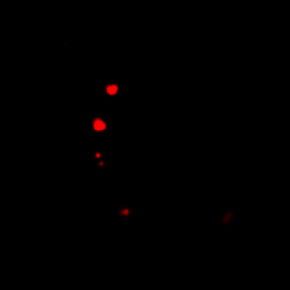

Supplement: Supplementary file 5 — Source data Fig. 3 [file 44318_2026_792_MOESM5_ESM.zip › Source data for Figure 3/Microscopy/3A/3A_2.tif]

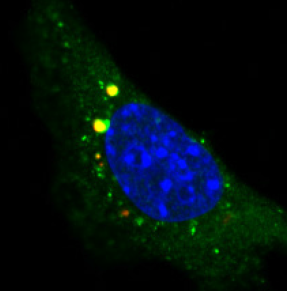

Supplement: Supplementary file 5 — Source data Fig. 3 [file 44318_2026_792_MOESM5_ESM.zip › Source data for Figure 3/Microscopy/3A/3A_3.tif]

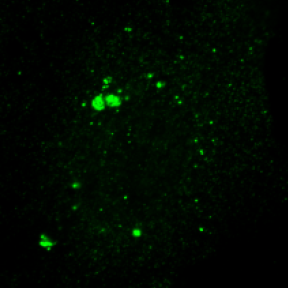

Supplement: Supplementary file 5 — Source data Fig. 3 [file 44318_2026_792_MOESM5_ESM.zip › Source data for Figure 3/Microscopy/3A/3A_4.tif]

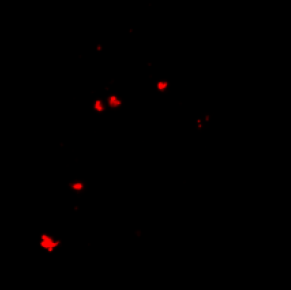

Supplement: Supplementary file 5 — Source data Fig. 3 [file 44318_2026_792_MOESM5_ESM.zip › Source data for Figure 3/Microscopy/3A/3A_5.tif]

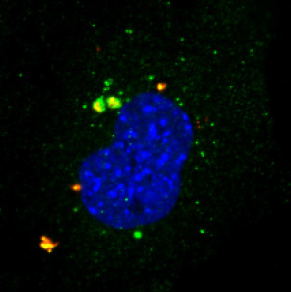

Supplement: Supplementary file 5 — Source data Fig. 3 [file 44318_2026_792_MOESM5_ESM.zip › Source data for Figure 3/Microscopy/3A/3A_6.tif]

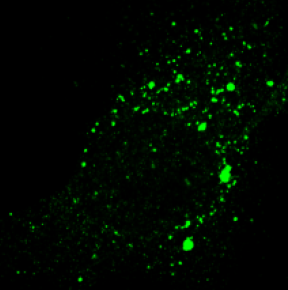

Supplement: Supplementary file 5 — Source data Fig. 3 [file 44318_2026_792_MOESM5_ESM.zip › Source data for Figure 3/Microscopy/3A/3A_7.tif]

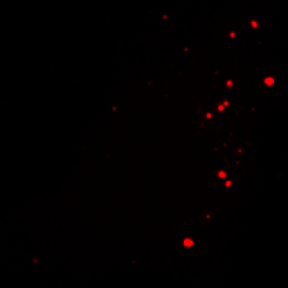

Supplement: Supplementary file 5 — Source data Fig. 3 [file 44318_2026_792_MOESM5_ESM.zip › Source data for Figure 3/Microscopy/3A/3A_8.tif]

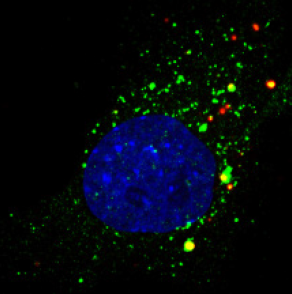

Supplement: Supplementary file 5 — Source data Fig. 3 [file 44318_2026_792_MOESM5_ESM.zip › Source data for Figure 3/Microscopy/3A/3A_9.tif]

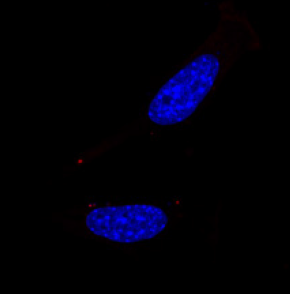

Supplement: Supplementary file 5 — Source data Fig. 3 [file 44318_2026_792_MOESM5_ESM.zip › Source data for Figure 3/Microscopy/3B/3B_1.tif]

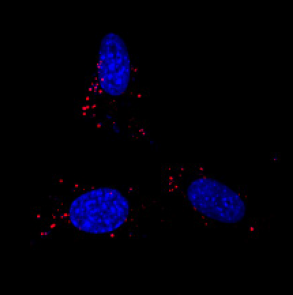

Supplement: Supplementary file 5 — Source data Fig. 3 [file 44318_2026_792_MOESM5_ESM.zip › Source data for Figure 3/Microscopy/3B/3B_2.tif]

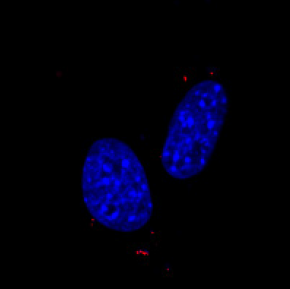

Supplement: Supplementary file 5 — Source data Fig. 3 [file 44318_2026_792_MOESM5_ESM.zip › Source data for Figure 3/Microscopy/3B/3B_3.tif]

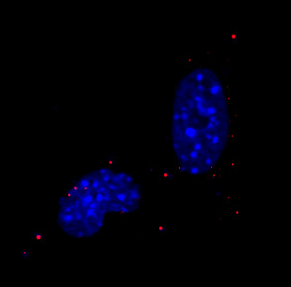

Supplement: Supplementary file 5 — Source data Fig. 3 [file 44318_2026_792_MOESM5_ESM.zip › Source data for Figure 3/Microscopy/3B/3B_4.tif]

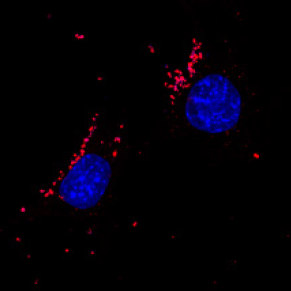

Supplement: Supplementary file 5 — Source data Fig. 3 [file 44318_2026_792_MOESM5_ESM.zip › Source data for Figure 3/Microscopy/3B/3B_5.tif]

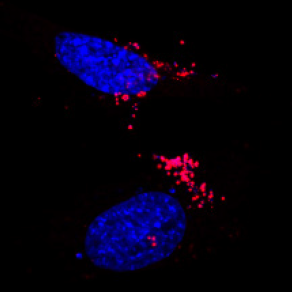

Supplement: Supplementary file 5 — Source data Fig. 3 [file 44318_2026_792_MOESM5_ESM.zip › Source data for Figure 3/Microscopy/3B/3B_6.tif]

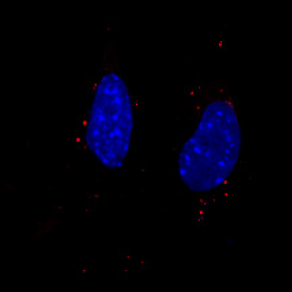

Supplement: Supplementary file 5 — Source data Fig. 3 [file 44318_2026_792_MOESM5_ESM.zip › Source data for Figure 3/Microscopy/3B/3B_7.tif]

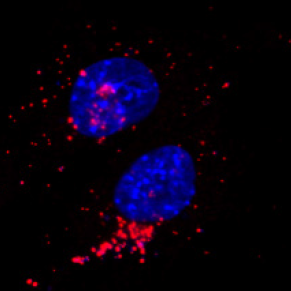

Supplement: Supplementary file 5 — Source data Fig. 3 [file 44318_2026_792_MOESM5_ESM.zip › Source data for Figure 3/Microscopy/3B/3B_8.tif]

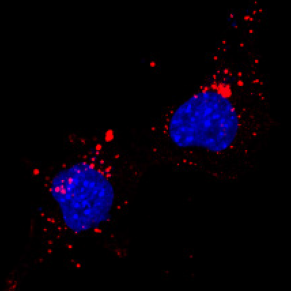

Supplement: Supplementary file 5 — Source data Fig. 3 [file 44318_2026_792_MOESM5_ESM.zip › Source data for Figure 3/Microscopy/3B/3B_9.tif]

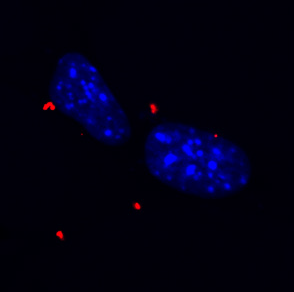

Supplement: Supplementary file 5 — Source data Fig. 3 [file 44318_2026_792_MOESM5_ESM.zip › Source data for Figure 3/Microscopy/3D/3D_1.tif]

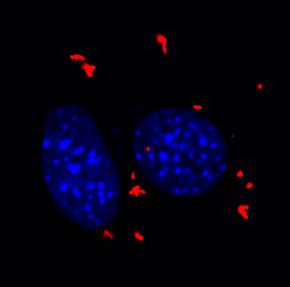

Supplement: Supplementary file 5 — Source data Fig. 3 [file 44318_2026_792_MOESM5_ESM.zip › Source data for Figure 3/Microscopy/3D/3D_2.tif]

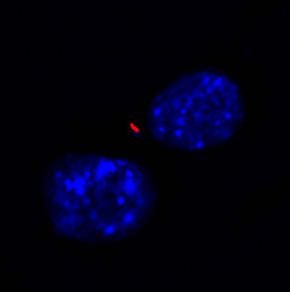

Supplement: Supplementary file 5 — Source data Fig. 3 [file 44318_2026_792_MOESM5_ESM.zip › Source data for Figure 3/Microscopy/3D/3D_3.tif]

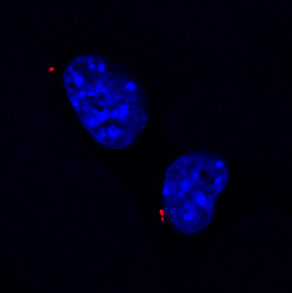

Supplement: Supplementary file 5 — Source data Fig. 3 [file 44318_2026_792_MOESM5_ESM.zip › Source data for Figure 3/Microscopy/3D/3D_4.tif]

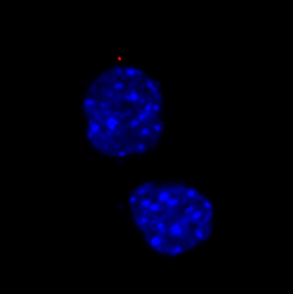

Supplement: Supplementary file 5 — Source data Fig. 3 [file 44318_2026_792_MOESM5_ESM.zip › Source data for Figure 3/Microscopy/3F/3F_1.tif]

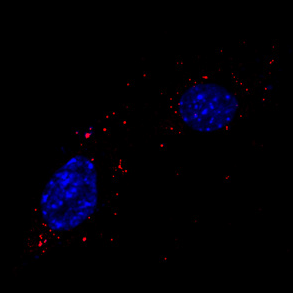

Supplement: Supplementary file 5 — Source data Fig. 3 [file 44318_2026_792_MOESM5_ESM.zip › Source data for Figure 3/Microscopy/3F/3F_2.tif]

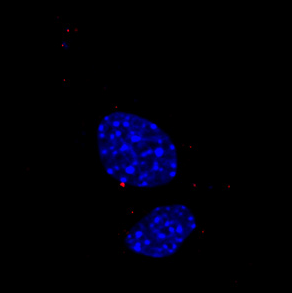

Supplement: Supplementary file 5 — Source data Fig. 3 [file 44318_2026_792_MOESM5_ESM.zip › Source data for Figure 3/Microscopy/3F/3F_3.tif]

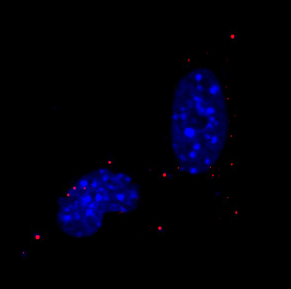

Supplement: Supplementary file 5 — Source data Fig. 3 [file 44318_2026_792_MOESM5_ESM.zip › Source data for Figure 3/Microscopy/3F/3F_4.tif]

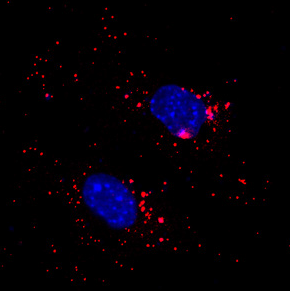

Supplement: Supplementary file 5 — Source data Fig. 3 [file 44318_2026_792_MOESM5_ESM.zip › Source data for Figure 3/Microscopy/3F/3F_5.tif]

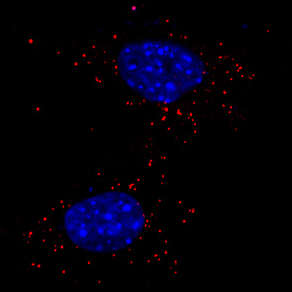

Supplement: Supplementary file 5 — Source data Fig. 3 [file 44318_2026_792_MOESM5_ESM.zip › Source data for Figure 3/Microscopy/3F/3F_6.tif]

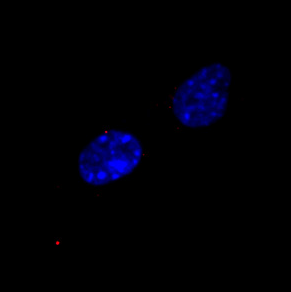

Supplement: Supplementary file 5 — Source data Fig. 3 [file 44318_2026_792_MOESM5_ESM.zip › Source data for Figure 3/Microscopy/3F/3F_7.tif]

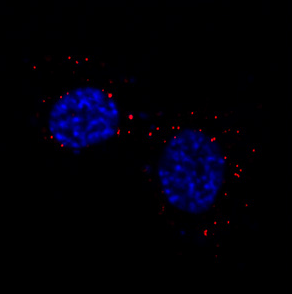

Supplement: Supplementary file 5 — Source data Fig. 3 [file 44318_2026_792_MOESM5_ESM.zip › Source data for Figure 3/Microscopy/3F/3F_8.tif]

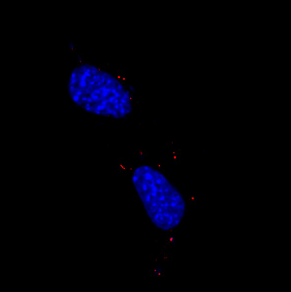

Supplement: Supplementary file 5 — Source data Fig. 3 [file 44318_2026_792_MOESM5_ESM.zip › Source data for Figure 3/Microscopy/3F/3F_9.tif]

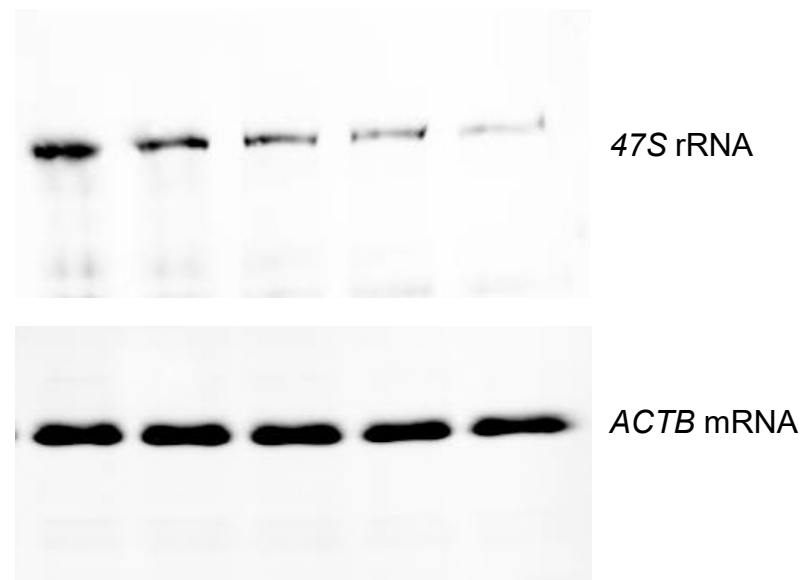

**Fig 4F**

Supplement: Supplementary file 6 — Source data Fig. 4 [file 44318_2026_792_MOESM6_ESM.zip › Source data for Figure 4/Gel data/4F.pdf]

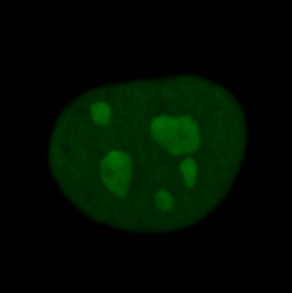

Supplement: Supplementary file 6 — Source data Fig. 4 [file 44318_2026_792_MOESM6_ESM.zip › Source data for Figure 4/Microscopy/4C/4C_1.tif]

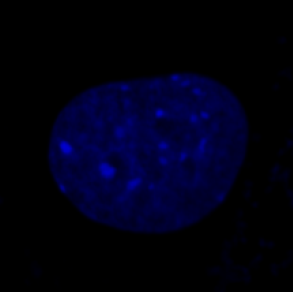

Supplement: Supplementary file 6 — Source data Fig. 4 [file 44318_2026_792_MOESM6_ESM.zip › Source data for Figure 4/Microscopy/4C/4C_2.tif]

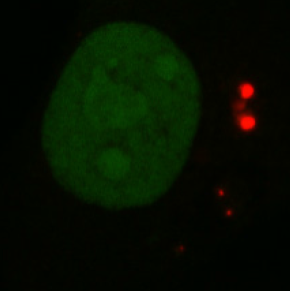

Supplement: Supplementary file 6 — Source data Fig. 4 [file 44318_2026_792_MOESM6_ESM.zip › Source data for Figure 4/Microscopy/4C/4C_3.tif]

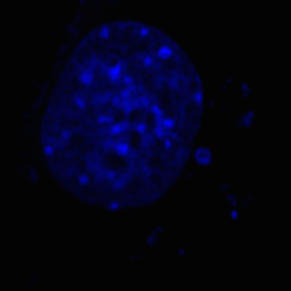

Supplement: Supplementary file 6 — Source data Fig. 4 [file 44318_2026_792_MOESM6_ESM.zip › Source data for Figure 4/Microscopy/4C/4C_4.tif]

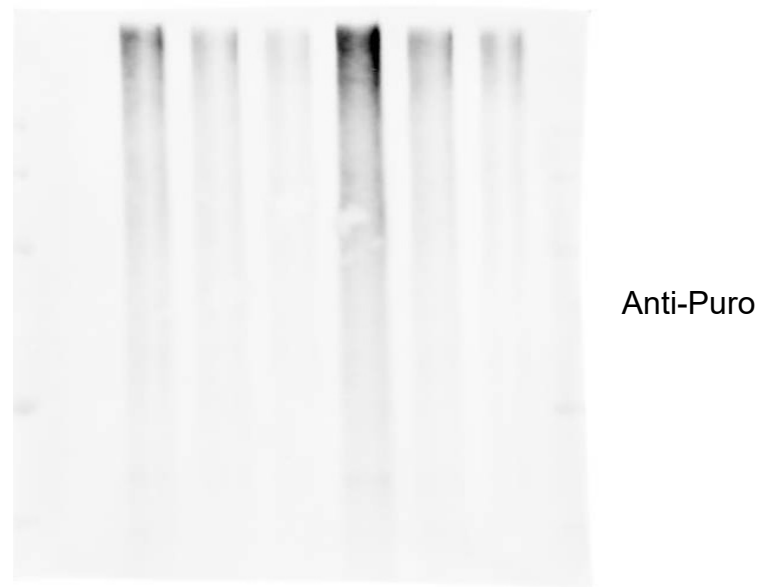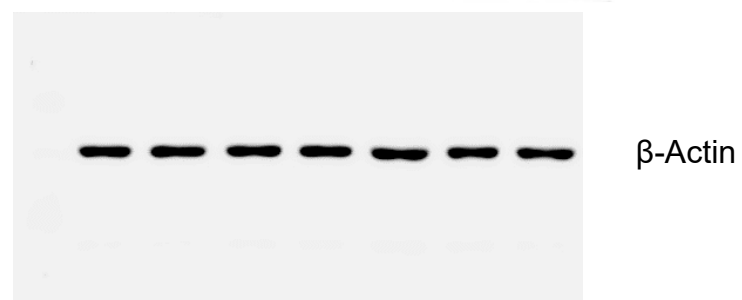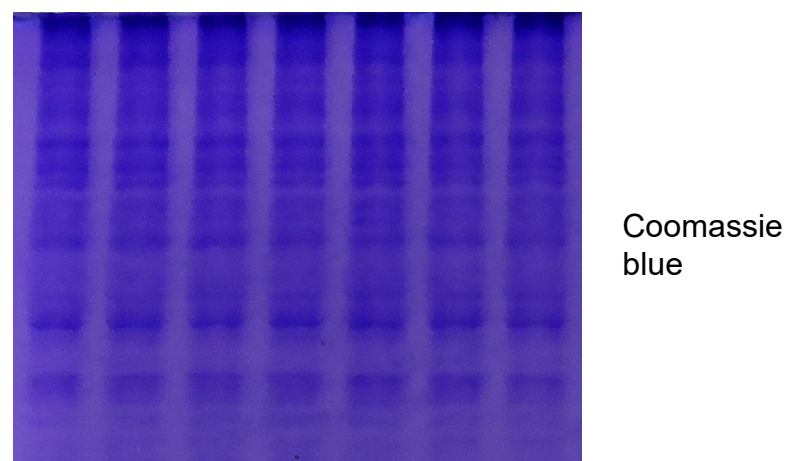

**Fig 5A**

Supplement: Supplementary file 7 — Source data Fig. 5 [file 44318_2026_792_MOESM7_ESM.zip › Source data for Figure 5/Gel data/5A.pdf]

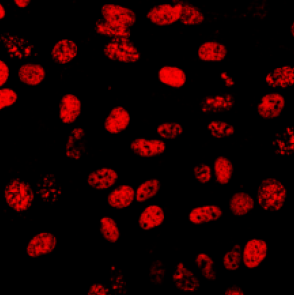

Supplement: Supplementary file 7 — Source data Fig. 5 [file 44318_2026_792_MOESM7_ESM.zip › Source data for Figure 5/Microscopy/5D/5D_1.tif]

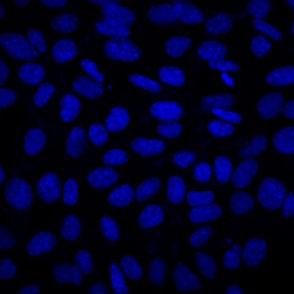

Supplement: Supplementary file 7 — Source data Fig. 5 [file 44318_2026_792_MOESM7_ESM.zip › Source data for Figure 5/Microscopy/5D/5D_2.tif]

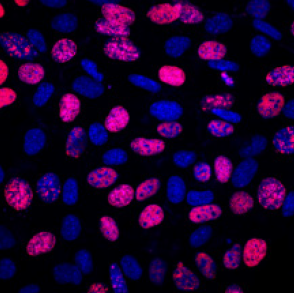

Supplement: Supplementary file 7 — Source data Fig. 5 [file 44318_2026_792_MOESM7_ESM.zip › Source data for Figure 5/Microscopy/5D/5D_3.tif]

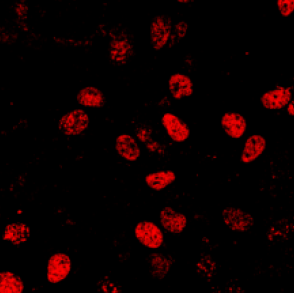

Supplement: Supplementary file 7 — Source data Fig. 5 [file 44318_2026_792_MOESM7_ESM.zip › Source data for Figure 5/Microscopy/5D/5D_4.tif]

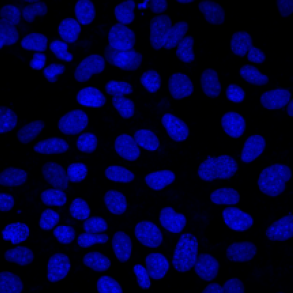

Supplement: Supplementary file 7 — Source data Fig. 5 [file 44318_2026_792_MOESM7_ESM.zip › Source data for Figure 5/Microscopy/5D/5D_5.tif]

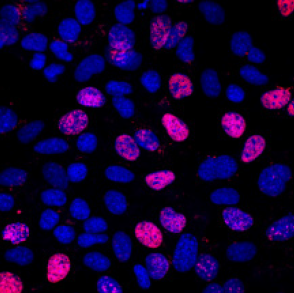

Supplement: Supplementary file 7 — Source data Fig. 5 [file 44318_2026_792_MOESM7_ESM.zip › Source data for Figure 5/Microscopy/5D/5D_6.tif]

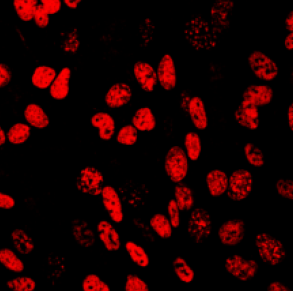

Supplement: Supplementary file 7 — Source data Fig. 5 [file 44318_2026_792_MOESM7_ESM.zip › Source data for Figure 5/Microscopy/5D/5D_7.tif]

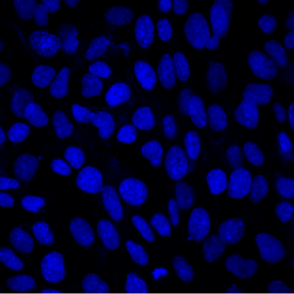

Supplement: Supplementary file 7 — Source data Fig. 5 [file 44318_2026_792_MOESM7_ESM.zip › Source data for Figure 5/Microscopy/5D/5D_8.tif]

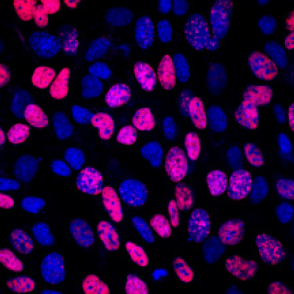

Supplement: Supplementary file 7 — Source data Fig. 5 [file 44318_2026_792_MOESM7_ESM.zip › Source data for Figure 5/Microscopy/5D/5D_9.tif]

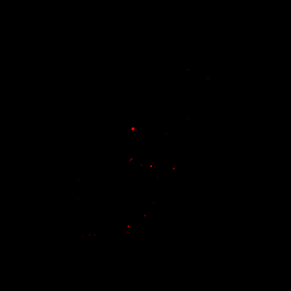

Supplement: Supplementary file 8 — Expanded View and Appendix Source Data [file 44318_2026_792_MOESM8_ESM.zip › Source data for Appendix Figure S1/Microscopy/S1/S1_1.tif]

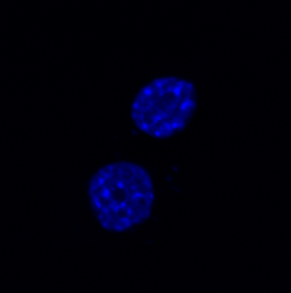

Supplement: Supplementary file 8 — Expanded View and Appendix Source Data [file 44318_2026_792_MOESM8_ESM.zip › Source data for Appendix Figure S1/Microscopy/S1/S1_2.tif]

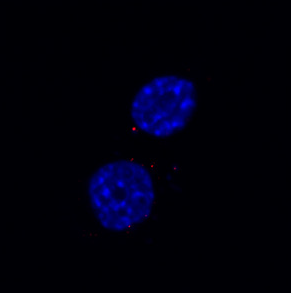

Supplement: Supplementary file 8 — Expanded View and Appendix Source Data [file 44318_2026_792_MOESM8_ESM.zip › Source data for Appendix Figure S1/Microscopy/S1/S1_3.tif]

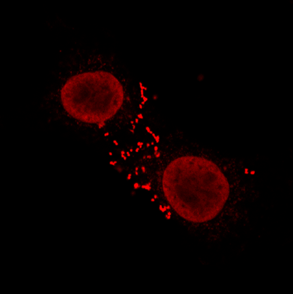

Supplement: Supplementary file 8 — Expanded View and Appendix Source Data [file 44318_2026_792_MOESM8_ESM.zip › Source data for Appendix Figure S1/Microscopy/S1/S1_4.tif]

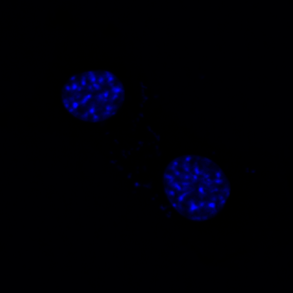

Supplement: Supplementary file 8 — Expanded View and Appendix Source Data [file 44318_2026_792_MOESM8_ESM.zip › Source data for Appendix Figure S1/Microscopy/S1/S1_5.tif]

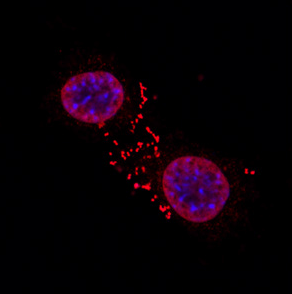

Supplement: Supplementary file 8 — Expanded View and Appendix Source Data [file 44318_2026_792_MOESM8_ESM.zip › Source data for Appendix Figure S1/Microscopy/S1/S1_6.tif]

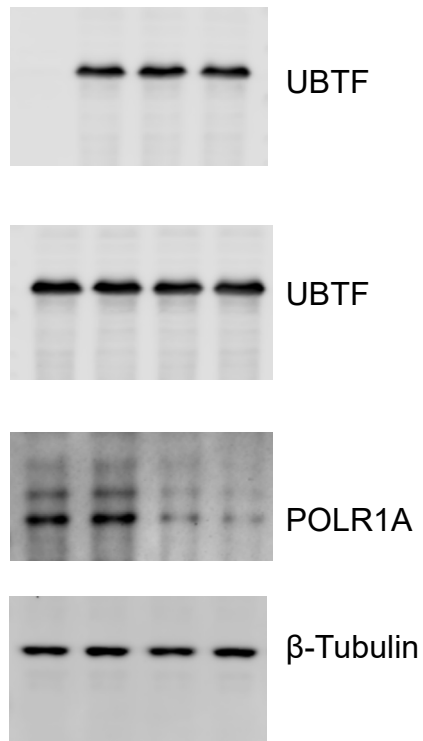

**Fig EV1**

Supplement: Supplementary file 8 — Expanded View and Appendix Source Data [file 44318_2026_792_MOESM8_ESM.zip › Source data for Figure EV1/Gel data/EV1.pdf]

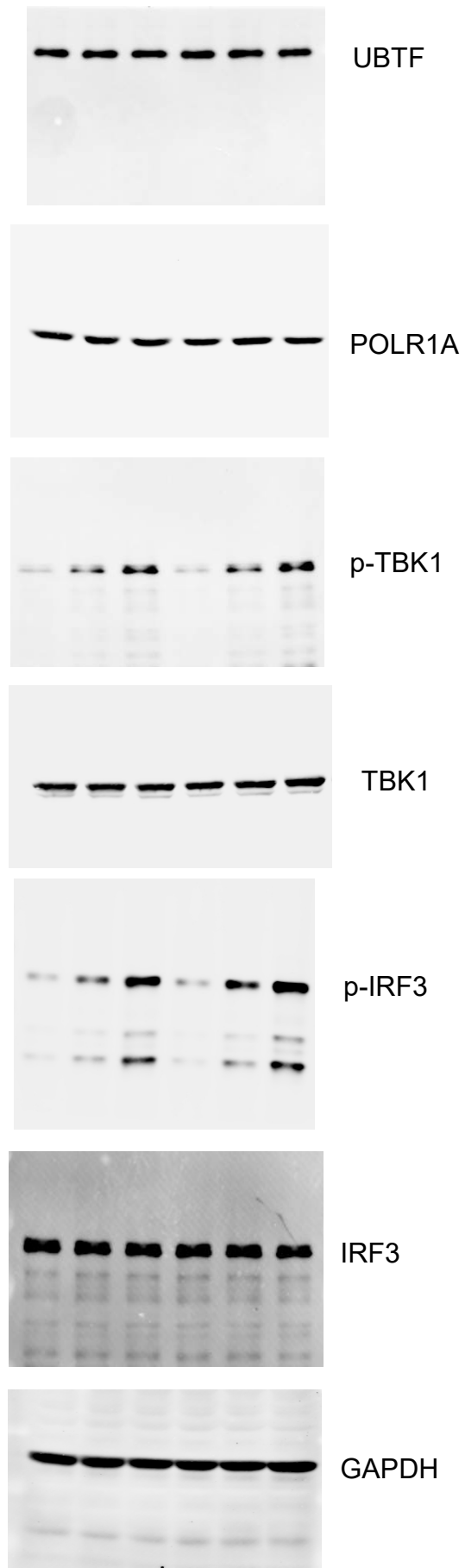

**Fig EV2A**

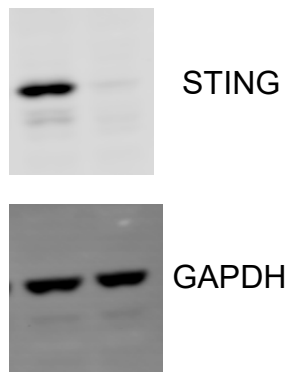

**Fig EV2B**

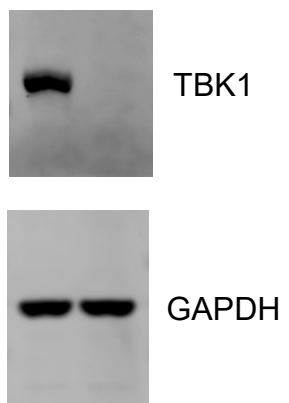

**Fig EV2D**

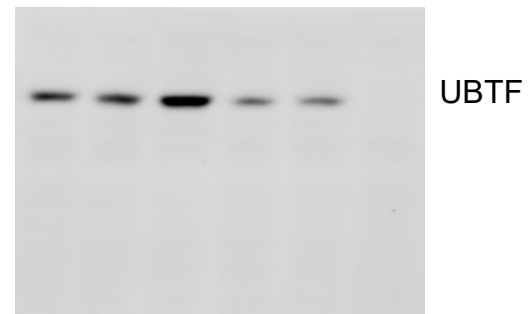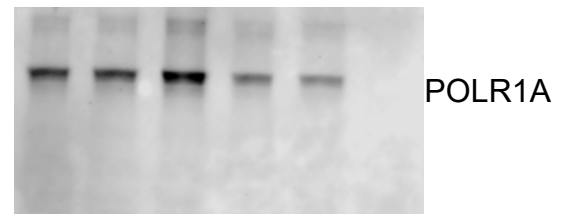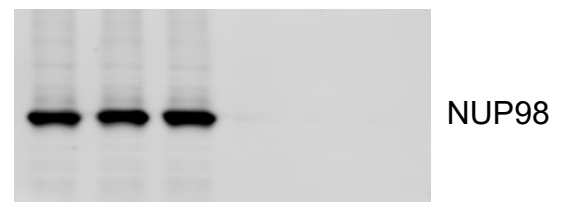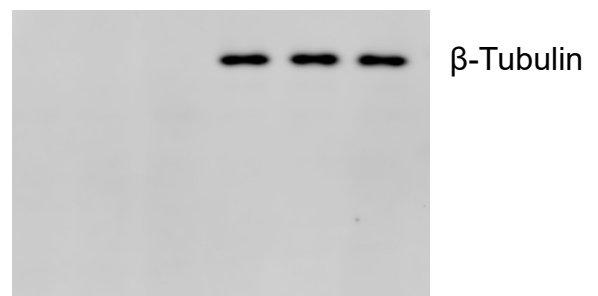

**Fig EV2F**

Supplement: Supplementary file 8 — Expanded View and Appendix Source Data [file 44318_2026_792_MOESM8_ESM.zip › Source data for Figure EV2/Gel data/EV2A, B, D, and F.pdf]

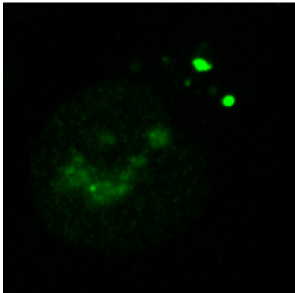

Supplement: Supplementary file 8 — Expanded View and Appendix Source Data [file 44318_2026_792_MOESM8_ESM.zip › Source data for Figure EV2/Microscopy/EV2E_1.tif]

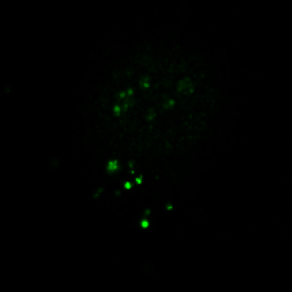

Supplement: Supplementary file 8 — Expanded View and Appendix Source Data [file 44318_2026_792_MOESM8_ESM.zip › Source data for Figure EV2/Microscopy/EV2E_10.tif]

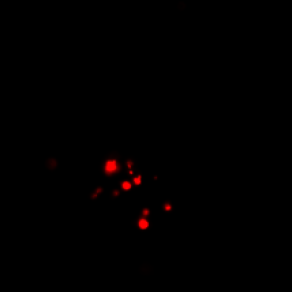

Supplement: Supplementary file 8 — Expanded View and Appendix Source Data [file 44318_2026_792_MOESM8_ESM.zip › Source data for Figure EV2/Microscopy/EV2E_11.tif]

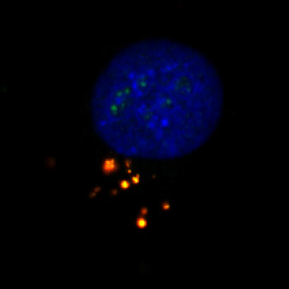

Supplement: Supplementary file 8 — Expanded View and Appendix Source Data [file 44318_2026_792_MOESM8_ESM.zip › Source data for Figure EV2/Microscopy/EV2E_12.tif]

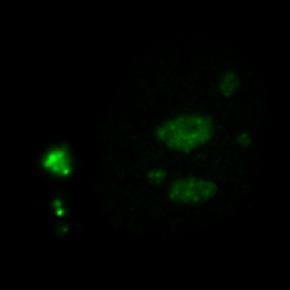

Supplement: Supplementary file 8 — Expanded View and Appendix Source Data [file 44318_2026_792_MOESM8_ESM.zip › Source data for Figure EV2/Microscopy/EV2E_13.tif]

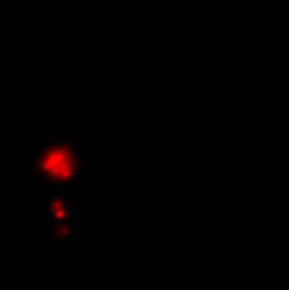

Supplement: Supplementary file 8 — Expanded View and Appendix Source Data [file 44318_2026_792_MOESM8_ESM.zip › Source data for Figure EV2/Microscopy/EV2E_14.tif]

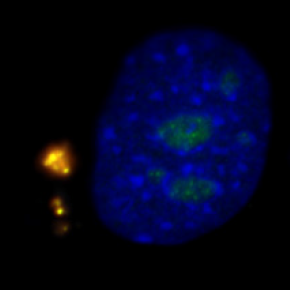

Supplement: Supplementary file 8 — Expanded View and Appendix Source Data [file 44318_2026_792_MOESM8_ESM.zip › Source data for Figure EV2/Microscopy/EV2E_15.tif]

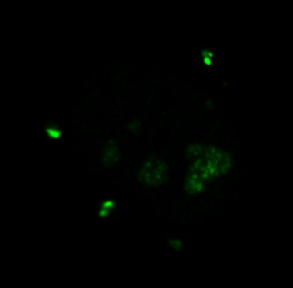

Supplement: Supplementary file 8 — Expanded View and Appendix Source Data [file 44318_2026_792_MOESM8_ESM.zip › Source data for Figure EV2/Microscopy/EV2E_16.tif]

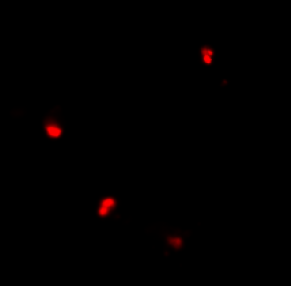

Supplement: Supplementary file 8 — Expanded View and Appendix Source Data [file 44318_2026_792_MOESM8_ESM.zip › Source data for Figure EV2/Microscopy/EV2E_17.tif]

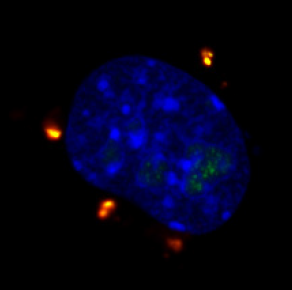

Supplement: Supplementary file 8 — Expanded View and Appendix Source Data [file 44318_2026_792_MOESM8_ESM.zip › Source data for Figure EV2/Microscopy/EV2E_18.tif]

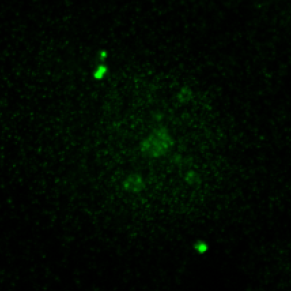

Supplement: Supplementary file 8 — Expanded View and Appendix Source Data [file 44318_2026_792_MOESM8_ESM.zip › Source data for Figure EV2/Microscopy/EV2E_19.tif]
