# Supplementary figures and images for: Cytosolic DNA inhibits rDNA transcription by retaining the RNA polymerase I transcription machinery (part 2 of 2)
Source: EMBO J. 2026 May 5;45(12):4153–75. doi: 10.1038/s44318-026-00792-2 (PMC13270134; doi:10.1038/s44318-026-00792-2)

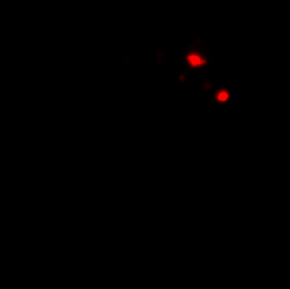

Supplement: Supplementary file 8 — Expanded View and Appendix Source Data [file 44318_2026_792_MOESM8_ESM.zip › Source data for Figure EV2/Microscopy/EV2E_2.tif]

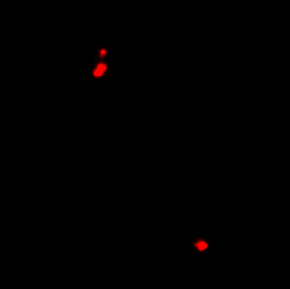

Supplement: Supplementary file 8 — Expanded View and Appendix Source Data [file 44318_2026_792_MOESM8_ESM.zip › Source data for Figure EV2/Microscopy/EV2E_20.tif]

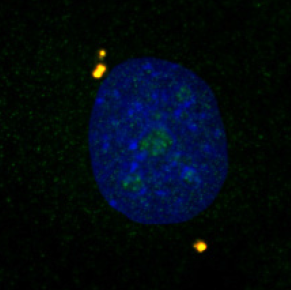

Supplement: Supplementary file 8 — Expanded View and Appendix Source Data [file 44318_2026_792_MOESM8_ESM.zip › Source data for Figure EV2/Microscopy/EV2E_21.tif]

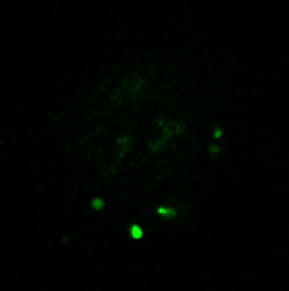

Supplement: Supplementary file 8 — Expanded View and Appendix Source Data [file 44318_2026_792_MOESM8_ESM.zip › Source data for Figure EV2/Microscopy/EV2E_22.tif]

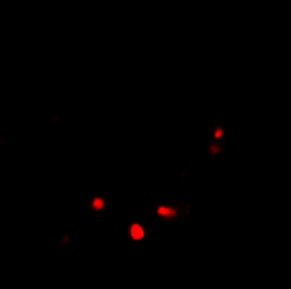

Supplement: Supplementary file 8 — Expanded View and Appendix Source Data [file 44318_2026_792_MOESM8_ESM.zip › Source data for Figure EV2/Microscopy/EV2E_23.tif]

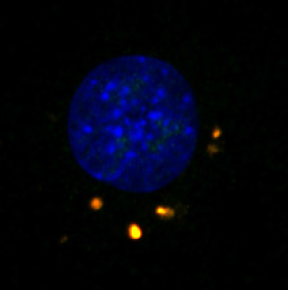

Supplement: Supplementary file 8 — Expanded View and Appendix Source Data [file 44318_2026_792_MOESM8_ESM.zip › Source data for Figure EV2/Microscopy/EV2E_24.tif]

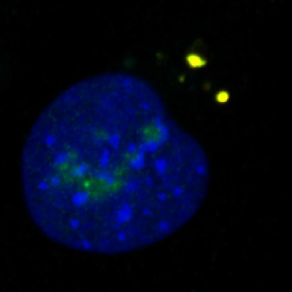

Supplement: Supplementary file 8 — Expanded View and Appendix Source Data [file 44318_2026_792_MOESM8_ESM.zip › Source data for Figure EV2/Microscopy/EV2E_3.tif]

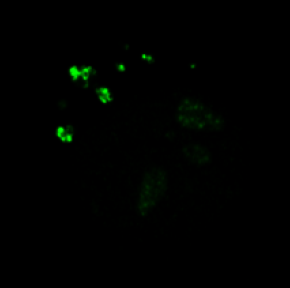

Supplement: Supplementary file 8 — Expanded View and Appendix Source Data [file 44318_2026_792_MOESM8_ESM.zip › Source data for Figure EV2/Microscopy/EV2E_4.tif]

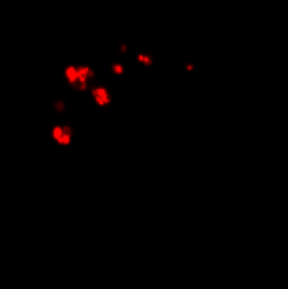

Supplement: Supplementary file 8 — Expanded View and Appendix Source Data [file 44318_2026_792_MOESM8_ESM.zip › Source data for Figure EV2/Microscopy/EV2E_5.tif]

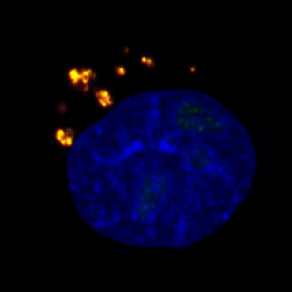

Supplement: Supplementary file 8 — Expanded View and Appendix Source Data [file 44318_2026_792_MOESM8_ESM.zip › Source data for Figure EV2/Microscopy/EV2E_6.tif]

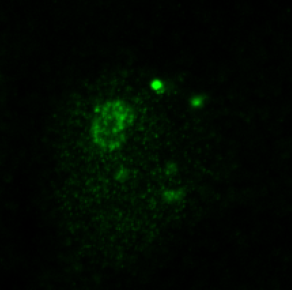

Supplement: Supplementary file 8 — Expanded View and Appendix Source Data [file 44318_2026_792_MOESM8_ESM.zip › Source data for Figure EV2/Microscopy/EV2E_7.tif]

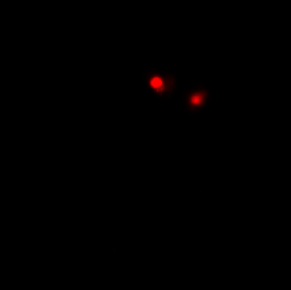

Supplement: Supplementary file 8 — Expanded View and Appendix Source Data [file 44318_2026_792_MOESM8_ESM.zip › Source data for Figure EV2/Microscopy/EV2E_8.tif]

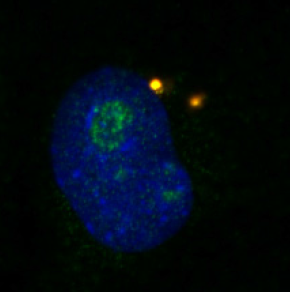

Supplement: Supplementary file 8 — Expanded View and Appendix Source Data [file 44318_2026_792_MOESM8_ESM.zip › Source data for Figure EV2/Microscopy/EV2E_9.tif]

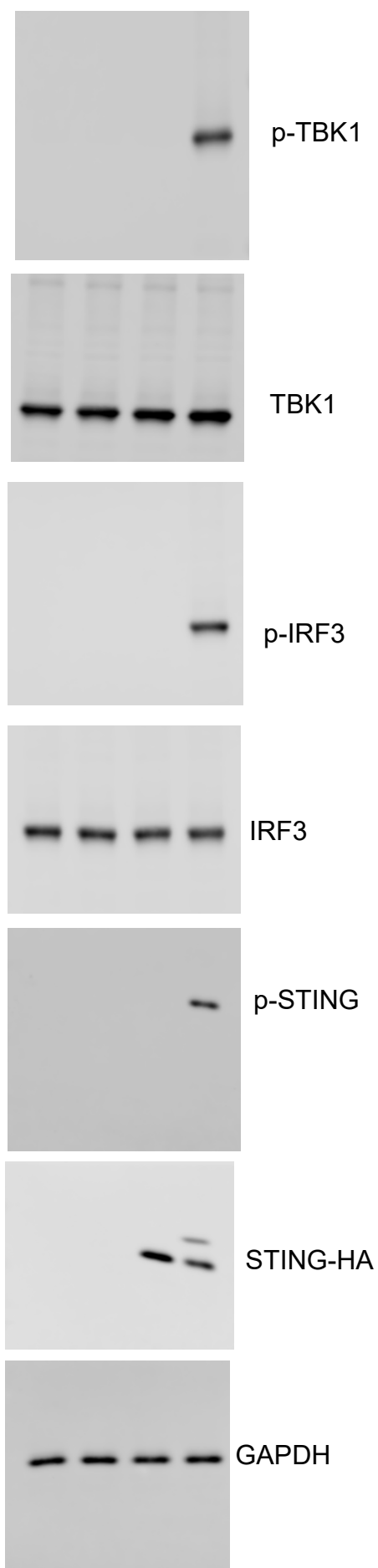

**Fig EV3A**

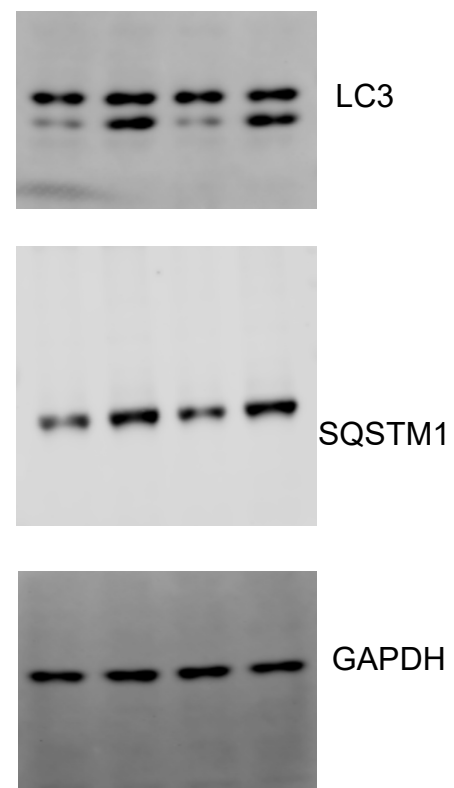

**Fig EV3D**

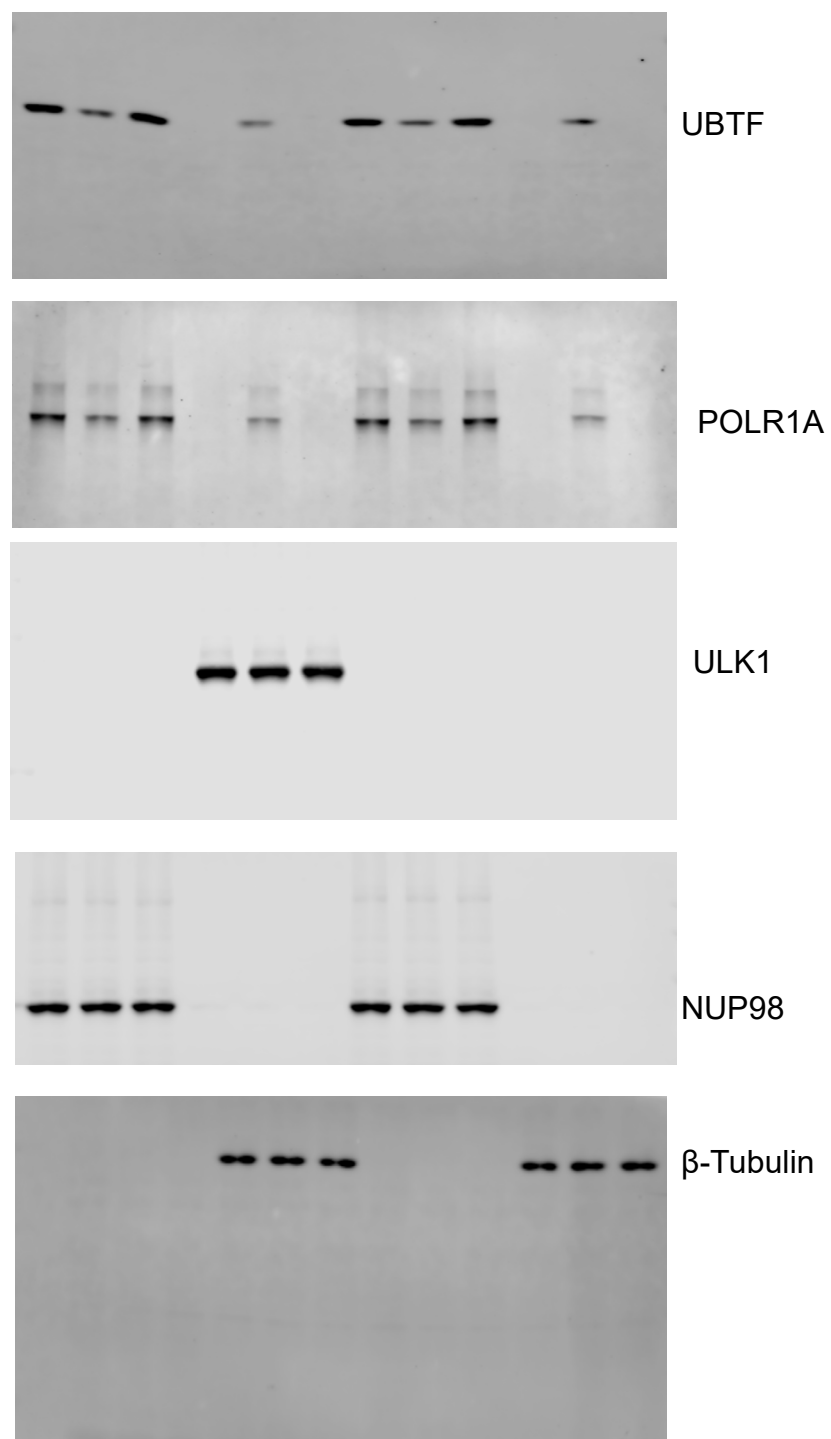

**Fig EV3F**

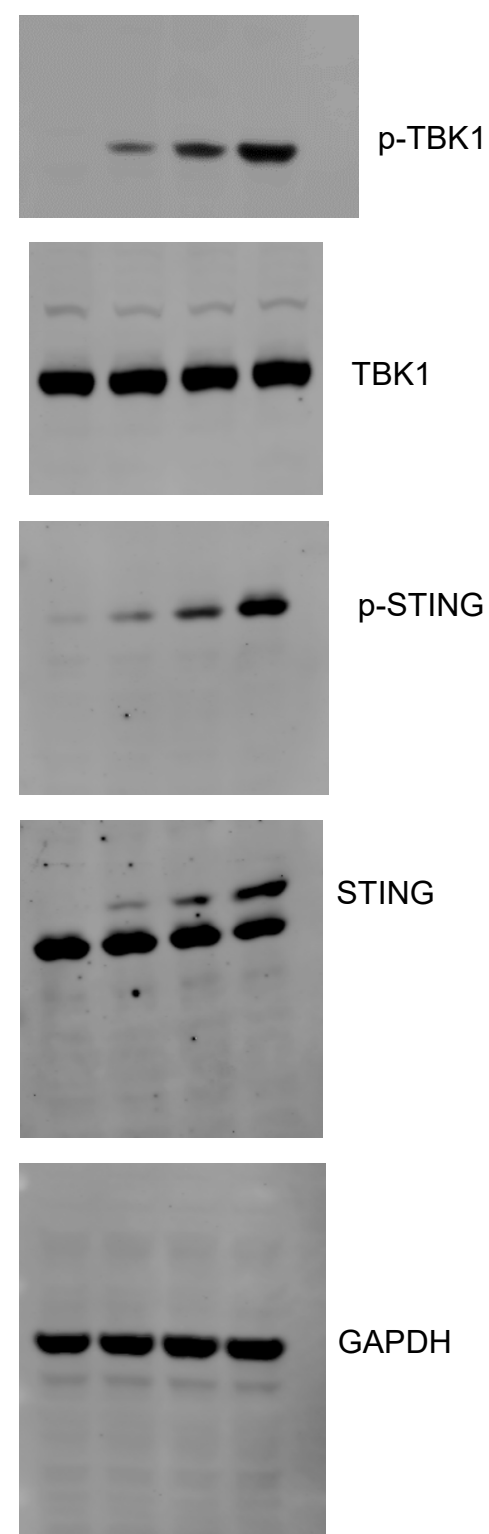

**Fig EV3G**

Supplement: Supplementary file 8 — Expanded View and Appendix Source Data [file 44318_2026_792_MOESM8_ESM.zip › Source data for Figure EV3/Gel data/EV3A, D, F, and G.pdf]

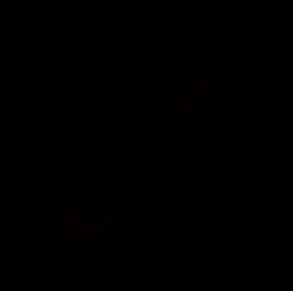

Supplement: Supplementary file 8 — Expanded View and Appendix Source Data [file 44318_2026_792_MOESM8_ESM.zip › Source data for Figure EV3/Microscopy/EV3B/EV3B_1.tif]

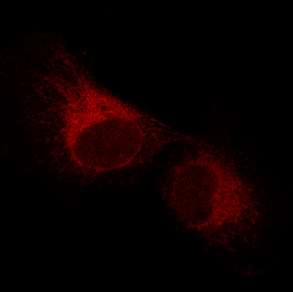

Supplement: Supplementary file 8 — Expanded View and Appendix Source Data [file 44318_2026_792_MOESM8_ESM.zip › Source data for Figure EV3/Microscopy/EV3B/EV3B_10.tif]

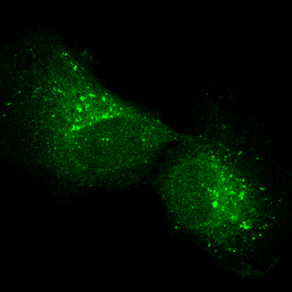

Supplement: Supplementary file 8 — Expanded View and Appendix Source Data [file 44318_2026_792_MOESM8_ESM.zip › Source data for Figure EV3/Microscopy/EV3B/EV3B_11.tif]

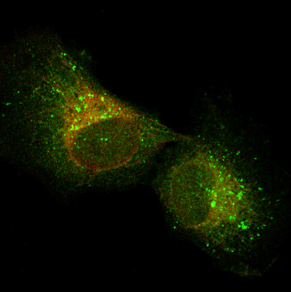

Supplement: Supplementary file 8 — Expanded View and Appendix Source Data [file 44318_2026_792_MOESM8_ESM.zip › Source data for Figure EV3/Microscopy/EV3B/EV3B_12.tif]

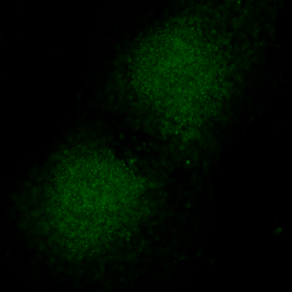

Supplement: Supplementary file 8 — Expanded View and Appendix Source Data [file 44318_2026_792_MOESM8_ESM.zip › Source data for Figure EV3/Microscopy/EV3B/EV3B_2.tif]

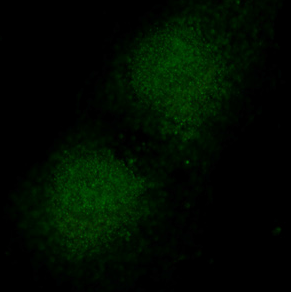

Supplement: Supplementary file 8 — Expanded View and Appendix Source Data [file 44318_2026_792_MOESM8_ESM.zip › Source data for Figure EV3/Microscopy/EV3B/EV3B_3.tif]

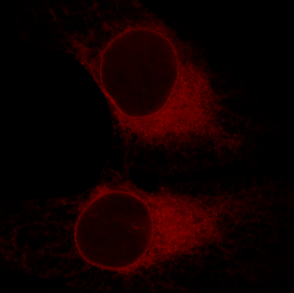

Supplement: Supplementary file 8 — Expanded View and Appendix Source Data [file 44318_2026_792_MOESM8_ESM.zip › Source data for Figure EV3/Microscopy/EV3B/EV3B_4.tif]

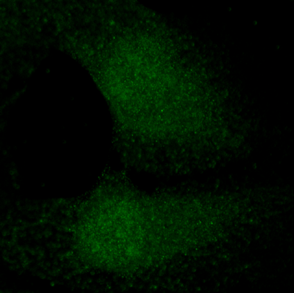

Supplement: Supplementary file 8 — Expanded View and Appendix Source Data [file 44318_2026_792_MOESM8_ESM.zip › Source data for Figure EV3/Microscopy/EV3B/EV3B_5.tif]

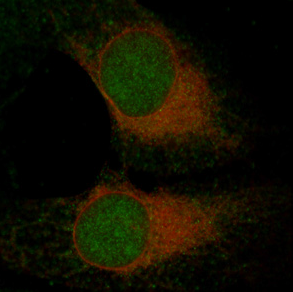

Supplement: Supplementary file 8 — Expanded View and Appendix Source Data [file 44318_2026_792_MOESM8_ESM.zip › Source data for Figure EV3/Microscopy/EV3B/EV3B_6.tif]

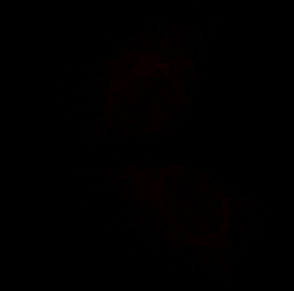

Supplement: Supplementary file 8 — Expanded View and Appendix Source Data [file 44318_2026_792_MOESM8_ESM.zip › Source data for Figure EV3/Microscopy/EV3B/EV3B_7.tif]

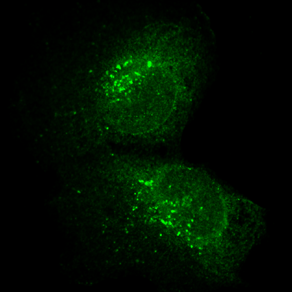

Supplement: Supplementary file 8 — Expanded View and Appendix Source Data [file 44318_2026_792_MOESM8_ESM.zip › Source data for Figure EV3/Microscopy/EV3B/EV3B_8.tif]

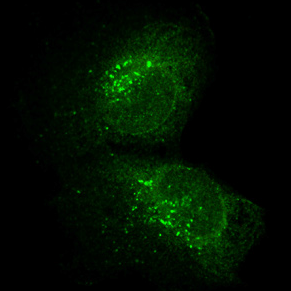

Supplement: Supplementary file 8 — Expanded View and Appendix Source Data [file 44318_2026_792_MOESM8_ESM.zip › Source data for Figure EV3/Microscopy/EV3B/EV3B_9.tif]

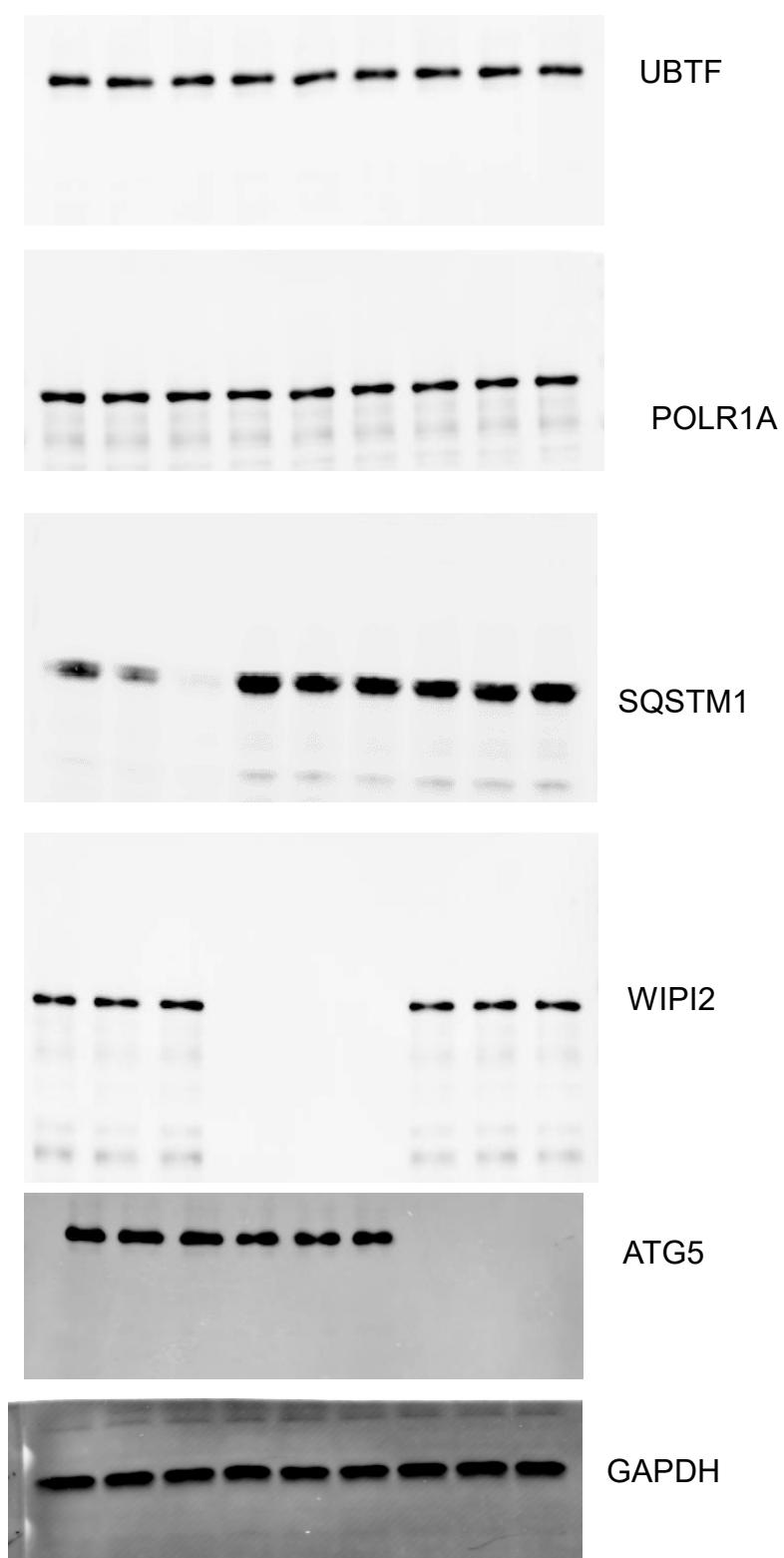

**Fig EV4A**

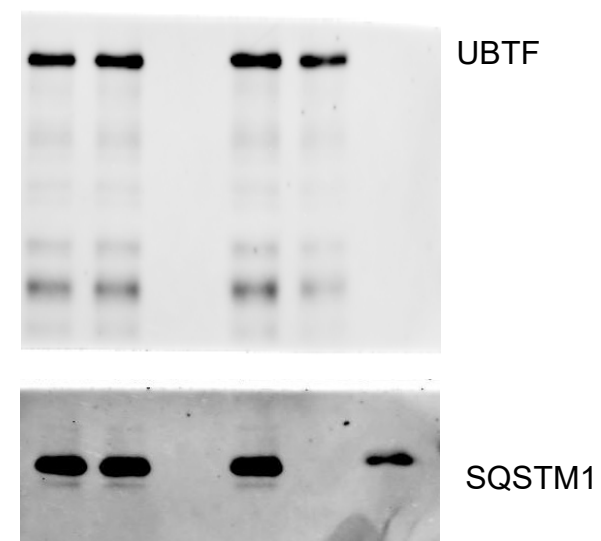

**Fig EV4C**

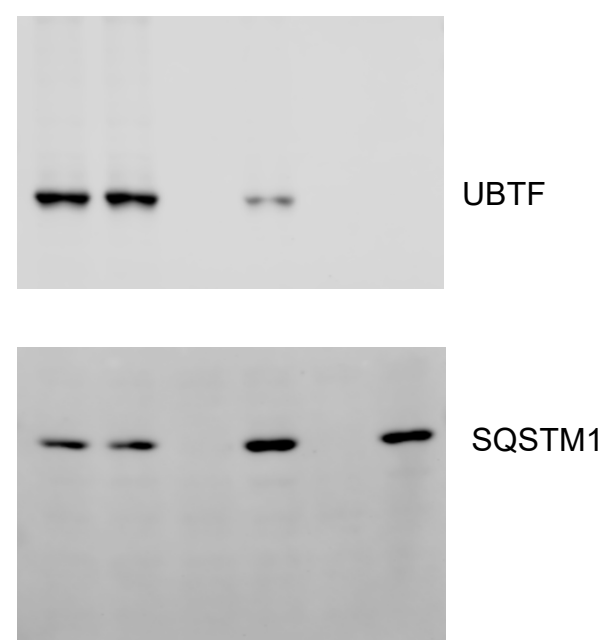

**Fig EV4D**

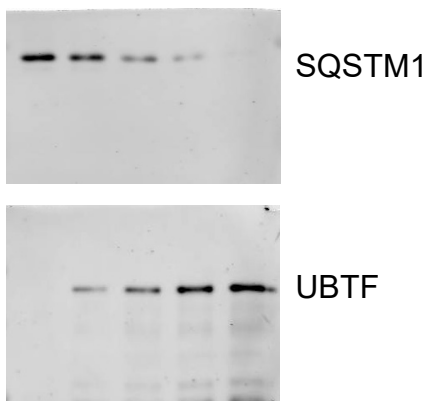

**Fig EV4F**

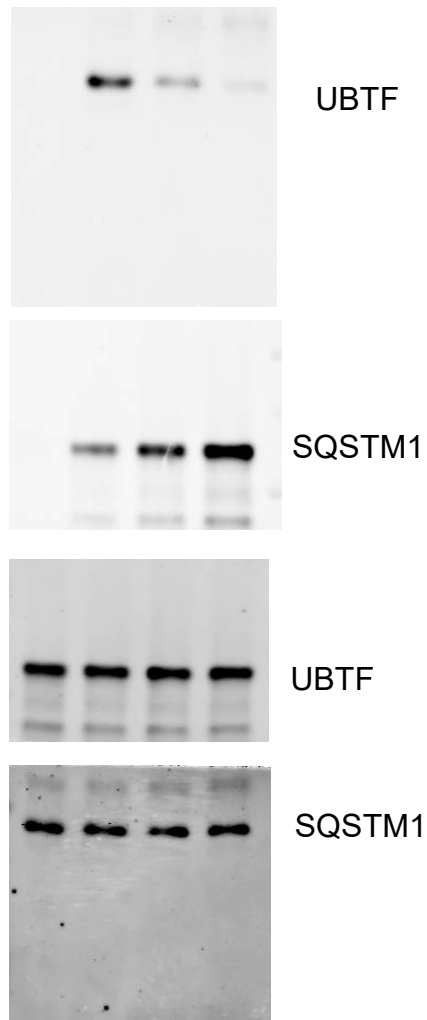

**Fig EV4G**

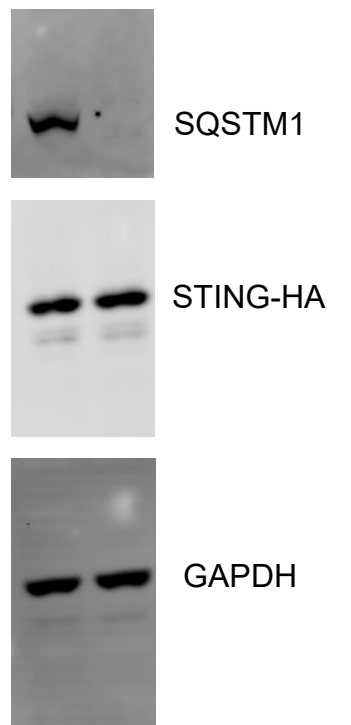

**Fig EV4J**

Supplement: Supplementary file 8 — Expanded View and Appendix Source Data [file 44318_2026_792_MOESM8_ESM.zip › Source data for Figure EV4/Gel data/EV4A, C, D, F, G, and J.pdf]

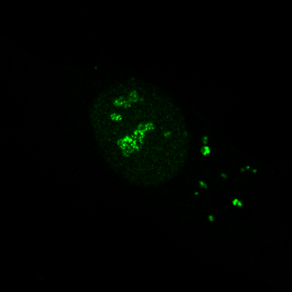

Supplement: Supplementary file 8 — Expanded View and Appendix Source Data [file 44318_2026_792_MOESM8_ESM.zip › Source data for Figure EV4/Microscopy/EV4H/EV4H_1.tif]

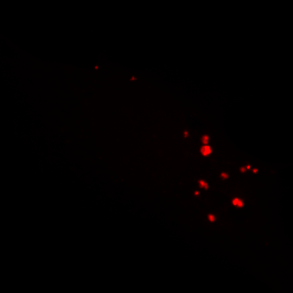

Supplement: Supplementary file 8 — Expanded View and Appendix Source Data [file 44318_2026_792_MOESM8_ESM.zip › Source data for Figure EV4/Microscopy/EV4H/EV4H_2.tif]

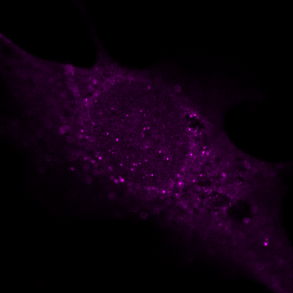

Supplement: Supplementary file 8 — Expanded View and Appendix Source Data [file 44318_2026_792_MOESM8_ESM.zip › Source data for Figure EV4/Microscopy/EV4H/EV4H_3.tif]

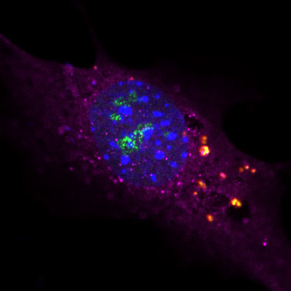

Supplement: Supplementary file 8 — Expanded View and Appendix Source Data [file 44318_2026_792_MOESM8_ESM.zip › Source data for Figure EV4/Microscopy/EV4H/EV4H_4.tif]

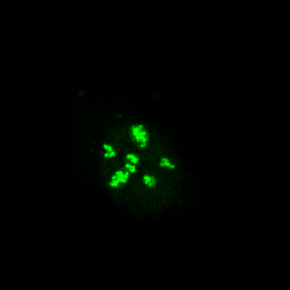

Supplement: Supplementary file 8 — Expanded View and Appendix Source Data [file 44318_2026_792_MOESM8_ESM.zip › Source data for Figure EV4/Microscopy/EV4H/EV4H_5.tif]

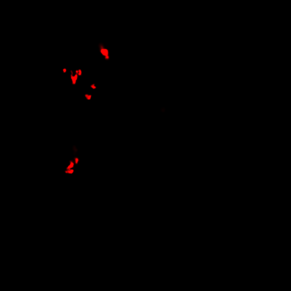

Supplement: Supplementary file 8 — Expanded View and Appendix Source Data [file 44318_2026_792_MOESM8_ESM.zip › Source data for Figure EV4/Microscopy/EV4H/EV4H_6.tif]

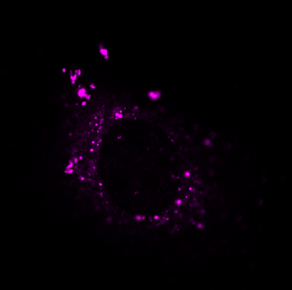

Supplement: Supplementary file 8 — Expanded View and Appendix Source Data [file 44318_2026_792_MOESM8_ESM.zip › Source data for Figure EV4/Microscopy/EV4H/EV4H_7.tif]

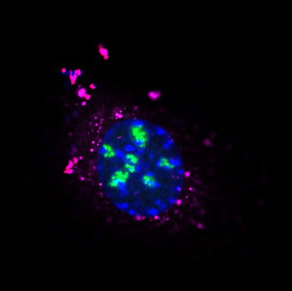

Supplement: Supplementary file 8 — Expanded View and Appendix Source Data [file 44318_2026_792_MOESM8_ESM.zip › Source data for Figure EV4/Microscopy/EV4H/EV4H_8.tif]

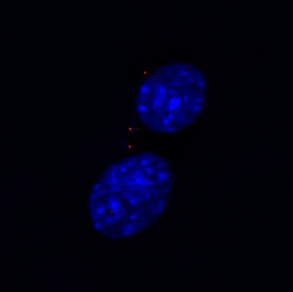

Supplement: Supplementary file 8 — Expanded View and Appendix Source Data [file 44318_2026_792_MOESM8_ESM.zip › Source data for Figure EV4/Microscopy/EV4K/EV4K_1.tif]

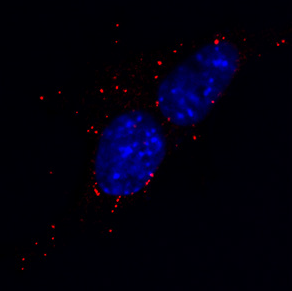

Supplement: Supplementary file 8 — Expanded View and Appendix Source Data [file 44318_2026_792_MOESM8_ESM.zip › Source data for Figure EV4/Microscopy/EV4K/EV4K_2.tif]

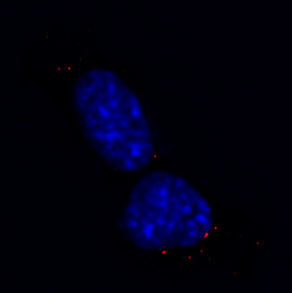

Supplement: Supplementary file 8 — Expanded View and Appendix Source Data [file 44318_2026_792_MOESM8_ESM.zip › Source data for Figure EV4/Microscopy/EV4K/EV4K_3.tif]

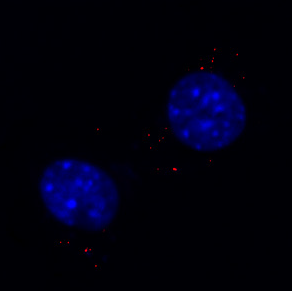

Supplement: Supplementary file 8 — Expanded View and Appendix Source Data [file 44318_2026_792_MOESM8_ESM.zip › Source data for Figure EV4/Microscopy/EV4K/EV4K_4.tif]

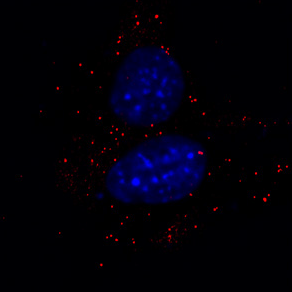

Supplement: Supplementary file 8 — Expanded View and Appendix Source Data [file 44318_2026_792_MOESM8_ESM.zip › Source data for Figure EV4/Microscopy/EV4K/EV4K_5.tif]

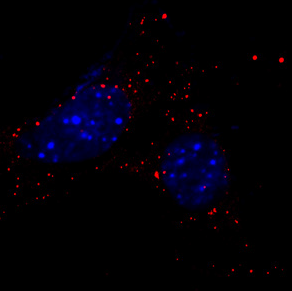

Supplement: Supplementary file 8 — Expanded View and Appendix Source Data [file 44318_2026_792_MOESM8_ESM.zip › Source data for Figure EV4/Microscopy/EV4K/EV4K_6.tif]
