# Supplementary material for: Cytosolic DNA inhibits rDNA transcription by retaining the RNA polymerase I transcription machinery
Source: EMBO J. 2026 May 5;45(12):4153–75. doi: 10.1038/s44318-026-00792-2 (PMC13270134; doi:10.1038/s44318-026-00792-2)
Supplement: Supplementary file 9 — Expanded View Figures [file 44318_2026_792_MOESM9_ESM.pdf]

Expanded View Figures

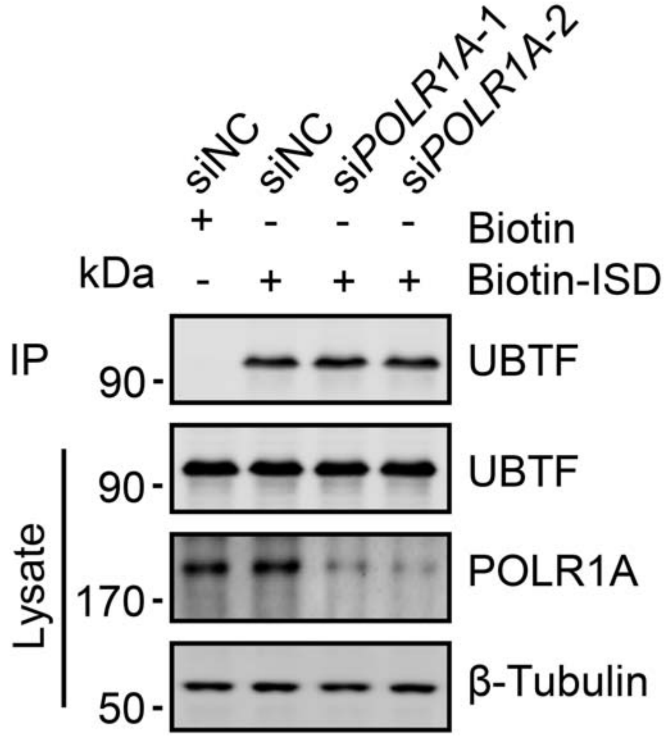

Figure EV1. POLR1A is not required for the interaction between UBTF and ISD.

Cells were treated with or without *POLR1A* siRNAs and then transfected with biotin-ISD. Following that, biotin-ISD was pulled down and the bound proteins were analyzed by western blot using anti-UBTF. Source data are available online for this figure.

 © The Author(s)

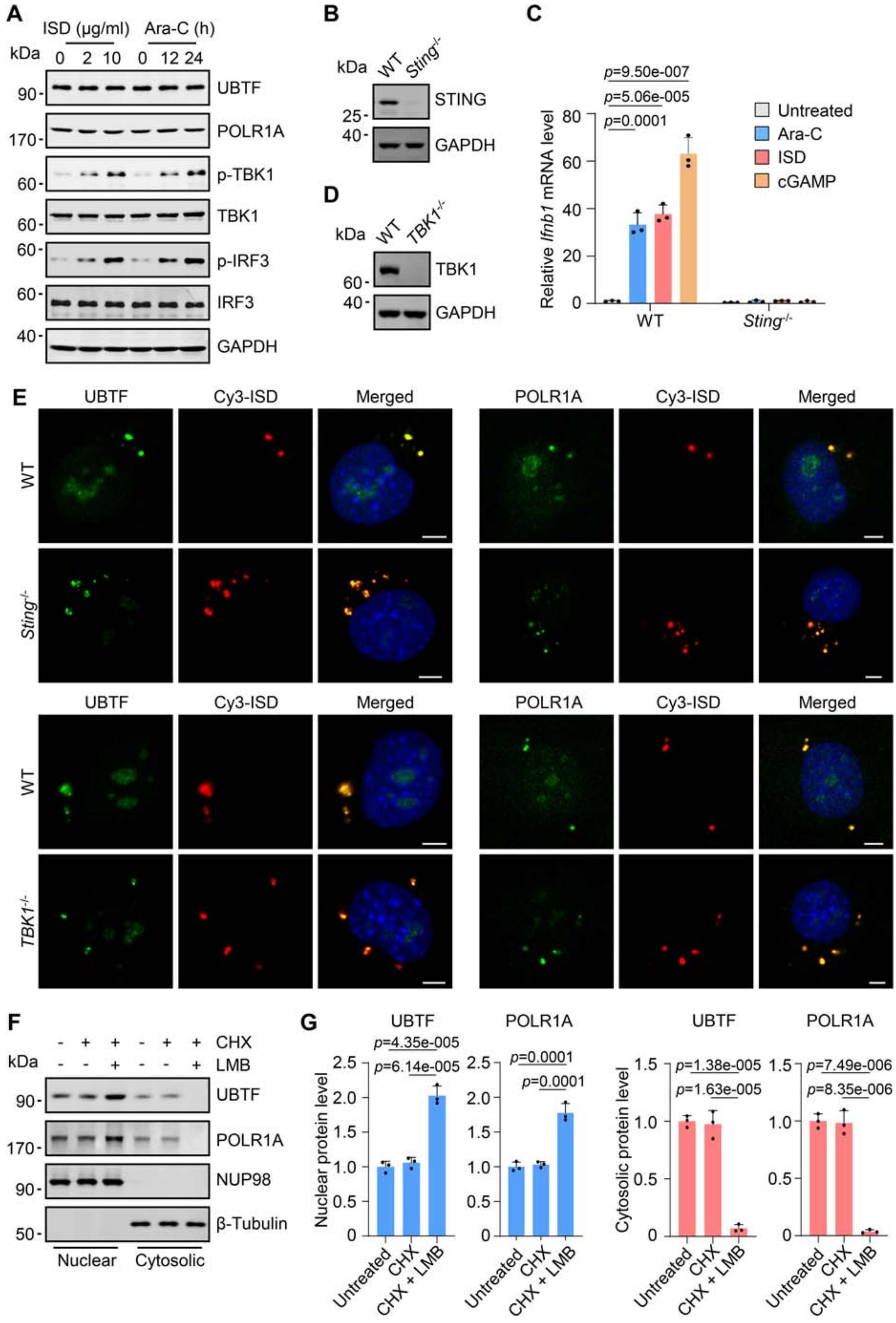
A
ISD (μg/ml) Ara-C (h)
kDa 0 2 10 0 12 24
UBTF
POLR1A
p-TBK1
TBK1
p-IRF3
IRF3
GAPDH
B
kDa WT Sting-/-
STING
GAPDH
C
p=9.50e-007
p=5.06e-005
p=0.0001
Relative Ifnb1 mRNA level
Untreated
Ara-C
ISD
cGAMP
WT Sting-/-
D
kDa WT TBK1-/-
TBK1
GAPDH
E
UBTF Cy3-ISD Merged POLR1A Cy3-ISD Merged
WT
Sting-/-
TBK1-/-
F
CHX
LMB
UBTF
POLR1A
NUP98
β-Tubulin
Nuclear Cytosolic
G
UBTF POLR1A UBTF POLR1A
p=4.35e-005
p=6.14e-005
p=0.0001
p=0.0001
p=1.38e-005
p=1.63e-005
p=7.49e-006
p=8.35e-006
Nuclear protein level
Cytosolic protein level
Untreated CHX CHX + LMB

Figure EV2. The cGAS-STING signaling is not required for the retention of UBTF and POLR1A in the cytoplasm by cytosolic DNA.

(**A**) The protein levels of UBTF and POLR1A in MEFs transfected with ISD or treated with arabinofuranosyl cytidine (Ara-C) as indicated. (**B**) Verification of *Sting* knockout MEFs. (**C**) Statistical analysis of *Ifnb1* mRNA levels in wild-type (WT) and *Sting*$^{-/-}$ MEFs. The cells were treated as indicated. (**D**) Verification of *TBK1* knockout DLD1 cells. (**E**) Subcellular localization of UBTF and POLR1A in cells transfected with Cy3 labeled ISD (Cy3-ISD). WT and *Sting*$^{-/-}$ MEFs, or WT and *TBK1*$^{-/-}$ DLD1 cells, were transfected with Cy3-ISD and subjected to immunostaining using anti-UBTF and anti-POLR1A, respectively. Scale bars, 10 µm. (**F**) The protein levels of UBTF and POLR1A in the nuclear and cytoplasmic fractions of MEFs. Cells were incubated with cycloheximide (CHX), a protein synthesis inhibitor, or co-treated with leptomycin B (LMB), a nuclear export inhibitor of proteins. Following that, the cells were transfected with Cy3-ISD and subjected to cell fractionation experiments. (**G**) Statistical analysis of the protein levels of UBTF and POLR1A in the nuclear and cytoplasmic fractions from cells treated as in (**F**). The relative levels of nuclear UBTF and POLR1A were normalized to NUP98, and the relative levels of cytoplasmic UBTF and POLR1A were normalized to β-Tubulin. All statistical data are presented as mean ± SD of three independent experiments and analyzed by one-way ANOVA and Tukey's post hoc test. Source data are available online for this figure.

 © The Author(s)

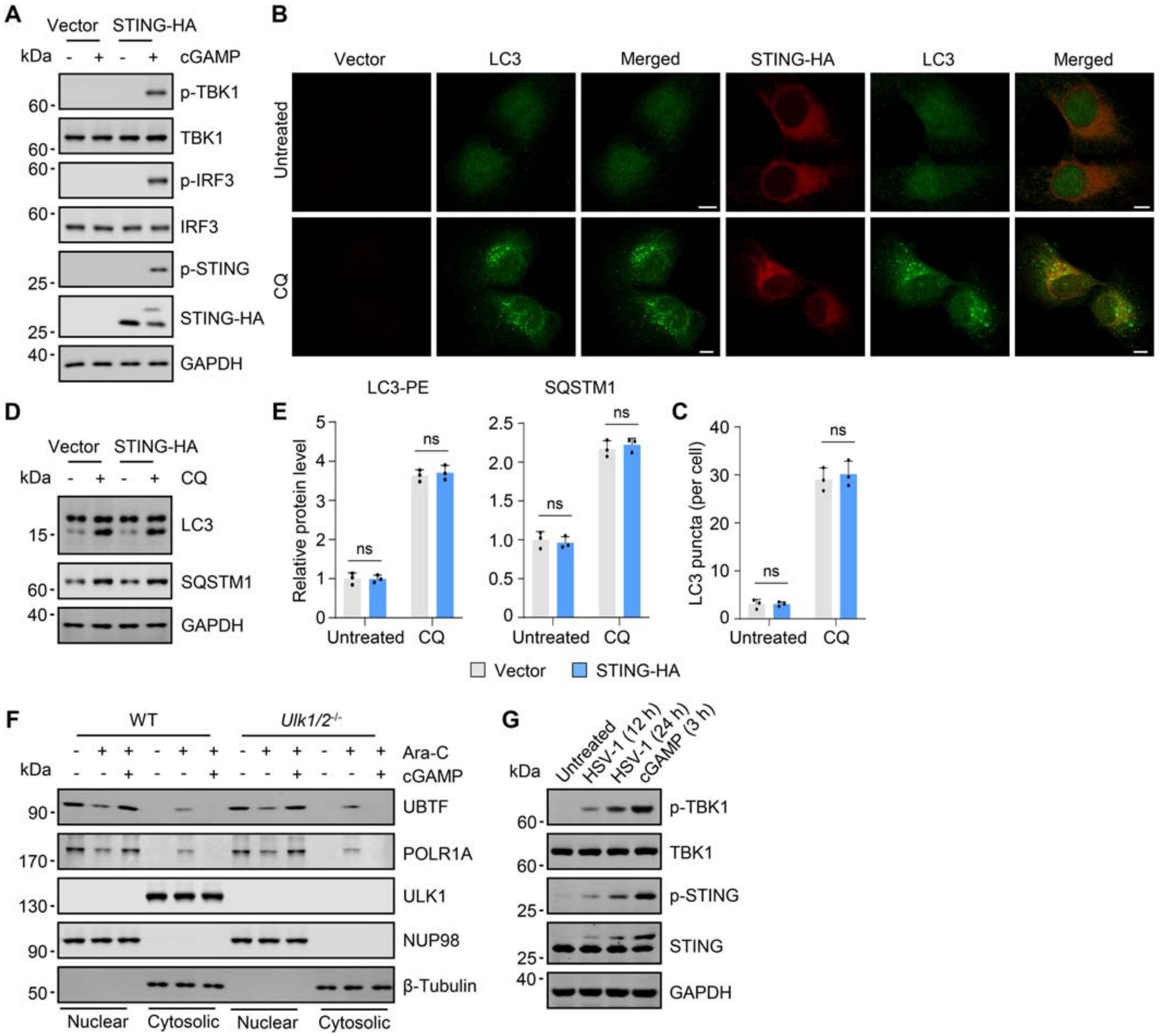

Figure EV3. STING does not affect basal autophagy.

(**A**) Verification of the functionality of STING in HEK293T cells reconstituted with HA-tagged STING. The cells were treated with or without cGAMP and subjected to western blot analysis using anti-phospho-TBK1 (Ser172), anti-phospho-IRF3 (Ser396), and anti-phospho-STING (Ser366). (**B**) LC3 punctum formation in HEK293T cells stably expressing STING-HA. The cells were treated with or without chloroquine (CQ), a lysosome inhibitor, and subjected to immunostaining using anti-LC3. Scale bars, 10 μm. (**C**) Statistical analysis of the number of LC3 puncta in cells treated as in (**B**). 60 cells were analyzed from three independent experiments. (**D**) The protein levels of lipidated LC3 and SQSTM1 in HEK293T cells stably expressing STING-HA. The cells were treated with or without CQ. (**E**) Statistical analysis of the protein levels of lipidated LC3 and SQSTM1 in cells treated as in (**D**). (**F**) The protein levels of UBTF and POLR1A in the nuclear and cytoplasmic fractions of cells. Wild-type (WT) and *Ulk1/2*$^{-/-}$ MEFs were treated with Ara-C for 12 h and then stimulated with or without cGAMP for another 12 h. (**G**) Activation of STING by HSV-1 infection. MEFs were infected with HSV-1 or treated with cGAMP, then subjected to western blot analysis using anti-phospho-TBK1 (Ser172) and anti-phospho-STING (Ser366). All statistical data are presented as mean ± SD of three independent experiments and analyzed by the unpaired two-tailed Student's *t* test. ns, not significant. Source data are available online for this figure.

© The Author(s)

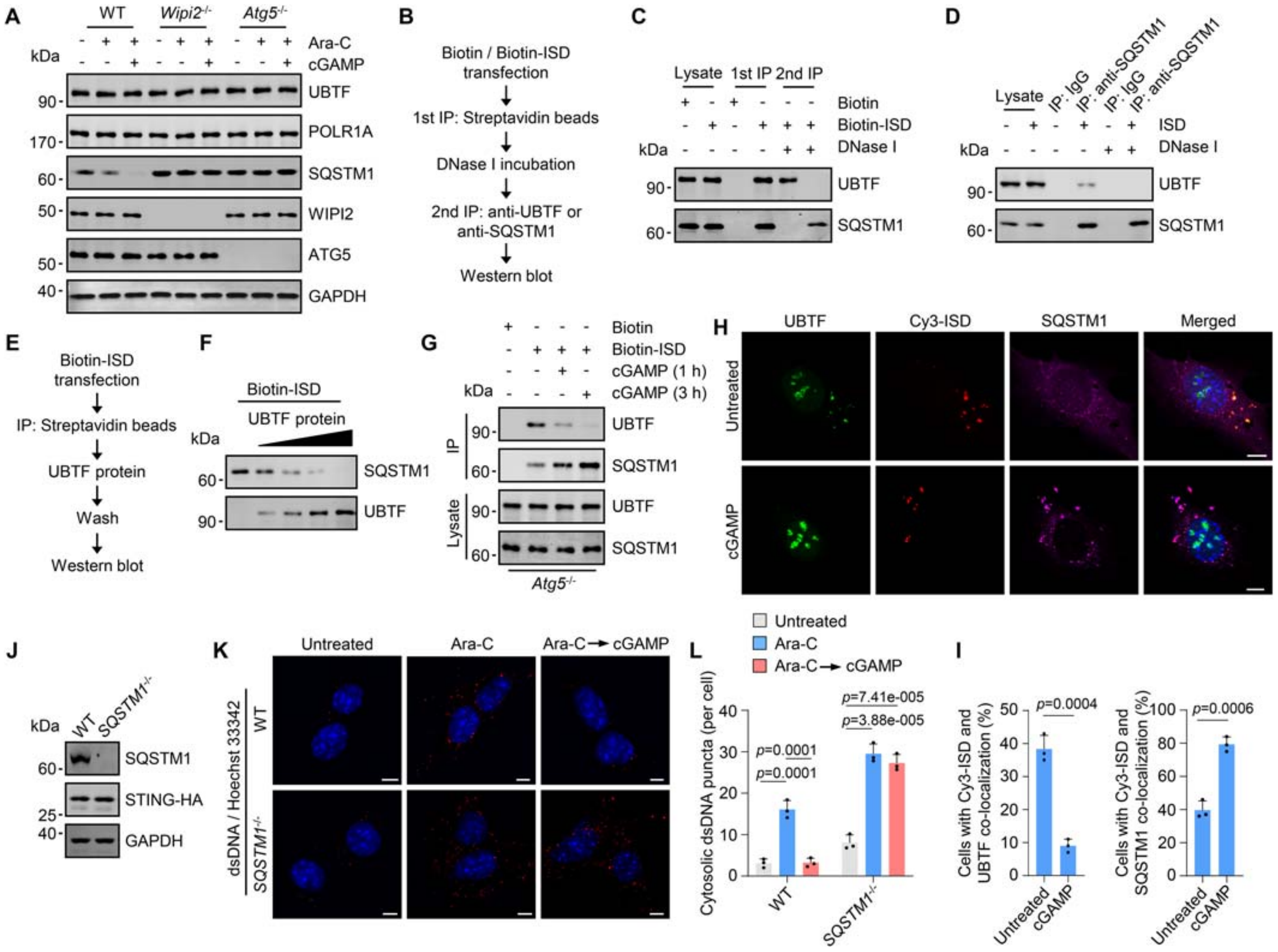

Figure EV4. UBTF and SQSTM1 compete for binding to cytosolic DNA.

(**A**) The protein levels of UBTF, POLR1A, and SQSTM1 in wild-type (WT), *Wipi2*$^{-/-}$, and *Atg5*$^{-/-}$ MEFs. The cells were treated with arabinofuranosyl cytidine (Ara-C) for 12 h and then cultured in fresh medium and incubated with or without cGAMP for another 12 h. The cell lysates from these cells were subjected to western blot analysis using anti-UBTF, anti-POLR1A, and anti-SQSTM1. (**B**) Schematic of the workflow for the two-step co-immunoprecipitation experiment. (**C**) MEFs transfected with biotin-ISD were lysed and incubated with streptavidin beads. After the incubation, the precipitates were treated with DNase I, the eluates were then subjected to the immunoprecipitation experiments with either anti-UBTF or anti-SQSTM1. The resulting immunoprecipitates were analyzed by western blot using anti-SQSTM1 and anti-UBTF, respectively. (**D**) MEFs transfected with ISD were lysed and incubated with or without DNase I. After the incubation, the cell lysates were subjected to the immunoprecipitation experiments using anti-SQSTM1. The resulting immunoprecipitates were analyzed by western blot using anti-UBTF. (**E**) Schematic of the workflow for examining the competitive binding of UBTF and SQSTM1 to biotin-ISD. (**F**) MEFs transfected with biotin-ISD were lysed and pulled down by streptavidin beads. After that, the precipitates were incubated with different amounts of recombinant UBTF protein, and then washed, and subjected to western blot analysis using anti-UBTF and anti-SQSTM1. (**G**) Association of endogenous UBTF and SQSTM1 with biotin-ISD in *Atg5*$^{-/-}$ MEFs. The cells transfected with biotin-ISD were stimulated with cGAMP for 1 or 3 h. Biotin-ISD was then pulled down from these cells and the precipitates were subjected to western blot analysis using anti-UBTF and anti-SQSTM1. (**H**) Subcellular localization of UBTF and SQSTM1 in MEFs. The cells were transfected with Cy3-ISD and stimulated with or without cGAMP. After the treatments, the cells were stained using anti-UBTF and anti-SQSTM1. (**I**) Statistical analysis of the co-localization between Cy3-ISD and UBTF, as well as between Cy3-ISD and SQSTM1, in cells treated as in (**H**). (**J**) Verification of WT and *SQSTM1*$^{-/-}$ HEK293 cells stably expressing HA-tagged STING. (**K**) Endogenous cytosolic DNA in cells. WT and *SQSTM1*$^{-/-}$ cells stably expressing HA-tagged STING were treated with Ara-C for 12 h and then cultured in fresh medium and incubated with or without cGAMP for another 12 h. (**L**) Statistical analysis of cytosolic DNA puncta in cells treated as in (**K**). In total, 60 cells were analyzed from three independent experiments. All statistical data are presented as mean ± SD of three independent experiments. The unpaired two-tailed Student's *t* test performed for (**I**), and one-way ANOVA and Tukey's post hoc test performed for (**L**). Scale bars, 10 μm. Source data are available online for this figure.

 © The Author(s)

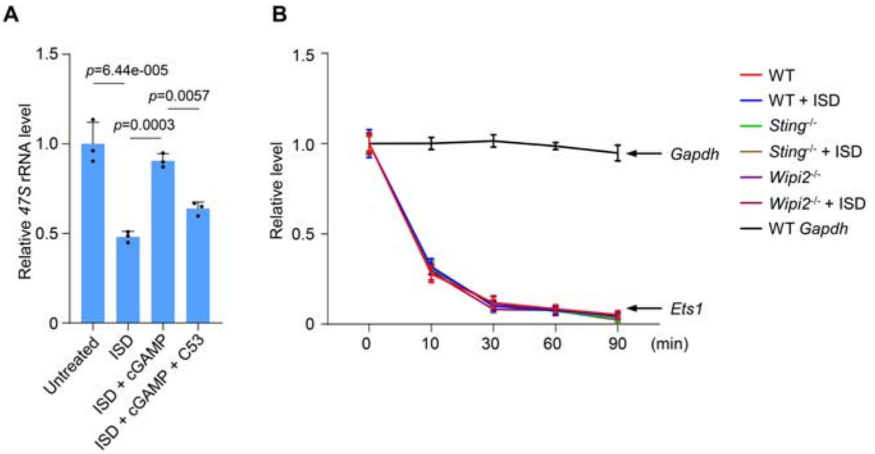

Figure EV5. STING-induced autophagy does not affect *47S* rRNA degradation.

(**A**) Cellular *47S* rRNA level in DLD1 cells. The cells were transfected with ISD and treated as indicated. *47S* rRNA level of the cells was measured by real-time PCR and normalized to *ACTB* mRNA. cGAMP, a STING agonist; compound 53 (C53), a STING agonist that can block its proton channel activity. (**B**) Cellular *Ets1* and *Gapdh* mRNA levels in wild-type (WT), *Sting*$^{-/-}$, and *Wipi2*$^{-/-}$ MEFs. The cells were transfected with or without ISD and incubated with 20 μg/ml actinomycin D (Act-D). The *Ets1* and *Gapdh* mRNA levels were measured by real-time PCR and normalized to *Actb* mRNA. *Gaphd* mRNA level was used as the internal control. Act-D, a transcription inhibitor. All statistical data are presented as mean ± SD of three independent experiments and analyzed by one-way ANOVA and Tukey's post hoc test. Source data are available online for this figure.
