# Supplementary material for: Cytosolic DNA inhibits rDNA transcription by retaining the RNA polymerase I transcription machinery
Source: EMBO J. 2026 May 5;45(12):4153–75. doi: 10.1038/s44318-026-00792-2 (PMC13270134; doi:10.1038/s44318-026-00792-2)
Supplement: Supplementary file 2 — Peer Review File [file 44318_2026_792_MOESM2_ESM.pdf]

THE EMBO JOURNAL

Cytosolic DNA inhibits rDNA transcription by retaining the RNA polymerase I transcription machinery

Yinfeng Xu, Qian Wang, Chuying Qian, Sheng Lu, Zhengfu He, Wei Liu, and Wei Wan

Corresponding author(s): Wei Wan (wanwei@zju.edu.cn) , Wei Liu (liuwei666@zju.edu.cn), Wei Wan (wanwei@zju.edu.cn), Yinfeng Xu (yinfengxu@hnfnu.edu.cn)

Review Timeline:		
	Submission Date:	26th Jun 25
	Editorial Decision:	29th Sep 25
	Revision Received:	27th Dec 25
	Editorial Decision:	2nd Feb 26
	Revision Received:	8th Feb 26
	Editorial Decision:	27th Feb 26
	Revision Received:	12th Mar 26
	Accepted:	7th Apr 26

Editor: Cornelius Schneider

Transaction Report:

(Note: With the exception of the correction of typographical or spelling errors that could be a source of ambiguity, letters and reports are not edited. Depending on transfer agreements, referee reports obtained elsewhere may or may not be included in this compilation. Referee reports are anonymous unless the Referee chooses to sign their reports.)

Dear Prof. Wan,

Thank you for submitting your manuscript for consideration by the EMBO Journal. It has now been seen by three referees whose comments are shown below.

As you can see for the referee comments all three referees think that the findings are interesting but also voice several concerns both regarding the depth of analysis and also think that several claims would need additional support. Given the referees' positive recommendations, I would like to invite you to submit a revised version of the manuscript, addressing the comments of all three reviewers. In particular we require that the concerns raised by referee #1 regarding the physiological relevance of the findings has to be addressed convincingly. I should add that it is EMBO Journal policy to allow only a single round of revision, and acceptance of your manuscript will therefore depend on the completeness of your responses in this revised version.

When preparing your letter of response to the referees' comments, please bear in mind that this will form part of the Review Process File, and will therefore be available online to the community. For more details on our Transparent Editorial Process, please visit our website: https://www.embopress.org/page/journal/14602075/authorguide#transparentprocess

We generally allow three months as standard revision time. As a matter of policy, competing manuscripts published during this period will not negatively impact on our assessment of the conceptual advance presented by your study. However, we request that you contact the editor as soon as possible upon publication of any related work, to discuss how to proceed. Should you foresee a problem in meeting this three-month deadline, please let us know in advance and we may be able to grant an extension.

Thank you for the opportunity to consider your work for publication. I look forward to your revision.

Yours sincerely,

Cornelius Schneider, PhD
Editor
The EMBO Journal
c.schneider@embojournal.org

Instructions for preparing your revised manuscript:

Please make sure you upload a letter of response to the referees' comments together with the revised manuscript.

Please also check that the title and abstract of the manuscript are brief, yet explicit, even to non-specialists.

When assembling figures, please refer to our figure preparation guideline in order to ensure proper formatting and readability in print as well as on screen:
https://bit.ly/EMBOPressFigurePreparationGuideline
See also guidelines for figure legends: https://www.embopress.org/page/journal/14602075/authorguide#figureformat

At EMBO Press we ask authors to provide source data for the main manuscript figures. You will receive a separate email with instructions for providing source data with your revised manuscript, including how to upload and organize the files.

IMPORTANT: When you send the revision we will require
- a point-by-point response to the referees' comments, with a detailed description of the changes made (as a word file).
- a word file of the manuscript text.
- individual production quality figure files (one file per figure)
- a complete author checklist, which you can download from our author guidelines (https://www.embopress.org/page/journal/14602075/authorguide).
- Expanded View files (replacing Supplementary Information)
Please see out instructions to authors
https://www.embopress.org/page/journal/14602075/authorguide#expandedview
- a Reagents and Tools Table as part of the Methods section, which can be downloaded from our author guidelines (https://www.embopress.org/page/journal/14602075/authorguide#structuredmethods)

Please remember: Digital image enhancement is acceptable practice, as long as it accurately represents the original data and

conforms to community standards. If a figure has been subjected to significant electronic manipulation, this must be noted in the figure legend or in the 'Materials and Methods' section. The editors reserve the right to request original versions of figures and the original images that were used to assemble the figure.

Further information is available in our Guide For Authors: https://www.embopress.org/page/journal/14602075/authorguide

We realize that it is difficult to revise to a specific deadline. In the interest of protecting the conceptual advance provided by the work, we recommend a revision within 3 months (28th Dec 2025). Please discuss the revision progress ahead of this time with the editor if you require more time to complete the revisions. Use the link below to submit your revision:

https://emboj.msubmit.net/cgi-bin/main.plex

Referee #1:

In this manuscript, the authors proposed that cytosolic DNA retains UBTF and POLR1A in the cytosol, thereby inhibiting rDNA transcription that occurs in the nucleolus. Their findings are potentially interesting, however, many issues need to be cleared. What is critically missing in the study is demonstration of the biological/pathological significance of the relocation of UBTF and POLR1A to the cytosol. The authors routinely use ISD transfection and/or Ara-C, surely producing the tons of cytosolic DNA, which never occurs in physiological conditions.

Major Critiques:
(1) As described above, the study depends on the artificial system to generate massive amount of cytosolic DNA. The authors should devise the experiments by which they can argue the biological/pathological significance of the relocation of UBTF and POLR1A. This is a must experiment for the publication in high-influential journals. dsDNA virus infection may be a good experiment.

(2) Does Ara-C treatment activate cGAS-STING pathway?

(3) Figure2A: the staining protocol of dsDNA should be described. Why can dsDNA in the cytoplasm only be stained (not genomic DNA/mitochondrial DNA)?

(4) Figure 3B: STING KO cells and cGAS KO cells should be examined. This reviewer would like to see the difference of the remaining dsDNA between WT cells and STING KO cells with Ara-C treatment. cGAMP stimulation is OK, but only supports the previous conclusion that STING pathway activates DNA-phagy. Therefore, not essential.

Minor critique:
p4: "indispensible" reads "indispensable".

Referee #2:

In this manuscript, the authors uncovered a novel role of cytosolic DNA in control of rDNA transcription. Moreover, they found that elimination of cytosolic DNA by STING-induced autophagy is sufficient to restore rDNA transcription activity. Mechanistically, they demonstrated that cytosolic DNA retains rDNA transcription machinery, which could be specifically abolished by STING-induced autophagy. These findings not only reveal a novel function of cytosolic DNA and expand the biological function of STING-induced non-canonical autophagy, but also offer insights into the pathogenesis of various related human disorders, such as infectious diseases, aging, and potentially unveil novel therapeutic avenues. Overall, this is an exciting study with compelling evidence, and the experiments are generally well executed and controlled to support the conclusions. I only have several concerns, which may help strengthen their conclusions and expand its influence.

Specific comments:

1. In Fig. 1, the authors examined the binding affinities of different cytosolic DNA fragments with rDNA transcription machinery, including UBTF and POLR1A. They should analyze the relative affinities statistically.
2. The authors found that longer DNA co-precipitated higher amount of UBTF and POLR1A. To further validate this finding, they should check whether longer cytosolic DNA exerts stronger inhibitory effect on rDNA transcription.
3. Many groups have demonstrated the primordial functions of the cGAS-STING pathway in autophagy, which is independent of TBK1. Supporting this notion, the authors showed that both STING and autophagy proteins are indeed required for the restoration of nuclear levels of UBTF and POLR1A. In addition to autophagy proteins, they should also examine whether the recovery of rDNA transcription activity is also dependent on STING and whether TBK1 is involved in this process.
4. The pre-rRNA level in the cell is highly dynamic, which may not only be determined by production but also regulated by

degradation. The authors should provide evidence to show whether cytosolic DNA and STING-induced autophagy affect the degradation of the pre-rRNA and discuss the findings accordingly.
5. In addition to autophagy, lysosome biogenesis induced by STING is essential for the elimination of cytosolic DNA, which is dependent on STING's proton channel activity. The authors should examine whether the proton channel activity is also required for STING to restore rDNA transcription activity.

Referee #3:

In this study, Yinfeng Xu et al., aimed to propose a DNA-sensing pathway-independent biological processes of cytosolic DNA. They proposed that cytosolic DNA sequesters the RNA polymerase components UBTF and POLR1A, thereby inhibiting rDNA synthesis in the nucleus and ultimately impairing cell proliferation. Additionally, autophagy appears to play a role in clearing cytosolic DNA, thereby restoring nuclear levels of UBTF and POLR1A and mitigating these effects. While the study provides substantial evidence supporting this hypothesis, several concerns should be addressed prior to publication.

Major Comments:
1. In Figure 1G, the direct binding of POLR1A to DNA should be examined in parallel with UBTF as a control to validate whether POLR1A also binds DNA.
2. Statistical analysis should be provided for the data presented in Figures 2A, 2B, and 2G. Furthermore, a cellular fractionation assay would strengthen the conclusions drawn from Figure 2G.
3. Given that both WIPI2 and ATG5 are involved in both canonical and non-canonical DNA-induced autophagy, it is essential to use additional canonical autophagy-related genes (e.g., FIP200 or ULK1/2) in the functional assays (Figures 3, 4, and 5) to clarify which autophagy pathway is involved.
4. For Figure 3E, it should be confirmed whether STING overexpression alone in 293T cells activates downstream signaling (e.g., TBK1/IRF3 phosphorylation) and autophagy induction to ensure the experimental system is functional.
5. In Figure 3F, the level of cytosolic UBTF in ATG5 KO cells appears lower than in WT cells. Since impaired autophagy should lead to DNA accumulation and potentially more UBTF sequestration, this result seems counterintuitive and requires explanation. Could the ATG5 KO cells be sick, aged, or have altered baseline signaling? Please confirm this finding by repeating the experiment with a different ATG-deficient cell line to ensure the phenotype is due to autophagy loss and not a clone-specific artifact.
6. The data in Figure 3 do not sufficiently demonstrate whether cytosolic DNA, UBTF, and SQSTM1 can form a tripartite complex. The observed interactions could be explained either by a direct three-part complex mediated by DNA, or by separate binary complexes-such as DNA-UBTF or DNA-SQSTM1.
7. The authors suggest that SQSTM1 competes with UBTF for DNA binding. It would be informative to examine whether SQSTM1 and UBTF colocalize in cells or not under relevant conditions.
8. If SQSTM1 is involved in cytoplasmic DNA recognition, does its knockout impair DNA clearance and consequently enhance the inhibition of rDNA synthesis and cell proliferation?

Minor Comments:
p-values should be explicitly indicated for the WT group in Figures 4G and 4H.

Referee #1:

In this manuscript, the authors proposed that cytosolic DNA retains UBTF and POLR1A in the cytosol, thereby inhibiting rDNA transcription that occurs in the nucleolus. Their findings are potentially interesting, however, many issues need to be cleared. What is critically missing in the study is demonstration of the biological/pathological significance of the relocation of UBTF and POLR1A to the cytosol. The authors routinely use ISD transfection and/or Ara-C, surely producing the tons of cytosolic DNA, which never occurs in physiological conditions.

Re: Thank you for your positive evaluation and constructive suggestion to our study. According to your suggestion, we have performed additional experiments to further investigate the pathological significance by utilizing HSV-1 infection. This model has been widely used to cause a rapid accumulation of significant amounts of viral DNA within the cytosol of host cells (PMID: 31332347; 30842662; 39689715).

Major Critiques:

(1) As described above, the study depends on the artificial system to generate massive amount of cytosolic DNA. The authors should devise the experiments by which they can argue the biological/pathological significance of the relocation of UBTF and POLR1A. This is a must experiment for the publication in high-influential journals. dsDNA virus infection may be a good experiment.

Re: According to your suggestion, we have carried out more experiments using HSV-1 infection in cGAS-STING- and autophagy-deficient cells. We found that HSV-1 infection induced UBTF and POLR1A relocation, inhibited rDNA transcription, and decreased protein synthesis in cells. Importantly, activation of STING-induced autophagy abolished all these effects, which is consistent with the previous observations that STING-induced autophagy facilitates the clearance of HSV-1 (PMID: 30842662; 37471002). These new data, added as Figs. 2I, 2J, 3L, 4J, 4K, 4L, and 5C, are consistent with and strengthen our conclusions.

(2) Does Ara-C treatment activate cGAS-STING pathway?

Re: Ara-C is known to generate cytosolic DNA to activate the cGAS-STING pathway (PMID: 25284779; 36559204), albeit with lower intensity than the direct STING agonist cGAMP (PMID: 36559204). Consistent with this, we observed that Ara-C elevated *Ifnb1* mRNA level, but to a lesser extent than cGAMP, and all these effects were abolished by *Sting* knockout. While Ara-C activates the cGAS-STING pathway, a robust and short-term cGAMP stimulation is well-established to significantly enhance the clearance of cytosolic DNA through autophagy-dependent manner (PMID: 30842662; 36872914). The new data have been added as Fig. EV2C in the revised manuscript.

(3) Figure2A: the staining protocol of dsDNA should be described. Why can dsDNA in the cytoplasm only be stained (not genomic DNA/mitochondrial DNA)?

Re: To achieve specific immunostaining of cytosolic DNA, we employed a low-permeabilization buffer (0.01% Triton X-100) that allows the dsDNA antibody to pass through plasma membrane, but not mitochondrial membrane or nuclear envelope (PMID: 31727239). To validate the specificity of this approach and the antibody, we performed a comparative experiment using a standard high-permeabilization buffer (0.1% Triton X-100). Consistent with the method's specificity, nuclear DNA was not stained under low-permeabilization conditions. In contrast, high permeabilization resulted in the staining of both nuclear DNA and increased cytosolic DNA, the latter likely representing mitochondrial DNA. These control data are added as Appendix Fig. S1, and the detailed staining protocol has been provided in the Methods section.

(4) Figure 3B: STING KO cells and cGAS KO cells should be examined. This reviewer would like to see the difference of the remaining dsDNA between WT cells and STING KO cells with Ara-C treatment. cGAMP stimulation is OK, but only supports the previous conclusion that STING pathway activates DNA-phagy. Therefore, not essential.

Re: According to your request, we performed cytosolic DNA clearance experiments using *Sting* knockout cells. Obviously, *Sting* knockout further increased Ara-C treatment-induced cytosolic DNA accumulation, and abolished the clearance of cytosolic DNA induced by subsequent cGAMP stimulation. To examine the role of cGAS, we used its inhibitor G150. While G150 inhibited Cy3-ISD clearance and cGAMP promoted it, G150 did not block the clearance in cGAMP-treated cells. This verifies that the autophagic clearance of cytosolic DNA depends specifically on downstream STING activation. These new data have been added as Fig. 3D-G in the revised manuscript.

Minor critique:

p4: "indispensible" reads "indispensable".

Re: Thank you, we have corrected this spelling error and carefully revised the manuscript.

Referee #2:

In this manuscript, the authors uncovered a novel role of cytosolic DNA in control of rDNA transcription. Moreover, they found that elimination of cytosolic DNA by STING-induced autophagy is sufficient to restore rDNA transcription activity. Mechanistically, they demonstrated that cytosolic DNA retains rDNA transcription machinery, which could be specifically abolished by STING-induced autophagy. These findings not only reveal a novel function of cytosolic DNA and expand the biological function of STING-induced non-canonical autophagy, but also offer

insights into the pathogenesis of various related human disorders, such as infectious diseases, aging, and potentially unveil novel therapeutic avenues. Overall, this is an exciting study with compelling evidence, and the experiments are generally well executed and controlled to support the conclusions. I only have several concerns, which may help strengthen their conclusions and expand its influence.

Re: Thank you for your nice summary, positive evaluation, and constructive suggestion to our study.

Specific comments:

1. In Fig. 1, the authors examined the binding affinities of different cytosolic DNA fragments with rDNA transcription machinery, including UBTF and POLR1A. They should analyze the relative affinities statistically.

Re: The relative affinities have been analyzed statistically, and the statistical data have been added as Fig. 1F,H.

2. The authors found that longer DNA co-precipitated higher amount of UBTF and POLR1A. To further validate this finding, they should check whether longer cytosolic DNA exerts stronger inhibitory effect on rDNA transcription.

Re: We have performed this experiment and found that longer cytosolic DNA indeed exhibited stronger inhibitory effect on rDNA transcription. The new data have been added as Fig. 4B.

3. Many groups have demonstrated the primordial functions of the cGAS-STING pathway in autophagy, which is independent of TBK1. Supporting this notion, the authors showed that both STING and autophagy proteins are indeed required for the restoration of nuclear levels of UBTF and POLR1A. In addition to autophagy proteins, they should also examine whether the recovery of rDNA transcription activity is also dependent on STING and whether TBK1 is involved in this process.

Re: Using *Sting* and *TBK1* knockout cells, we examined rDNA transcription upon HSV-1 infection, a model known to rapidly accumulate cytosolic viral DNA (PMID: 31332347; 30842662; 39689715). HSV-1 infection decreased *47S* rRNA level, and this decrease was rescued by cGAMP treatment. Crucially, the restorative effect of cGAMP was abolished in *Sting* but not *TBK1* knockout cells. These results confirm that activation of STING rather than TBK1 is required to trigger the autophagic clearance of cytosolic DNA and restore rDNA transcription activity. The new data have been added as Fig. 4J,K.

4. The pre-rRNA level in the cell is highly dynamic, which may not only be determined by production but also regulated by degradation. The authors should provide evidence to show whether cytosolic DNA and STING-induced autophagy affect the degradation of the pre-rRNA and discuss the findings accordingly.

Re: Thank you for your constructive suggestion. To test whether STING-induced autophagy directly degrades the pre-rRNA, we measured the half-life time of the *Ets1* fragment that is derived from the *47S* rRNA (PMID: PMID: 9211976; 23412654), in *Sting* and *Wipi2* knockout cells. rDNA transcription has been demonstrated to be rapidly inhibited by a high dose of actinomycin D (Act-D) (PMID: PMID: 9211976; 23412654). After the treatment, we measured the levels of *Ets1* and *Gapdh* mRNA, and the latter was used as the internal control. *Gapdh* mRNA level remained nearly unaffected at different time points. By contrast, the half-life time of *Ets1* was less than 10 min, which is consistent with the previous observations (PMID: PMID: 9211976; 23412654). However, the half-life time of *Ets1* remained nearly unchanged in wild-type, *Sting*, and *Wipi2* knockout cells. Moreover, ISD transfection had no obvious effect on the half-life time of *Ets1* in these cells. These data suggest that STING-induced autophagy does not degrade *47S* rRNA. The new data have been added as Fig. EV5B.

5. In addition to autophagy, lysosome biogenesis induced by STING is essential for the elimination of cytosolic DNA, which is dependent on STING's proton channel activity. The authors should examine whether the proton channel activity is also required for STING to restore rDNA transcription activity.

Re: We have examined whether the proton channel activity is required for STING to restore rDNA transcription activity by using compound 53 (C53), a special STING agonist that can block STING’s proton channel activity (PMID: 37535724; 38177926). By using this chemical, we found that block of STING’s proton channel activity significantly decreased the recovery of *47S* rRNA level led by cGAMP treatment in cells with ISD transfection. These data suggest that STING’s proton channel activity is required for the recovery of rDNA transcription activity in cells with cytosolic DNA. The proton channel activity is necessary for STING to stimulate lysosome biogenesis (PMID: 39423796; 39689715), which is essential for the efficient clearance of cytosolic DNA (PMID: 39689715). Our results are consistent with these previous findings and further support the conclusion that clearance of the cytosolic DNA is required for the recovery of rDNA transcription activity. The new data have been added as Fig. EV5A.

Referee #3:

In this study, Yinfeng Xu et al., aimed to propose a DNA-sensing pathway-independent biological processes of cytosolic DNA. They proposed that cytosolic DNA sequesters the RNA polymerase components UBTF and POLR1A, thereby inhibiting rDNA synthesis in the nucleus and ultimately impairing cell proliferation. Additionally, autophagy appears to play a role in clearing cytosolic DNA, thereby restoring nuclear levels of UBTF and POLR1A and mitigating these effects. While the study provides substantial evidence supporting this hypothesis, several concerns should be addressed prior to publication.

Re: Thank you for your nice summary, positive evaluation, and constructive suggestion to our study.

Major Comments:

1. In Figure 1G, the direct binding of POLR1A to DNA should be examined in parallel with UBTF as a control to validate whether POLR1A also binds DNA.

Re: Thank you for your suggestion, we have examined the direct binding of POLR1A to DNA using recombinant protein *in vitro*. The data showed that POLR1A can also interact with DNA, but the affinity was much lower compared to UBTF. Based on this, we further checked the binding of UBTF to DNA in cells with *POLR1A* knockdown. We found that knockdown of *POLR1A* did not affect the UBTF-DNA binding in cells. Given that our previous data show that knockdown of *UBTF* decreases POLR1A-DNA binding, all the data together suggest that it is UBTF rather than POLR1A acting as the key factor to mediate the association of cytosolic DNA with the RNA Pol I transcription machinery in cells. The new data have been included as Figs. 1I and EV1 in the revised manuscript.

2. Statistical analysis should be provided for the data presented in Figures 2A, 2B, and 2G. Furthermore, a cellular fractionation assay would strengthen the conclusions drawn from Figure 2G.

Re: The statistical analysis has been performed and the data have been provided as Fig. 2B,H in the revised manuscript. We also carried out cellular fractionation assay as requested and obtained consistent results. The new data have been added as Fig. EV2F,G.

3. Given that both WIPI2 and ATG5 are involved in both canonical and non-canonical DNA-induced autophagy, it is essential to use additional canonical autophagy-related genes (e.g., FIP200 or ULK1/2) in the functional assays (Figures 3, 4, and 5) to clarify which autophagy pathway is involved.

Re: According to your request, we performed additional experiments using *Ulk1/2* knockout cells. We found that *Ulk1/2* knockout did not substantially affect cGAMP-induced recovery of UBTF and POLR1A localization, rDNA transcription, and protein synthesis in cells with Ara-C treatment or HSV-1 infection. These results are consistent with prior findings that ULK1/2 complex is dispensable for STING-induced autophagic clearance of cytosolic DNA and HSV-1 virus (PMID: 30842662; PMID: 37471002). The new data have been added as Figs. EV3F, 4L, and 5C.

4. For Figure 3E, it should be confirmed whether STING overexpression alone in 293T cells activates downstream signaling (e.g., TBK1/IRF3 phosphorylation) and autophagy induction to ensure the experimental system is functional.

Re: We have examined the cGAS-STING signaling and autophagy induction in HEK293T cells stably expressing STING-HA. Analysis of TBK1, IRF3, and STING phosphorylation confirmed that STING expression alone does not activate the cGAS-STING pathway. However, cGAMP treatment successfully activated this signaling in these cells but not in wild-type (WT) controls, consistent with prior findings that STING expression can reconstitute this signaling pathway in WT HEK293T cells, in which the endogenous *STING* is silenced (PMID: 28720717; 30842662). While STING has been reported to control energy stress-induced autophagy (PMID: 35510944), our assessment of autophagosome formation and autophagic flux under basal conditions revealed that STING expression alone does not substantially affect basal autophagy in HEK293T cells. These new results ensure that the experimental system we used in this study is functional. The data have been added as Fig. EV3A-E.

5. In Figure 3F, the level of cytosolic UBTF in ATG5 KO cells appears lower than in WT cells. Since impaired autophagy should lead to DNA accumulation and potentially more UBTF sequestration, this result seems counterintuitive and requires explanation. Could the ATG5 KO cells be sick, aged, or have altered baseline signaling? Please confirm this finding by repeating the experiment with a different ATG-deficient cell line to ensure the phenotype is due to autophagy loss and not a clone-specific artifact.

Re: To address your concern, we re-performed the experiment and confirmed that wild-type (WT) and *Atg5* knockout cells with Ara-C treatment exhibited similar protein levels of UBTF and POLR1A in the cytoplasm. To further verify this observation, we performed additional cellular fractionation assays using different autophagy-deficient cells with Ara-C treatment or HSV-1 infection and obtained consistent results. Moreover, we found that knockout of *Atg5* or *Wipi2* but not *Ulk1/2*, the latter is not required for STING-induced autophagy (PMID: 30842662; PMID: 37471002), abolished the relocation of UBTF and POLR1A in cGAMP-treated cells. The new data have been included as Figs. 3J-L, and EV3F.

As expected, autophagy-deficient cells treated with Ara-C accumulated more cytosolic DNA. However, the levels of cytosolic UBTF and POLR1A in these cells remained comparable to those in WT cells. Since autophagy-deficient cells are known to accumulate much higher level of SQSTM1—an autophagy receptor that competes with UBTF for binding to cytosolic DNA—the elevated SQSTM1 likely exerts a stronger inhibitory effect on Ara-C-induced retention of UBTF and POLR1A in the cytoplasm. This competition may explain why differences in UBTF and POLR1A relocation between WT and autophagy-deficient cells under these conditions were relatively mild. In contrast, activation of STING-induced autophagy by cGAMP specifically cleared cytosolic DNA in WT cells, but not in autophagy-deficient cells. This selective clearance in WT cells resulted in a pronounced difference in the cytoplasmic retention of UBTF and POLR1A. We have elaborated on this point in the Discussion section in the revised manuscript.

6. The data in Figure 3 do not sufficiently demonstrate whether cytosolic DNA, UBTF, and SQSTM1 can form a tripartite complex. The observed interactions could be explained either by a direct three-part complex mediated by DNA, or by separate binary complexes-such as DNA - UBTF or DNA - SQSTM1.

Re: Thank you for pointing out this issue, we have re-interpreted our results in the revised manuscript.

7. The authors suggest that SQSTM1 competes with UBTF for DNA binding. It would be informative to examine whether SQSTM1 and UBTF colocalize in cells or not under relevant conditions.

Re: Thank you for your suggestion, we have performed additional cell image experiments. We did not observe the co-localization of SQSTM1 and UBTF in cells with or without cGAMP treatment. Moreover, we found that cGAMP stimulation could decrease UBTF-DNA co-localization but increase SQSTM1-DNA co-localization in cells. These data further support the notion that SQSTM1 and UBTF compete for cytosolic DNA binding. The new data have been added as Fig. EV4H,I. We have also discussed this finding in the Discussion section in the revised manuscript.

8. If SQSTM1 is involved in cytoplasmic DNA recognition, does its knockout impair DNA clearance and consequently enhance the inhibition of rDNA synthesis and cell proliferation?

Re: We have checked cytosolic DNA clearance using *SQSTM1* knockout cells. Clearly, knockout of *SQSTM1* leaded to a significant accumulation of cytosolic DNA in Ara-C-treated cells, and which could not be abolished by cGAMP stimulation. These new data have been added as Fig. EV4K,L.

Actually, our previous study has demonstrated that accumulated SQSTM1 in autophagy-deficient cells can elevate basal mTORC1 activity, thereby enhancing rDNA transcription, protein synthesis, and cell proliferation (PMID: 34612149). Although SQSTM1 is a well-known autophagy receptor protein in autophagy process, it plays multiple roles beyond autophagy. For example, SQSTM1 can function as a hub protein integrating several signaling pathways, including mTORC1 (PMID: 30499183). Therefore, there seem to be several different mechanisms underlying the inhibition of rDNA transcription, protein synthesis, and cell proliferation in *SQSTM1* knockout cells.

Minor Comments:

p-values should be explicitly indicated for the WT group in Figures 4G and 4H.

Re: Thank you, p-values in these figures have been indicated.

Dear Prof. Wan,

Thank you for submitting your manuscript for consideration by the EMBO Journal. It has now been seen again by all three referees whose comments are shown below.

As you can see from the reports, all three referees find the manuscript to be improved and are generally favorable toward publication in The EMBO Journal. There is one remaining concern raised by referee #1 that would require an additional experiment, which we consider reasonable and likely to strengthen the manuscript. We therefore invite you to revise the manuscript to address this remaining point.

Thank you for the opportunity to consider your work for publication. I look forward to your revision.

Yours sincerely,

Cornelius Schneider, PhD
Editor
The EMBO Journal
c.schneider@embojournal.org

Instructions for preparing your revised manuscript:

Read our guidance for manuscript revisions and related editorial policies: https://link.springer.com/journal/44318/submission-guidelines#cms-Revised-submissions

Please make sure you upload a letter of response to the referees' comments together with the revised manuscript.

Please also check that the title and abstract of the manuscript are brief, yet explicit, even to non-specialists.

When assembling figures, please refer to our figure preparation guideline in order to ensure proper formatting and readability in print as well as on screen:
https://media.springernature.com/original/springer-cms/rest/v1/content/27825798/data/v1

At EMBO Press we ask authors to provide source data for the main manuscript figures. You will receive a separate email with instructions for providing source data with your revised manuscript, including how to upload and organize the files.

IMPORTANT: When you send the revision we will require
- a point-by-point response to the referees' comments, with a detailed description of the changes made (as a word file).
- a word file of the manuscript text.
- individual production quality figure files (one file per figure)
- a complete author checklist
- Expanded View files (replacing Supplementary Information)
- a Reagents and Tools Table as part of the Methods section

Please remember: Digital image enhancement is acceptable practice, as long as it accurately represents the original data and conforms to community standards. If a figure has been subjected to significant electronic manipulation, this must be noted in the figure legend or in the 'Materials and Methods' section. The editors reserve the right to request original versions of figures and the original images that were used to assemble the figure.

We realize that it is difficult to revise to a specific deadline. In the interest of protecting the conceptual advance provided by the work, we recommend a revision within 3 months (3rd May 2026). Please discuss the revision progress ahead of this time with the editor if you require more time to complete the revisions. Use the link below to submit your revision:

https://emboj.msubmit.net/cgi-bin/main.plex

Referee #1:

In this revised manuscript, the authors mostly addressed to the original critiques. The new data showing the relocation of UBTF/POLR1A upon HSV infection are neat, strengthening the significance of the present study. This reviewer has one critique on the new HSV infection experiment.

A HSV infection brings HSV-derived dsDNA into the cytosol, and at the same time, activates the STING pathway/STING-dependent DNAphagy, indicating a trade-off between dsDNA appearance (by HSV infection) and dsDNA clearance (by STING-dependent DNAphagy). Therefore, this reviewer is keen to know the activation status of STING during the HSV infection. The timing of cGAS-STING activation during the HSV infection may be the key to understanding this conundrum (for example, STING-dependent DNA-phagy will take place later time points after HSV infection). Or, the HSV infection may not fully activate the STING pathway in the authors' experimental settings, therefore, the cGAMP stimulation is further required to induce the DNAphagy. The authors would like also to discuss the results in Figure 3H/I.

Referee #2:

The authors have satisfactorily addressed all my concerns with rigorous data and clear logic. Moreover, they have significantly strengthened their core model by employing compelling genetic evidence, including autophagy-deficient systems with specific knockouts of canonical and non-canonical autophagy genes. This solidly demonstrates that STING-mediated clearance of cytosolic DNA occurs via non-canonical autophagy, which in turn relieves the suppression of rDNA transcription. The physiological significance of this study is further enhanced by linking this mechanism to a disease context through the use of HSV-1 infection.

Referee #3:

My concerns have been addressed.

Referee #1:

In this revised manuscript, the authors mostly addressed to the original critiques. The new data showing the relocation of UBTF/POLR1A upon HSV infection are neat, strengthening the significance of the present study. This reviewer has one critique on the new HSV infection experiment.

Re: Thank you for your kind support and positive evaluation on our revised manuscript.

A HSV infection brings HSV-derived dsDNA into the cytosol, and at the same time, activates the STING pathway/STING-dependent DNAphagy, indicating a trade-off between dsDNA appearance (by HSV infection) and dsDNA clearance (by STING-dependent DNAphagy). Therefore, this reviewer is keen to know the activation status of STING during the HSV infection. The timing of cGAS-STING activation during the HSV infection may be the key to understanding this conundrum (for example, STING-dependent DNA-phagy will take place later time points after HSV infection). Or, the HSV infection may not fully activate the STING pathway in the authors' experimental settings, therefore, the cGAMP stimulation is further required to induce the DNAphagy. The authors would like also to discuss the results in Figure 3H/I.

Re: Thank you for your criticism. We agree with you that there seems to be a trade-off between cytosolic DNA appearance and cytosolic DNA clearance. We think the activation status of STING may determine the efficiency of cytosolic DNA clearance. Actually, we have checked the activation status of the cGAS-STING signaling by measuring *Ifnb1* mRNA levels in cells with Ara-C treatment or ISD infection. Although both Ara-C treatment and ISD transfection could activate the cGAS-STING pathway, the STING agonist cGAMP activated STING more strongly. The data are shown in Fig. EV2B,C. Several previous studies have shown that cGAMP stimulation is required for the efficient clearance of cytosolic DNA via STING-induced autophagy (PMID: 30842662; 36872914; 37999993). Therefore, a strong activation of STING seems to greatly enhance the autophagic clearance of cytosolic DNA. To meet your request, we performed additional experiments and checked the cGAS-STING signaling in cells with HSV-1 infection. In contrast to the strong activation by cGAMP, HSV-1 infection activated STING to a much weaker degree, as indicated by TBK1 and STING phosphorylation. These new data have been added as Fig. EV3G. In addition, we have discussed the results in Fig. 3H,I in the revised manuscript.

Referee #2:

The authors have satisfactorily addressed all my concerns with rigorous data and clear logic. Moreover, they have significantly strengthened their core model by employing compelling genetic evidence, including autophagy-deficient systems with specific knockouts of canonical and non-canonical autophagy genes. This solidly demonstrates that STING-mediated clearance of cytosolic DNA occurs via non-canonical autophagy, which in turn relieves the suppression of rDNA transcription. The physiological

significance of this study is further enhanced by linking this mechanism to a disease context through the use of HSV-1 infection.

Re: Thank you for your positive evaluation on our revised manuscript.

Referee #3:

My concerns have been addressed.

Re: Thank you and we are happy that you are satisfied with our revised manuscript.

2nd Revision - Editorial Decision 27th Feb 2026

Dear Prof. Wan,

Thank you for submitting a revised version of your manuscript. Your study has now been seen by referee #1, who finds that their previous concerns have been addressed and now recommends publication of the manuscript. There remain only a few mainly editorial points that have to be addressed before I can extend formal acceptance of the manuscript:

- As we are switching from a free-text author contribution statement towards a more formal statement based on Contributor Role Taxonomy (CRediT) terms, please remove the present Author Contribution section and instead specify each author's contribution(s) directly in the Author Information page of our submission system during upload of the final manuscript. See https://casrai.org/credit/ for more information.

- REFERENCES: DOIs should only be used for preprints and datasets that have not been published yet

- FIGURE CALLOUTS: missing for Appendix Tables S1-S4

- Please provide suggestions for a short 'blurb' text prefacing and summing up the conceptual aspect of the study in two sentences (max. 250 characters), followed by 3-5 one-sentence 'bullet points' with brief factual statements of key results of the paper; they will form the basis of an editor-written 'Synopsis' accompanying the online version of the article. Please also provide an altered synopsis image, making sure that the aspect ratio conforms to our website's format - it should be exactly 550 pixels wide and between 300-600 pixels high.

- During our routine image integrity checks, we observed that the images within the Figure set appear pixelated under analysis. This is often a result of converting original 16-bit TIFF files to RGB format for publication. While this is not inherently problematic, it can give the impression of image alteration to critical readers. To address this, please upload all source data using the 16-bit captured image. This will enable us to confirm the integrity of the complete figure set and enhance transparency for readers.

- Figure Legends - Comments:

- Please note that the exact p values are not provided in the legends of figures 1F, 2 B, D, F, H, J; 3C, E, G; 4A, B, D, E, G, H, I, J, K, L; 5B, C, E, F, G; EV2 C, G; EV4 I, L; EV5 A

- Please note that information related to n is missing in the legends of figures S2 A, B

- Please note that the error bars are not defined in the legends of figures S2 A, B

Please let me know if you have any questions regarding any of these points. You can use the link below to upload the revised files.
Thank you again for giving us the chance to consider your manuscript for The EMBO Journal. I look forward to receiving the final version.
With best regards,
Cornelius Schneider

____
Cornelius Schneider, PhD
Editor | The EMBO Journal
c.schneider@embojournal.org

Please refer to our figure preparation guideline in order to ensure proper formatting and readability in print as well as on screen:

https://link.springer.com/journal/44318/submission-guidelines#cms-Figure-and-data-presentation

Use the link below to submit your revision:

https://emboj.msubmit.net/cgi-bin/main.plex

Referee #1:

My concers have been addressed neatly. The manuscript has been much improved and is ready for publication.

Editor:

As we are switching from a free-text author contribution statement towards a more formal statement based on Contributor Role Taxonomy (CRediT) terms, please remove the present Author Contribution section and instead specify each author's contribution(s) directly in the Author Information page of our submission system during upload of the final manuscript. See https://casrai.org/credit/ for more information.

Re: The Author Contribution has been removed and the CRediT has been added.

REFERENCES: DOIs should only be used for preprints and datasets that have not been published yet.

Re: The DOIs of the references have been deleted.

FIGURE CALLOUTS: missing for Appendix Tables S1-S4.

Re: Appendix Tables S1-S4 have been referenced in the Methods and the Reagents and tools table.

Please provide suggestions for a short 'blurb' text prefacing and summing up the conceptual aspect of the study in two sentences (max. 250 characters), followed by 3-5 one-sentence 'bullet points' with brief factual statements of key results of the paper; they will form the basis of an editor-written 'Synopsis' accompanying the online version of the article. Please also provide an altered synopsis image, making sure that the aspect ratio conforms to our website's format - it should be exactly 550 pixels wide and between 300-600 pixels high.

Re: Provided.

During our routine image integrity checks, we observed that the images within the Figure set appear pixelated under analysis. This is often a result of converting original 16-bit TIFF files to RGB format for publication. While this is not inherently problematic, it can give the impression of image alteration to critical readers. To address this, please upload all source data using the 16-bit captured image. This will enable us to confirm the integrity of the complete figure set and enhance transparency for readers.

Re: The figures have been uploaded using the 16-bit captured image.

Figure Legends - Comments:

Please note that the exact p values are not provided in the legends of figures 1F, 2 B, D, F, H, J; 3C, E, G; 4A, B, D, E, G, H, I, J, K, L; 5B, C, E, F, G; EV2 C, G; EV4 I, L; EV5 A.

Re: The exact p values have been provided in the figures.

Please note that information related to n is missing in the legends of figures S2 A, B.

Re: Provided.

Please note that the error bars are not defined in the legends of figures S2 A, B.

Re: Defined.

Referee #1:

My concerns have been addressed neatly. The manuscript has been much improved and is ready for publication.

Re: We are happy that you are satisfied with the revised manuscript.

Dear Prof. Wan,

I am pleased to inform you that your manuscript has been accepted for publication in the EMBO Journal.

Your manuscript will be processed for publication by EMBO Press. It will be copy edited and you will receive page proofs prior to publication. Please note that you will be contacted by Springer Nature Author Services to complete licensing and payment information.

You may qualify for financial assistance for your publication charges - either via a Springer Nature fully open access agreement or an EMBO initiative. Check your eligibility: https://link.springer.com/journal/44318/how-to-publish-with-us

Should you be planning a Press Release on your article, please get in contact with embo_production@springernature.com as early as possible in order to coordinate publication and release dates.

If you have any questions, please do not hesitate to contact the Editorial Office. Thank you for your contribution to The EMBO Journal.

Yours sincerely,

Cornelius Schneider, PhD
Editor
The EMBO Journal
c.schneider@embojournal.org

*****
Please note that it is The EMBO Journal policy for the transcript of the editorial process (containing referee reports and your response letters) to be published as an online supplement to each paper. If you should prefer removal of any referee-only figures included in the point-by-point response(s), e.g. because they may still be used for future publication or because they have been reproduced from published work by others, please do let us know immediately via response email.
More information is available here: https://link.springer.com/partners/embo-press/editorial-policies#Peer%20review

** Click here to be directed to your login page: https://emboj.msubmit.net

EMBO Press Author Checklist

Corresponding Author Name: Wei Wan	
Journal Submitted to: The EMBO Journal	
Manuscript Number: EMBOJ-2025-121731	

USEFUL LINKS FOR COMPLETING THIS FORM
The EMBO Journal - Author Guidelines
EMBO Reports - Author Guidelines
Molecular Systems Biology - Author Guidelines
EMBO Molecular Medicine - Author Guidelines

Reporting Checklist for Life Science Articles (updated January
This checklist is adapted from Materials Design Analysis Reporting (MDAR) Checklist for Authors. MDAR establishes a minimum set of requirements in transparent reporting in the life sciences (see Statement of Task: 10.31222/osf.io/9sm4x). Please follow the journal's guidelines in preparing your
Please note that a copy of this checklist will be published alongside your article.

Abridged guidelines for figures
1. Data
The data shown in figures should satisfy the following conditions:
- the data were obtained and processed according to the field's best practice and are presented to reflect the results of the experiments in an accurate and unbiased manner.
- ideally, figure panels should include only measurements that are directly comparable to each other and obtained with the same assay.
- plots include clearly labeled error bars for independent experiments and sample sizes. Unless justified, error bars should not be shown for technical
- if n<5, the individual data points from each experiment should be plotted. Any statistical test employed should be justified.
- Source Data should be included to report the data underlying figures according to the guidelines set out in the authorship guidelines on Data

2. Captions
Each figure caption should contain the following information, for each panel where they are relevant:
- a specification of the experimental system investigated (eg cell line, species name).
- the assay(s) and method(s) used to carry out the reported observations and measurements.
- an explicit mention of the biological and chemical entity(ies) that are being measured.
- an explicit mention of the biological and chemical entity(ies) that are altered/varied/perturbed in a controlled manner.
- the exact sample size (n) for each experimental group/condition, given as a number, not a range;
- a description of the sample collection allowing the reader to understand whether the samples represent technical or biological replicates (including how many animals, litters, cultures, etc.).
- a statement of how many times the experiment shown was independently replicated in the laboratory.
- definitions of statistical methods and measures:
  - common tests, such as t-test (please specify whether paired vs. unpaired), simple χ2 tests, Wilcoxon and Mann-Whitney tests, can be unambiguously identified by name only, but more complex techniques should be described in the methods section;
  - are tests one-sided or two-sided?
  - are there adjustments for multiple comparisons?
  - exact statistical test results, e.g., P values = x but not P values < x;
  - definition of 'center values' as median or average;
  - definition of error bars as s.d. or s.e.m.

Please complete ALL of the questions below.
Select "Not Applicable" only when the requested information is not relevant for your study.

Materials

Newly Created Materials	Information included in the manuscript?	In which section is the information available? (Reagents and Tools Table, Materials and Methods, Figures, Data Availability Section)
New materials and reagents need to be available; do any restrictions apply?	Not Applicable	

Antibodies	Information included in the manuscript?	In which section is the information available? (Reagents and Tools Table, Materials and Methods, Figures, Data Availability Section)
For **antibodies** provide the following information:<br>- Commercial antibodies: RRID (if possible) or supplier name, catalogue number and or/clone number<br>- Non-commercial: RRID or citation	Yes	Reagents and Tools Table

DNA and RNA sequences	Information included in the manuscript?	In which section is the information available? (Reagents and Tools Table, Materials and Methods, Figures, Data Availability Section)
**Short novel DNA or RNA including primers, probes**: provide the sequences.	Yes	Appendix Table

Cell materials	Information included in the manuscript?	In which section is the information available? (Reagents and Tools Table, Materials and Methods, Figures, Data Availability Section)
**Cell lines:** Provide species information, strain. Provide accession number in repository **OR** supplier name, catalog number, clone number, and/**OR** RRID.	Yes	Reagents and Tools Table
**Primary cultures:** Provide species, strain, sex of origin, genetic modification status.	Not Applicable	
Report if the cell lines were recently **authenticated** (e.g., by STR profiling) and tested for mycoplasma contamination.	Yes	Methods

Experimental animals	Information included in the manuscript?	In which section is the information available? (Reagents and Tools Table, Materials and Methods, Figures, Data Availability Section)
**Laboratory animals or Model organisms:** Provide species, strain, sex, age, genetic modification status. Provide accession number in repository **OR** supplier name, catalog number, clone number, **OR** RRID.	Not Applicable	
**Animal observed in or captured from the field:** Provide species, sex, and age where possible.	Not Applicable	
Please detail **housing and husbandry conditions**.	Not Applicable	

Plants and microbes	Information included in the manuscript?	In which section is the information available? (Reagents and Tools Table, Materials and Methods, Figures, Data Availability Section)
**Plants:** provide species and strain, ecotype and cultivar where relevant, unique accession number if available, and source (including location for collected wild specimens).	Not Applicable	
**Microbes:** provide species and strain, unique accession number if available, and source.	Yes	Methods

Human research participants	Information included in the manuscript?	In which section is the information available? (Reagents and Tools Table, Materials and Methods, Figures, Data Availability Section)
If collected and within the bounds of privacy constraints report on age, sex and gender or ethnicity for all study participants.	Not Applicable	

Core facilities	Information included in the manuscript?	In which section is the information available? (Reagents and Tools Table, Materials and Methods, Figures, Data Availability Section)
If your work benefited from core facilities, was their service mentioned in the acknowledgments section?	Yes	Acknowledgements

Design

Study protocol	Information included in the manuscript?	In which section is the information available? (Reagents and Tools Table, Materials and Methods, Figures, Data Availability Section)
If study protocol has been **pre-registered, provide DOI in the manuscript**. For clinical trials, provide the trial registration number **OR** cite DOI.	Yes	Methods
Report the **clinical trial registration number** (at ClinicalTrials.gov or equivalent), where applicable.	Not Applicable	

Laboratory protocol	Information included in the manuscript?	In which section is the information available? (Reagents and Tools Table, Materials and Methods, Figures, Data Availability Section)
Provide DOI OR other citation details if **external detailed step-by-step protocols** are available.	Yes	Methods

Experimental study design and statistics	Information included in the manuscript?	In which section is the information available? (Reagents and Tools Table, Materials and Methods, Figures, Data Availability Section)
Include a statement about **sample size** estimate even if no statistical methods were used.	Yes	Figure legends
Were any steps taken to minimize the effects of subjective bias when allocating animals/samples to treatment (e.g. **randomization procedure**)? If yes, have they been described?	Not Applicable	
Include a statement about **blinding** even if no blinding was done.	Not Applicable	
Describe **inclusion/exclusion criteria** if samples or animals were excluded from the analysis. Were the criteria pre-established?<br><br>If sample or data points were omitted from analysis, report if this was due to attrition or intentional exclusion and provide justification.	Not Applicable	
For every figure, are **statistical tests** justified as appropriate? Do the data meet the assumptions of the tests (e.g., normal distribution)? Describe any methods used to assess it. Is there an estimate of variation within each group of data? Is the variance similar between the groups that are being statistically compared?	Yes	Figure legends and Methods

Sample definition and in-laboratory replication	Information included in the manuscript?	In which section is the information available? (Reagents and Tools Table, Materials and Methods, Figures, Data Availability Section)
In the figure legends: state number of times the experiment was **replicated** in laboratory.	Yes	Figure legends
In the figure legends: define whether data describe **technical or biological replicates**.	Yes	Figure legends

Ethics

Ethics	Information included in the manuscript?	In which section is the information available? (Reagents and Tools Table, Materials and Methods, Figures, Data Availability Section)
Studies involving **human participants**: State details of **authority granting ethics approval** (IRB or equivalent committee(s), provide reference number for approval.	Not Applicable	
Studies involving **human participants**: Include a statement confirming that **informed consent** was obtained from all subjects and that the experiments conformed to the principles set out in the WMA Declaration of Helsinki and the Department of Health and Human Services Belmont Report.	Not Applicable	
Studies involving **human participants:** For publication of **patient photos**, include a statement confirming that consent to publish was obtained.	Not Applicable	
Studies involving experimental **animals**: State details of **authority granting ethics approval** (IRB or equivalent committee(s), provide reference number for approval. Include a statement of compliance with ethical regulations.	Not Applicable	
Studies involving **specimen and field samples:** State if relevant **permits** obtained, provide details of authority approving study; if none were required, explain why.	Not Applicable	

Dual Use Research of Concern (DURC)	Information included in the manuscript?	In which section is the information available? (Reagents and Tools Table, Materials and Methods, Figures, Data Availability Section)
Could your study fall under dual use research restrictions? Please check biosecurity documents and list of **select agents and toxins** (CDC): https://www.selectagents.gov/sat/list.htm	Not Applicable	
If you used a select agent, is the security level of the lab appropriate and reported in the manuscript?	Not Applicable	
If a study is subject to dual use research of concern regulations, is the name of the **authority granting approval and reference number** for the regulatory approval provided in the manuscript?	Not Applicable	

Reporting

The MDAR framework recommends adoption of discipline-specific guidelines, established and endorsed through community initiatives. Journals have their own policy about requiring specific guidelines and recommendations to complement MDAR.

Adherence to community standards	Information included in the manuscript?	In which section is the information available? (Reagents and Tools Table, Materials and Methods, Figures, Data Availability Section)
State if relevant guidelines or checklists (e.g., **ICMJE, MIBBI, ARRIVE, PRISMA**) have been followed or provided.	Not Applicable	
For **tumor marker prognostic studies,** we recommend that you follow the **REMARK** reporting guidelines (see link list at top right). See author guidelines, under 'Reporting Guidelines'. Please confirm you have followed these guidelines.	Not Applicable	
For **phase II and III randomized controlled trials**, please refer to the **CONSORT** flow diagram (see link list at top right) and submit the CONSORT checklist (see link list at top right) with your submission. See author guidelines, under 'Reporting Guidelines'. Please confirm you have submitted this list.	Not Applicable	

Data Availability

Data availability	Information included in the manuscript?	In which section is the information available? (Reagents and Tools Table, Materials and Methods, Figures, Data Availability Section)
Have **primary datasets** been deposited according to the journal's guidelines (see 'Data Deposition' section) and the respective accession numbers provided in the Data Availability Section?	Not Applicable	
Were **human clinical and genomic datasets** deposited in a public access-controlled repository in accordance to ethical obligations to the patients and to the applicable consent agreement?	Not Applicable	
Are **computational models** that are central and integral to a study available without restrictions in a machine-readable form? Were the relevant accession numbers or links provided?	Not Applicable	
If publicly available data were reused, provide the respective **data citations in the reference list**.	Not Applicable	
